# Racial disparities in characteristics and outcomes of patients undergoing mitral transcatheter edge-to-edge repair

**DOI:** 10.3389/fcvm.2023.1111714

**Published:** 2023-03-02

**Authors:** Alon Shechter, Danon Kaewkes, Moody Makar, Vivek Patel, Ofir Koren, Keita Koseki, Aum Solanki, Manvir Dhillon, Takashi Nagasaka, Sabah Skaf, Tarun Chakravarty, Raj R. Makkar, Robert J. Siegel

**Affiliations:** ^1^Department of Cardiology, Smidt Heart Institute, Cedars-Sinai Medical Center, Los Angeles, CA, United States; ^2^Department of Cardiology, Rabin Medical Center, Petah Tikva, Israel; ^3^Faculty of Medicine, Tel Aviv University, Tel Aviv, Israel; ^4^Department of Medicine, Faculty of Medicine, Khon Kaen University, Khon Kaen, Thailand; ^5^Rappaport Faculty of Medicine, Technion – Israel Institute of Technology, Haifa, Israel; ^6^Department of Cardiovascular Medicine, The University of Tokyo, Tokyo, Japan; ^7^Department of Cardiovascular Medicine, Gunma University Graduate School of Medicine, Maebashi, Gunma, Japan; ^8^David Geffen School of Medicine, University of California, Los Angeles, Los Angeles, CA, United States

**Keywords:** mitral regurgitation, mitral transcatheter edge-to-edge repair, transcatheter mitral valve repair, MitraClip, racial disparities

## Abstract

**Background:**

There are scarce data regarding the post-mitral transcatheter edge-to-edger repair (TEER) course in different racial groups.

**Objective:**

To assess the impact of race on outcomes following TEER for mitral regurgitation (MR).

**Methods:**

This is a single-center, retrospective analysis of consecutive TEER procedures performed during 2013–2020. The primary outcome was the composite of all-cause mortality or heart failure (HF) hospitalizations along the first postprocedural year. Secondary outcomes included individual components of the primary outcome, New York Heart Association (NYHA) class, MR grade, and left ventricular mass index (LVMi).

**Results:**

Out of 964 cases, 751 (77.9%), 88 (9.1%), 68 (7.1%), and 57 (5.9%) were whites, blacks, Asians, and Hispanics, respectively. At baseline, non-whites and blacks were younger and more likely be female, based in lower socioeconomic areas, not fully insured, diagnosed with functional MR, and affected by biventricular dysfunction. Intra-procedurally, more devices were implanted in blacks. At 1-year, non-whites (vs. whites) and blacks (vs. non-blacks or whites) experienced higher cumulative incidence of the primary outcome (32.9% vs. 22.5%, *p* = 0.002 and 38.6% vs. 23.4% or 22.5%, *p* = 0.002 or *p* = 0.001, respectively), which were accounted for by hospitalizations in the functional MR sub-cohort (*n* = 494). NYHA class improved less among blacks with functional MR. MR severity and LVMi equally regressed in all groups. White race (HR 0.62, 95% CI 0.39–0.99, *p* = 0.047) and black race (HR 2.07, 95% CI 1.28–3.35, *p* = 0.003) were independently associated with the primary outcome in functional MR patients only.

**Conclusion:**

Mitral TEER patients of different racial backgrounds exhibit major differences in baseline characteristics. Among those with functional MR, non-whites and blacks also experience a less favorable 1-year clinical outcome.

## Introduction

Transcatheter edge-to-edge repair (TEER) is an effective therapy for both functional (1) and primary (2) forms of mitral regurgitation (MR). Consequently, it has a central role in the management of these conditions according to the most recent North American (3) and European (4) practice guidelines. Lately, there has been increased recognition of the interaction between race/ethnicity and numerous disease states and therapies, including percutaneous structural heart interventions (5, 6). While not explicitly assessed in the pivotal randomized controlled trials, racial disparities affecting the mitral TEER arena were found in real-world reports that examined short-term, namely in-hospital, outcomes (7–10). Although plausible in theory, the association between race and longer-term course following mitral TEER has not been explored. Using a contemporary, large database, we evaluated the characteristics and 1-year clinical and echocardiographic outcomes of patients undergoing mitral TEER according to race. In addition, we assessed whether race was an independent predictor for mortality or heart failure (HF) hospitalizations.

## Methods

### Study population and outcomes

Our study is the product of an observational analysis of the Cedars-Sinai Medical Center (CSMC) registry of consecutive mitral TEER procedures performed on adult patients between January 1st, 2013 and December 31st, 2020. Constructed using an electronic medical chart platform (CS-Link™, Epic, Verona, WI, United States), the registry contained information regarding demographic background, medical conditions, therapeutics, electrocardiographic, laboratory and imaging studies, procedures, hospitalizations, and deaths – all as entered by medical providers and state authorities in real-time. While insurance coverage data were taken directly from patient files, income and level of education were estimated based on the 2020 United States (US) Census Report (11) according to address zip codes.

Patients included in the study underwent an isolated, first-ever mitral TEER. Formal assessment was carried per protocol at baseline, hospital discharge, 1-month and 1-year post-procedure. Stratification was made according to self-reported racial background. The primary outcome was the combined 1-year rate of all-cause mortality or HF hospitalizations. Secondary outcomes consisted of the separate endpoints of death and HF hospitalizations, as well as the achievement of New York Heart Association (NYHA) functional class I-II and/or MR grade of mild or moderate and less at 1-month and 1-year. Left ventricular mass index (LVMi) by echocardiography, as a surrogate of remodeling, was also assessed. One-month adverse events were defined as any of the following: cardiac tamponade, cardiac arrest, myocardial infarction, stroke, transient ischemic attack, Mitral Valve Academic Research Consortium (MVARC) bleeding, and vascular complications. All medical diagnoses were made in accordance with accepted criteria, such as those of the MVARC (12).

This project complied with the Declaration of Helsinki and was approved by the Cedars-Sinai Institutional Review Board (IRB), which waived the need for informed consent.

### Procedural and echocardiographic aspects

Mitral TEER followed a Heart Team discussion that included at least one interventional cardiologist, one cardiac surgeon, one echocardiologist, and one HF specialist. Patient-, disease-, and institution-related aspects, as well as perceived risks and benefits according to the best scientific evidence at the time, were all considered in the decision process. All procedures used the MitraClip™ system (Abbott Vascular Inc., Santa Clara, CA, United States) and were performed under general anesthesia, *via* a femoral venous access, and with echocardiographic and fluoroscopic guidance. Monitoring by simultaneous right heart catheterization (RHC) was utilized as well.

Echocardiograms were carried out pre-, intra-, and post-procedure using the EPIQ ultrasound system (Philips, Amsterdam, Netherlands) and the PICOM365 software for post-test processing (SciImage, Los Altos, CA, United States), and conformed to the relevant American Society of Echocardiography (ASE) guidelines (13–15). MR severity was assessed using integration of qualitative and semi-quantitative measures, whenever applicable. MR Etiology was determined according to the valve leaflet morphology, as visualized on the intraprocedural transesophageal echocardiogram (TEE). Pulmonary venous flow pattern (PVFP) was evaluated on TEE by a pulsed-wave (PW) Doppler beam placed within 1 cm of the PV ostia. Normalization in the PV flow after clip deployment required the emergence of a peak systolic (S) to peak diastolic (D) velocity ratio of ≥1 on either side. LVMi calculation was applied on transthoracic echocardiograms (TTEs) using the ASE formula. Global right ventricular (RV) function was determined by qualitative assessment.

### Statistical analysis

The study cohort was split into 4 groups based on race – white, black, Asian, and Hispanic. For each group, variables were reported as frequencies and percentages, medians and interquartile ranges (IQR), or means and standard deviations, as appropriate. Inter-group comparisons incorporated two racial groups at a time (mostly whites vs. non-whites, blacks vs. non-blacks, Asians vs. non-Asians, Hispanics vs. non-Hispanics, and whites vs. blacks) and utilized Pearson’s Chi-Square, Fisher’s exact, Student’s *t*, or Mann–Whitney *U* tests. Evaluation of change over time in variables within each group was based on paired-sample t, Wilcoxon, or McNemaer tests.

The risk for death and/or HF hospitalizations as a function of race was graphically displayed according to the Kaplan–Meier method, with comparisons of cumulative event-free survival times across strata by the Log-Rank test. To identify associations between baseline and procedural factors and the primary outcome, a multivariable Cox regression analysis was employed that integrated variables demonstrating a value of *p* of <0.1 on a preliminary univariable model. Parameters included in this first step were chosen based their perceived prognostic implication, as judged by clinical reasoning, published data (16, 17), and results of the comparison between the various racial groups.

Lastly, descriptive and survival analyses were repeated on two matched cohorts that were created by means of propensity score matching – the first comparing whites and non-whites and the second comparing blacks and non-blacks. In each cohort, cases were matched according to the probability of belonging to either white or black racial group, in a 1:1 fashion, and by using a match tolerance of ≤0.1. A multivariable binary logistic regression was employed for calculating this probability, which included baseline parameters of perceived and/or proved prognostic implication regarding the primary outcome: age, sex, regular/full insurance, median yearly income and percentage of adults with academic degrees according to area of residence, chronic obstructive pulmonary disease (COPD), peripheral arterial disease (PAD), blood hemoglobin level, estimated glomerular filtration rate, no use of renin angiotensin system (RAS) inhibitors, high-dose diuretics (i.e., ≥80 mg of furosemide per day or use of ≥2 diuretics excluding mineralocorticoid receptor antagonists (MRAs)), NYHA class, functional MR, left ventricular ejection fraction (LVEF), and low tricuspid annular plane systolic excursion (TAPSE) to pulmonary arterial systolic pressure (PASP) ratio according to the total cohort’s median.

Cases with missing data were censored from the relevant analyses. A two-sided value of *p* of <0.05 defined statistical significance. All analyses were performed using SPSS, version 24 (IBM Corporation, Armonk, NY, United States).

## Results

### Baseline characteristics of the study population

A total of 964 patients were identified that underwent an isolated, first-time mitral TEER at CSMC between 2013 and 2020. Of these, 751 (77.9%), 88 (9.1%), 68 (7.1%), and 57 (5.9%) were whites, blacks, Asians, and Hispanics, respectively. Among patients aged 65 or 75 years and over, the figures were 667 (81.3%) or 503 (84.7%) for whites, 51 (6.2%) or 27 (4.5%) for blacks, 60 (7.3%) or 41 (6.9%) for Asians, and 42 (5.1%) or 23 (3.9%) for Hispanics. The follow-up duration was 468 (IQR, 106–1,034) days. Baseline, 1-month, and 1-year echocardiograms were performed on day 19 (IQR, 5–46) before, day 33 (IQR, 29–36) after, and day 370 (IQR, 351–403) following the intervention, respectively.

Pre-procedural clinical characteristics of the four racial groups are summarized in Table 1. A significant inter-racial variation in demographics and comorbidities was evident. Notably, whites were the oldest [median age 80 (IQR, 72–87) years] and most often male (*n* = 469, 62.5%), as well as the most fully insured (*n* = 677, 90.4%), highest paid, and most academically educated group. In blacks, on the other hand, the median age, proportion of males to females, full coverage percentage, median household income, and fraction of adults with academic degrees were lowest [67 (IQR, 58–77) years, 40 (45.5%) males, 67 (76.1%) with regular insurance]. Blacks were more likely to have a cardiac implantable electronic device (CIED) or non-ischemic cardiomyopathy. Indices of functional and symptomatic status, including the NYHA class, the Kansas City Cardiomyopathy Questionnaire (KCCQ) 12 score, and the 6-min walk test distance, were comparable; B-type natriuretic peptide (BNP) levels, however, were highest within the black group and lowest in whites. Surgical and percutaneous risk were not significantly different. While most of the medical treatment prior to mitral TEER was comparable, whites were prescribed less MRAs and more oral anticoagulants and blacks received more often a hydralazine-nitrates combination.

**Table 1:** Baseline clinical characteristics of the total cohort according to race.

					*p*-value				
	Whites (*N* = 751)	Blacks (*N* = 88)	Asians (*N* = 68)	Hispanics (*N* = 57)	Whites vs. non-whites	Blacks vs. non-blacks	Asians vs. non-Asians	Hispanics vs. non-Hispanics	Whites vs. blacks
**Demographic details**									
Age (years)	80 (72–87)	67 (58–77)	78 (68–84)	73 (63–82)	**<0.001**	**<0.001**	0.291	**0.002**	**<0.001**
Sex Male	469 (62.5)	40 (45.5)	37 (54.4)	32 (56.1)	**0.003**	**0.004**	0.333	0.544	**0.002**
**Insurance**									
None	5 (0.7)	0 (0.0)	0 (0.0)	1 (1.8)	0.746	0.436	0.498	0.264	0.442
Low-income	67 (8.9)	21 (23.9)	14 (20.6)	24 (42.1)	**<0.001**	**0.002**	0.058	**<0.001**	**<0.001**
Regular / full	677 (90.4)	67 (76.1)	54 (79.4)	32 (56.1)	**<0.001**	**0.004**	0.088	**<0.001**	**<0.001**
Median yearly household income* (K USD)	84.9 (64.4–103.0)	58.3 (52.3–72.2)	78.9 (59.3–97.8)	70.6 (55.1–95.1)	**<0.001**	**<0.001**	0.334	**0.018**	**<0.001**
Percentage of adults with academic degree*	44.0 (28.1–61.9)	24.0 (16.0–35.0)	39.3 (27.0–48.1)	28.8 (18.9–46.4)	**<0.001**	**<0.001**	0.174	**<0.001**	**<0.001**
**Comorbidities**									
Obesity (Body mass index ≥30 kg/m^2^)	127 (16.9)	21 (23.9)	6 (8.8)	12 (21.1)	0.633	0.083	0.057	0.429	0.105
Diabetes mellitus	175 (23.4)	28 (31.8)	27 (39.7)	25 (43.9)	**<0.001**	0.239	**0.011**	**0.002**	0.081
Hypertension	627 (83.5)	75 (86.2)	49 (72.1)	50 (87.7)	0.627	0.428	**0.011**	0.345	0.515
Smoking history	32 (4.3)	3 (3.4)	0 (0.0)	3 (5.3)	0.341	0.783	0.103	0.488	0.702
Previous MI, PCI, or CABG	330 (43.9)	26 (29.5)	28 (41.2)	33 (57.9)	0.421	**0.006**	0.719	**0.021**	**0.010**
Prior stroke or transient ischemic attack (TIA)	99 (13.2)	13 (14.8)	13 (19.1)	4 (7.0)	0.733	0.688	0.150	0.146	0.678
Peripheral arterial disease (PAD)	60 (8.0)	8 (9.1)	4 (5.9)	4 (7.0)	0.816	0.662	0.524	0.801	0.723
Atrial fibrillation / flutter	424 (56.5)	33 (37.5)	31 (45.6)	26 (45.6)	**<0.001**	**0.002**	0.185	0.229	**0.001**
Chronic obstructive pulmonary disease (COPD)	101 (13.4)	17 (19.3)	6 (8.8)	4 (7.0)	0.769	0.080	0.262	0.151	0.134
Anemia+	459 (61.1)	63 (71.6)	41 (60.3)	44 (77.2)	**0.026**	0.079	0.636	**0.022**	0.055
Stage ≥III chronic kidney disease	534 (73.0)	61 (70.1)	59 (88.1)	39 (69.6)	0.423	0.443	**0.005**	0.492	0.575
**Heart failure indices**									
**New York Heart Association (NYHA) Class**									
II	53 (7.1)	3 (3.4)	4 (5.9)	1 (1.8)	0.081	0.238	0.876	0.254	0.195
III	310 (41.4)	32 (36.4)	24 (35.3)	30 (52.6)	0.813	0.346	0.315	0.068	0.375
IV	385 (51.4)	53 (60.2)	40 (58.8)	26 (45.6)	0.235	0.118	0.263	0.299	0.111
Kansas city cardiomyopathy questionnaire 12 score	39.1 (18.8–62.0)	36.5 (13.0–52.1)	38.0 (17.7–69.3)	26.3 (6.8–51.0)	0.092	0.370	0.751	**0.033**	0.279
6-Minute walk test distance (m)	244 (122–335)	183 (61–305)	274 (187–366)	219 (122–354)	0.865	0.092	0.106	0.803	0.106
Serum B-type natriuretic peptide (pg/mL)	466 (221–1,104)	1,180 (494–2,088)	615 (284–1,470)	486 (255–1,553)	**<0.001**	**<0.001**	0.219	0.535	**<0.001**
**Risk status**									
STS score for mitral valve repair	5.5 (3.0–8.7)	5.9 (2.1–9.0)	6.2 (3.7–10.4)	5.5 (2.1–9.8)	0.929	0.313	0.136	0.828	0.365
MitraScore	3 (2–4)	4 (3–4)	3 (2–4)	4 (3–4)	0.622	0.256	0.378	0.663	0.280
**Treatment**									
Medications									
Beta Blockers	514 (68.4)	68 (77.3)	42 (61.8)	43 (75.4)	0.344	0.085	0.169	0.292	0.089
Renin angiotensin system (RAS) inhibitors	367 (48.9)	49 (55.7)	33 (48.5)	27 (47.4)	0.552	0.25	0.985	0.754	0.226
Mineralocorticoid receptor antagonists (MRAs)	135 (18.0)	29 (33.0)	12 (17.6)	18 (31.6)	**0.002**	**0.002**	0.597	**0.026**	**0.001**
**Loop diuretics**
Frequency	557 (74.2)	73 (83.0)	55 (80.9)	39 (68.4)	0.207	0.074	0.253	0.229	0.071
Furosemide-equivalent dose (mg/day)	40 (20–80)	40 (40–80)	40 (40–40)	60 (40–80)	0.085	0.067	0.086	**0.006**	0.058
Anti-arrhythmics	159 (21.2)	20 (22.7)	14 (20.6)	11 (19.3)	0.982	0.710	0.901	0.79	0.741
Hydralazine + nitrates	16 (2.1)	7 (8.0)	1 (1.5)	2 (3.5)	**0.039**	**0.006**	0.533	0.685	**0.006**
Oral anticoagulants	355 (47.3)	35 (39.8)	30 (44.1)	16 (28.1)	**0.017**	0.281	0.849	**0.007**	0.182
**Cardiac implantable electronic device (CIED)**
Total	237 (31.6)	49 (55.7)	16 (23.5)	17 (29.8)	0.057	**<0.001**	0.082	0.589	**<0.001**
Pacemaker	95 (12.6)	9 (10.2)	5 (7.4)	3 (5.3)	0.061	0.669	0.265	0.123	0.514
Implantable cardioverter defibrillator (ICD)	40 (5.3)	17 (19.3)	2 (2.9)	6 (10.5)	**0.001**	**<0.001**	0.311	0.268	**<0.001**
CRT/defibrillator (CRT/D)	102 (13.6)	23 (26.1)	9 (13.2)	8 (14.0)	0.059	**0.002**	0.718	0.879	**0.002**

Data are presented as number (percentage) or median (interquartile range), where appropriate. Figures in bold denote statistical significance. *Per zip code. + Anemia was defined as a blood hemoglobin of <13 mg/dL in men or < 12 mg/dL in women. CABG, coronary bypass artery grafting; CRT, cardiac resynchronization therapy; GFR, glomerular filtration rate; MI, myocardial infarction; PCI, percutaneous coronary intervention; STS, Society of Thoracic Surgeons; USD, United States Dollars.

Echocardiographic parameters are presented in detail in Table 2. Functional MR was observed more commonly in blacks (*n* = 71, 80.7%) and Hispanics (*n* = 40, 70.2%), while primary MR – in whites (*n* = 399, 53.1%). Apart from effective regurgitant orifice area (EROA), which was lowest in blacks, most indices of MR severity, as well as left atrial volume index (LAVi), were comparable across the various racial groups. Blacks displayed the lowest LVEF and the highest prevalence of ≥moderate RV dysfunction. By contrast, whites exhibited the highest LVEF and RV-pulmonary arterial (PA) coupling values and harbored the lowest frequencies of significant RV dysfunction and tricuspid regurgitation (TR). Baseline LVMi was lower among whites and higher among blacks and Hispanics.

**Table 2:** Baseline echocardiographic data of the total cohort according to race.

					*P*-value				
	Whites (*N* = 751)	Blacks (*N* = 88)	Asians (*N* = 68)	Hispanics (*N* = 57)	Whites vs. non-whites	Blacks vs. non-blacks	Asians vs. non-Asians	Hispanics vs. non-Hispanics	Whites vs. blacks
**Mitral valve**									
Mitral regurgitation etiology					**<0.001**	**<0.001**	0.333	**0.003**	**<0.001**
Functional	352 (46.9)	71 (80.7)	31 (45.6)	40 (70.2)					
Primary	399 (53.1)	17 (19.3)	37 (54.4)	17 (29.8)					
Mitral regurgitation severity									
Moderate–severe	152 (20.3)	14 (15.9)	5 (7.4)	12 (21.1)	0.058	0.429	**0.011**	0.693	0.324
Severe	589 (78.8)	73 (83.0)	63 (92.6)	44 (77.2)	0.068	0.482	**0.007**	0.570	0.369
Mitral regurgitation PISA EROA (cm^2^)	0.37 (0.27–0.50)	0.34 (0.25–0.40)	0.38 (0.27–0.42)	0.30 (0.22–0.43)	**0.020**	0.260	0.543	**0.044**	0.167
Mitral regurgitation PISA RVol (mL)	51.8 (36.9–69.5)	48.4 (36.8–60.8)	50.2 (41.0–66.8)	44.8 (31.2–56.1)	0.119	0.387	0.813	0.053	0.306
Transmitral mean pressure gradient (TMPG) (mmHg)	3 (2–4)	3 (2–4)	3 (2–4)	3 (2–4)	0.396	0.595	0.174	0.525	0.544
≥Moderate mitral annulus calcification (MAC)	83 (11.1)	4 (4.5)	5 (7.4)	5 (8.8)	0.055	0.071	0.441	0.738	0.058
**Left heart**									
Left ventricular ejection fraction (LVEF) (%)	55 (34–64)	29 (18–45)	58 (33–66)	40 (25–60)	**<0.001**	**<0.001**	0.122	**0.041**	**<0.001**
Left ventricular end-systolic diameter (LVESD) (cm)	3.7 (3.0–4.8)	5.3 (3.9–6.2)	3.8 (3.0–4.9)	4.2 (3.4–5.6)	**<0.001**	**<0.001**	0.398	0.054	**<0.001**
Left ventricular mass index, ASE formula (gr/m^2^)	124.7 (98.3–151.3)	131.3 (110.4–153.6)	130.0 (107.4–168.8)	136.7 (111.5–158.8)	**<0.001**	**0.047**	0.099	**0.041**	**0.019**
Left atrial volume index (LAVi) (cm^3^/m^2^)	57.2 (43.2–75.0)	52.0 (40.2–72.8)	56.8 (46.1–71.8)	60.5 (50.1–75.2)	0.916	0.147	0.897	0.140	0.199
**Right heart**									
≥Moderate right ventricular dysfunction	115 (17.9)	26 (34.2)	15 (24.6)	13 (22.8)	**0.003**	**0.001**	0.380	0.617	**0.001**
≥Moderate–severe tricuspid regurgitation	144 (19.3)	25 (28.4)	23 (34.3)	24 (42.1)	**<0.001**	0.164	**0.016**	**<0.001**	**0.043**
**Right ventricular (RV)-pulmonary arterial (PA) coupling**									
Tricuspid annular plane systolic excursion (TAPSE) (mm)	17 (14–20)	16 (14–22)	17 (13–20)	15 (13–20)	0.698	0.716	0.942	0.259	0.809
Pulmonary arterial systolic pressure (PASP) (mmHg)	44 (34–57)	53 (35–64)	45 (33–60)	48 (37–63)	**0.006**	**0.022**	0.573	0.149	**0.013**
TAPSE/PASP (mm/mmHg)	0.39 (0.27–0.56)	0.34 (0.23–0.50)	0.35 (0.29–0.54)	0.31 (0.22–0.44)	**0.018**	0.129	0.925	**0.025**	0.085

Data are presented as number (percentage) or median (interquartile range), where appropriate. Figures in bold denote statistical significance. ASE, American Society of Echocardiography; EROA, effective regurgitant orifice area; PISA, proximal isovelocity surface area; RVol, regurgitant volume.

### Procedural details and short-term results

Supplementary Table 1 summarizes aspects pertaining to the mitral TEER procedure itself and its immediate and short-term results. Whites were less likely to present to the intervention with acute decompensated heart failure or to require hemodynamic support. Intra-procedurally, black patients had more devices implanted. Clipping was mostly applied to the A2P2 segment, with no major differences in total procedure or fluoroscopy times across the races. While immediate reduction in MR severity to mild or less and PVFP normalization on either side were similarly achieved in the four racial groups, fewer black and Asian patients maintained an up to mild MR upon hospital discharge (*n* = 61, 69.3% and *n* = 45, 69.2%, respectively). Further, blacks experienced the highest rate of in-hospital blood transfusion or any 1-month adverse events (*n* = 17, 19.3%), the lengthiest duration of hospitalization [3 (IQR, 1–9) days], and the highest 1-month transmitral mean pressure gradient (TMPG) [5 (IQR, 3–6) mmHg].

### Mortality and heart failure hospitalizations

**Figure 1:**
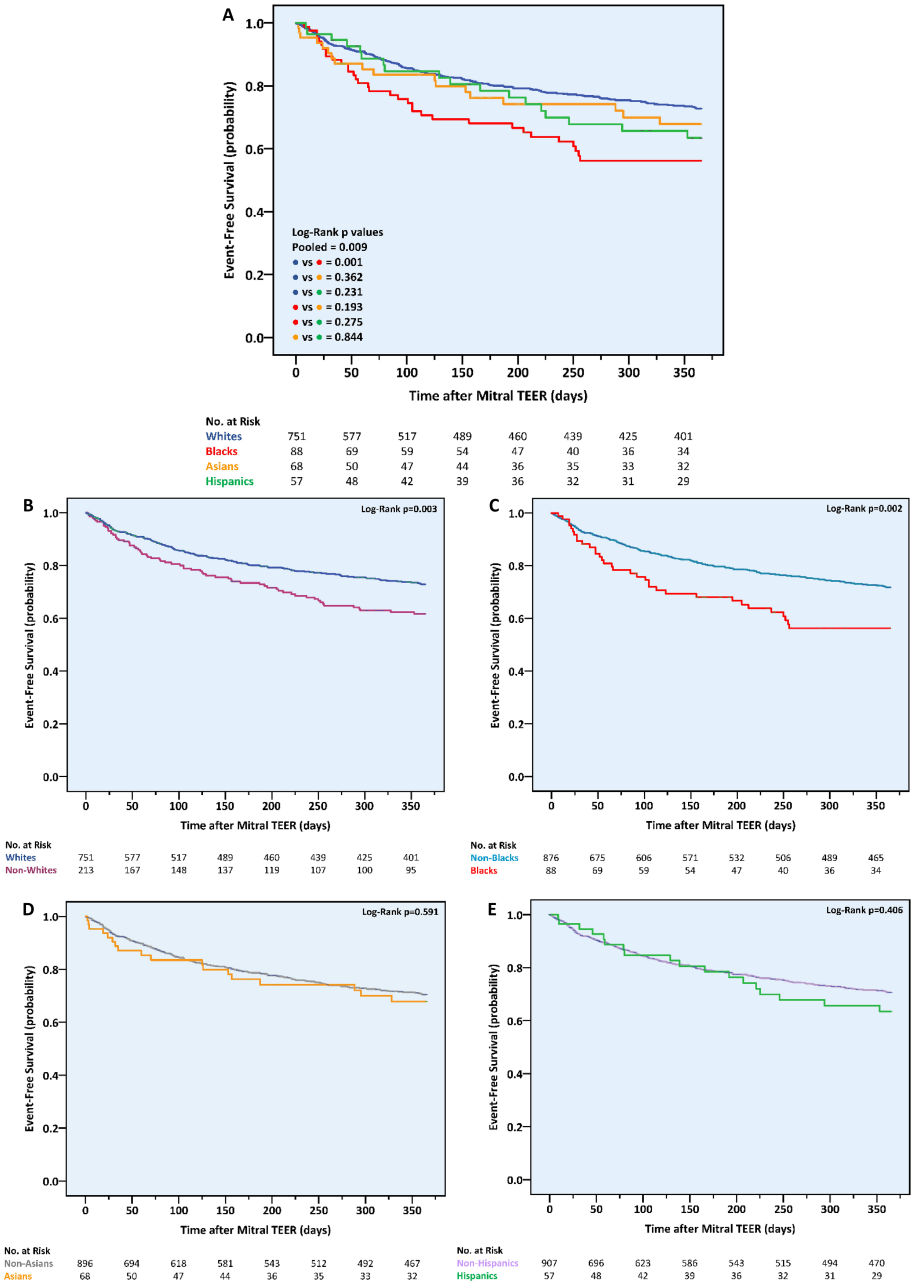
One-year cumulative incidence of the combined outcome of all-cause mortality or heart failure hospitalizations following mitral transcatheter edge-to-edge repair according to race. **(A)** All races, **(B)** whites vs. non-whites, **(C)** blacks vs. non-blacks, **(D)** Asians vs. non-Asians, **(E)** Hispanics vs. non-Hispanics. TEER, transcatheter edge-to-edge repair.

By the end of the first postprocedural year, the primary outcome, a composite of all-cause mortality or HF hospitalizations, was experienced by a total of 239 (24.8%) patients. Considering race, it occurred earlier and more frequently in non-whites vs. whites (*n* = 70, 32.9% vs. *n* = 169, 22.5%, *p* = 0.002), blacks vs. non-blacks (*n* = 34, 38.6% vs. *n* = 205, 23.4%, *p* = 0.002), and blacks vs. whites (*p* = 0.001) (Table 3; Figure 1). Event rates in Asians (*n* = 18, 26.5%) and Hispanics (*n* = 18, 31.6%) were similar to the ones in non-Asians (*n* = 221, 24.7%, *p* = 0.740) and non-Hispanics (*n* = 221, 24.4%, *p* = 0.221), respectively. Notably, the differences in the primary outcome incidence were accounted for by hospitalizations only (Supplementary Figures 1, 2). According to a multivariable analysis, no race was found to independently predict the primary outcome, although black race did impose a trend toward a higher risk (HR 1.61, 95% CI 0.93–2.78, *p* = 0.088) (Supplementary Tables 2, 4).

**Table 3:** Outcomes and trends following mitral transcatheter edge-to-edge repair in the total cohort according to race.

					*P*-value				
	Whites (*N* = 751)	Blacks (*N* = 88)	Asians (*N* = 68)	Hispanics (*N* = 57)	Whites vs. non-whites	Blacks vs. non-blacks	Asians vs. non-Asians	Hispanics vs. non-Hispanics	Whites vs. blacks
**Primary outcome**									
All-cause mortality or heart failure hospitalizations at 1-year	169 (22.5)	34 (38.6)	18 (26.5)	18 (31.6)	**0.002**	**0.002**	0.740	0.221	**0.001**
Event-free survival time at 1-year (days)	300 ± 5	252 ± 16	286 ± 17	285 ± 17	**0.003**	**0.002**	0.591	0.406	**0.001**
**Secondary outcomes**									
Clinical									
All-cause mortality at 1-year	84 (11.2)	15 (17.0)	8 (11.8)	8 (14.0)	0.181	0.120	0.965	0.613	0.107
Heart failure hospitalizations at 1-year	101 (13.4)	26 (29.5)	13 (19.1)	13 (22.8)	**<0.001**	**<0.001**	0.447	0.140	**<0.001**
New York Heart Association Class ≤II									
At 1-month	448 (80.1)	46 (70.8)	35 (68.6)	36 (83.7)	0.075	0.102	0.069	0.406	0.078
At 1-year	294 (78.4)	22 (56.4)	32 (91.4)	19 (67.9)	0.194	**0.001**	**0.014**	0.230	**0.002**
**Echocardiographic**									
Mitral regurgitation severity ≤mild									
At 1-month	344 (64.1)	33 (53.2)	26 (53.1)	25 (62.5)	0.059	0.126	0.171	0.969	0.094
At 1-year	158 (53.7)	17 (53.1)	12 (42.9)	12 (46.2)	0.322	0.929	0.295	0.511	0.947
Mitral regurgitation severity ≤moderate									
At 1-month	496 (94.5)	53 (89.8)	48 (98.0)	38 (95.0)	0.795	0.133	0.513	0.855	0.151
At 1-year	274 (93.2)	29 (90.6)	26 (92.9)	23 (88.5)	0.435	0.719	0.962	0.425	0.483
Left ventricular mass index (gr/m^2^)									
At 1-month	115.3 (93.5–145.7)	127.9 (105.4–176.5)	139.0 (109.5–158.6)	122.3 (106.8–150.7)	**<0.001**	**0.030**	**0.026**	0.269	**0.015**
At 1-year	110.0 (90.1–133.9)	125.7 (94.9–146.8)	120.6 (94.4–146.5)	140.5 (116.4–165.8)	**0.001**	0.192	0.521	**0.001**	0.096
Combined clinical and echocardiographic									
New York Heart Association Class ≤II or mitral regurgitation severity ≤mild									
At 1-month	505 (92.0)	54 (84.4)	40 (80.0)	39 (92.9)	**0.011**	0.080	**0.020**	0.788	**0.042**
At 1-year	324 (90.0)	29 (78.4)	32 (100.0)	21 (77.8)	0.202	**0.049**	**0.037**	0.102	**0.049**
**Trends**									
Absolute change in New York Heart Association Class									
At 1-month	−1.4 ± 0.8	−1.3 ± 0.8	−1.5 ± 0.8	−1.5 ± 0.8	0.704	0.142	0.768	0.429	0.171
At 1-year	−1.4 ± 0.9	−1.1 ± 0.7	−1.7 ± 0.7	−1.3 ± 0.9	0.424	**0.005**	**0.021**	0.485	**0.008**
*P*-value for 1-year vs. baseline	**<0.001**	**<0.001**	**<0.001**	**<0.001**	NA	NA	NA	NA	NA
Relative change in left ventricular mass index (%)									
At 1-month	−2.7 (−19.6–14.5)	5.2 (−10.7–25.8)	−1.6 (−19.1–15.7)	−3.8 (−22.6–13.0)	0.251	**0.018**	0.966	0.430	**0.021**
At 1-year	−4.3 (−21.7–17.3)	−0.2 (−20.9–22.8)	−16.3 (−30.5– −2.7)	0.1 (−17.2–27.5)	0.747	0.744	0.073	0.355	0.785

Data are presented as number (percentage), median (interquartile range), or mean ± standard deviation, where appropriate. Figures in bold denote statistical significance. NA, not applicable.

**Table 4:** Multivariable Cox proportional hazard model for the combined outcome of all-cause mortality or heart failure hospitalizations at 1 year following mitral transcatheter edge-to-edge repair.

	Total cohort				Functional mitral regurgitation			
	White race considered		Black race considered		White race considered		Black race considered	
	HR (95% CI)	*p*-value	HR (95% CI)	*p*-value	HR (95% CI)	*p*-value	HR (95% CI)	*p*-value
**Baseline clinical variables**								
Age (continuous)	0.98 (0.96–1.01)	0.064	0.99 (0.97–1.00)	0.103	NA	NA	NA	NA
Race								
White vs. non-White	0.99 (0.69–1.66)	0.776	NA	NA	0.62 (0.39–0.99)	**0.047**	NA	NA
Black vs. non-Black	NA	NA	1.61 (0.93–2.78)	0.088	NA	NA	2.07 (1.28–3.35)	**0.003**
None or low-income vs. regular / full insurance	1.04 (0.63–1.72)	0.529	1.14 (0.68–1.89)	0.625	1.15 (0.69–1.91)	0.591	1.28 (0.78–2.09)	0.332
Median yearly household income* (continuous)	1.00 (0.99–1.01)	0.259	1.00 (0.99–1.01)	0.329	NA	NA	NA	NA
Percentage of adults with academic degree* (continuous)	1.00 (0.99–1.02)	0.463	1.01 (0.99–1.02)	0.350	NA	NA	NA	NA
Diabetes mellitus	1.66 (1.07–2.56)	**0.023**	1.62 (1.05–2.50)	**0.044**	1.30 (0.82–2.07)	0.265	1.27 (0.80–2.02)	0.308
Previous MI, PCI, or CABG	0.81 (0.53–1.23)	0.322	0.88 (0.58–1.35)	0.558	NA	NA	NA	NA
Chronic obstructive pulmonary disease (COPD)	1.60 (0.94–2.71)	0.081	1.53 (0.90–2.58)	0.113	NA	NA	NA	NA
Anemia+	2.37 (1.43–3.93)	**<0.001**	2.29 (1.38–3.81)	**0.001**	3.22 (1.70–6.12)	**<0.001**	3.16 (1.66–6.01)	**<0.001**
Stage ≥III chronic kidney disease	1.36 (0.77–2.42)	0.288	1.21 (0.70–2.11)	0.497	1.27 (0.73–2.20)	0.404	1.24 (0.72–2.16)	0.438
New York Heart Association (NYHA) Class IV	1.66 (1.09–2.53)	**0.017**	1.65 (1.09–2.51)	**0.019**	1.83 (1.13–2.96)	**0.014**	1.84 (1.14–2.98)	**0.013**
Serum B-type natriuretic peptide level (continuous)	1.00 (0.98–1.01)	0.177	1.01 (0.99–1.03)	0.129	1.00 (0.99–1.01)	0.299	1.00 (0.99–1.01)	0.191
No use of renin angiotensin system (RAS) inhibitors	1.69 (1.12–2.56)	**0.013**	1.71 (1.13–2.59)	**0.010**	1.93 (1.43–2.96)	**0.012**	1.84 (1.15–2.95)	**0.012**
Furosemide-equivalent dose (continuous)	1.00 (0.99–1.04)	0.824	1.00 (0.99–1.04)	0.879	NA	NA	NA	NA
Oral anticoagulants prescription	NA	NA	NA	NA	0.90 (0.57–1.41)	0.641	0.96 (0.61–1.51)	0.859
Cardiac implantable electronic device (CIED)	1.29 (0.83–1.99)	0.262	1.22 (0.78–1.90)	0.389	NA	NA	NA	NA
**Baseline echocardiographic variables**								
Functional mitral regurgitation	1.89 (1.08–3.30)	**0.025**	1.94 (1.10–3.40)	**0.022**	NA	NA	NA	NA
Mitral regurgitation PISA EROA (continuous)	2.50 (0.79–7.69)	0.117	1.85 (0.62–5.56)	0.265	NA	NA	NA	NA
Left ventricular ejection fraction (LVEF)								
Continuous	NA	NA	NA	NA	1.01 (0.99–1.02)	0.139	1.01 (0.99–1.03)	0.103
<60%	1.60 (0.88–2.90)	0.121	1.55 (0.85–2.81)	0.152	NA	NA	NA	NA
Left ventricular end-systolic diameter (LVESD) ≥0.4 cm	2.19 (1.29–3.72)	**0.004**	2.05 (1.21–3.48)	**0.008**	NA	NA	NA	NA
Left atrial volume index (LAVi) (continuous)	1.08 (1.02–1.14)	**0.008**	1.06 (1.01–1.12)	**0.027**	1.07 (1.02–1.13)	**0.010**	1.07 (1.02–1.13)	**0.010**
≥Moderate right ventricular dysfunction	1.20 (0.75–1.93)	0.454	1.21 (0.75–1.96)	0.432	1.14 (0.69–1.88)	0.609	1.13 (0.69–1.85)	0.628
≥Moderate–severe tricuspid regurgitation	1.02 (0.65–1.61)	0.920	1.07 (0.68–1.68)	0.775	1.04 (0.64–1.69)	0.881	1.08 (0.66–1.77)	0.752
TAPSE/PASP ≤0.37 mm/mmHg (total cohort median)	1.99 (1.60–2.59)	**0.027**	1.96 (1.57–2.58)	**0.042**	1.97 (1.68–2.91)	**0.036**	1.95 (1.65–2.88)	**0.046**
Procedural variables								
Acute heart failure presentation, cardiogenic shock, hemodynamic support, or urgent procedure	1.86 (1.20–2.87)	**0.005**	1.82 (1.18–2.80)	**0.007**	1.92 (1.32–2.99)	**0.009**	1.87 (1.33–2.64)	**0.011**
Number of clips deployed (continuous)	1.39 (1.08–1.79)	**0.010**	1.39 (1.08–1.78)	**0.010**	1.38 (1.03–1.86)	**0.032**	1.31 (0.98–1.76)	0.069

*Per zip code. + Anemia was defined as a blood hemoglobin of <13 mg/dL in men or < 12 mg/dL in women. Figures in bold denote statistical significance. CABG, coronary artery bypass grafting; CI, confidence interval; EROA, effective regurgitant orifice area; HR, hazard ratio; MI, myocardial infarction; NA, not applicable; PASP, pulmonary arterial systolic pressure; PCI, percutaneous coronary intervention; PISA, proximal isovelocity surface area; TAPSE, tricuspid annular plane systolic excursion.

### Functional status

**Figure 2:**
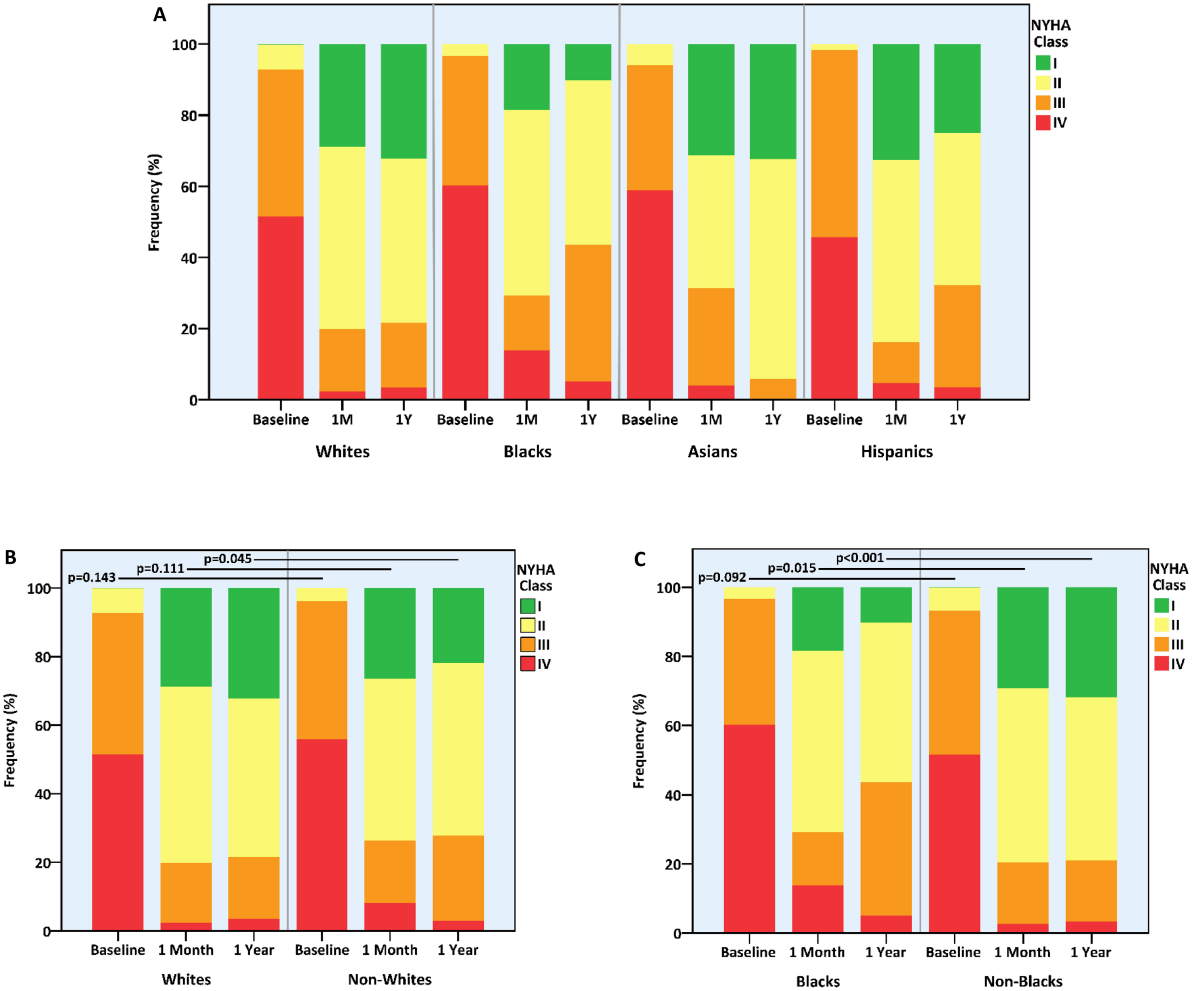
Functional status at baseline and following mitral transcatheter edge-to-edge repair according to race. **(A)** All races, **(B)** whites vs. non-whites, **(C)** blacks vs. non-blacks. NYHA, New York Heart Association.

One-year NYHA class was significantly improved compared to baseline in all groups. However, the magnitude of change was modest in blacks vs. non-blacks or whites (−1.1 ± 0.7 vs. −1.4 ± 0.9 or − 1.4 ± 0.9 classes, *p* = 0.005 and *p* = 0.008, respectively) and more prominent in Asians vs. non-Asians (−1.7 ± 0.7 vs. −1.4 ± 0.9 classes, *p* = 0.021) (Table 3). Consequently, whites and non-blacks exhibited a better functional status at 1 year compared to non-whites (*p* = 0.045) and blacks (*p* < 0.001), respectively (Figure 2). Of note, the difference between non-blacks and blacks was evident also at 1-month (*p* = 0.015).

### Mitral regurgitation severity and reverse remodeling

**Figure 3:**
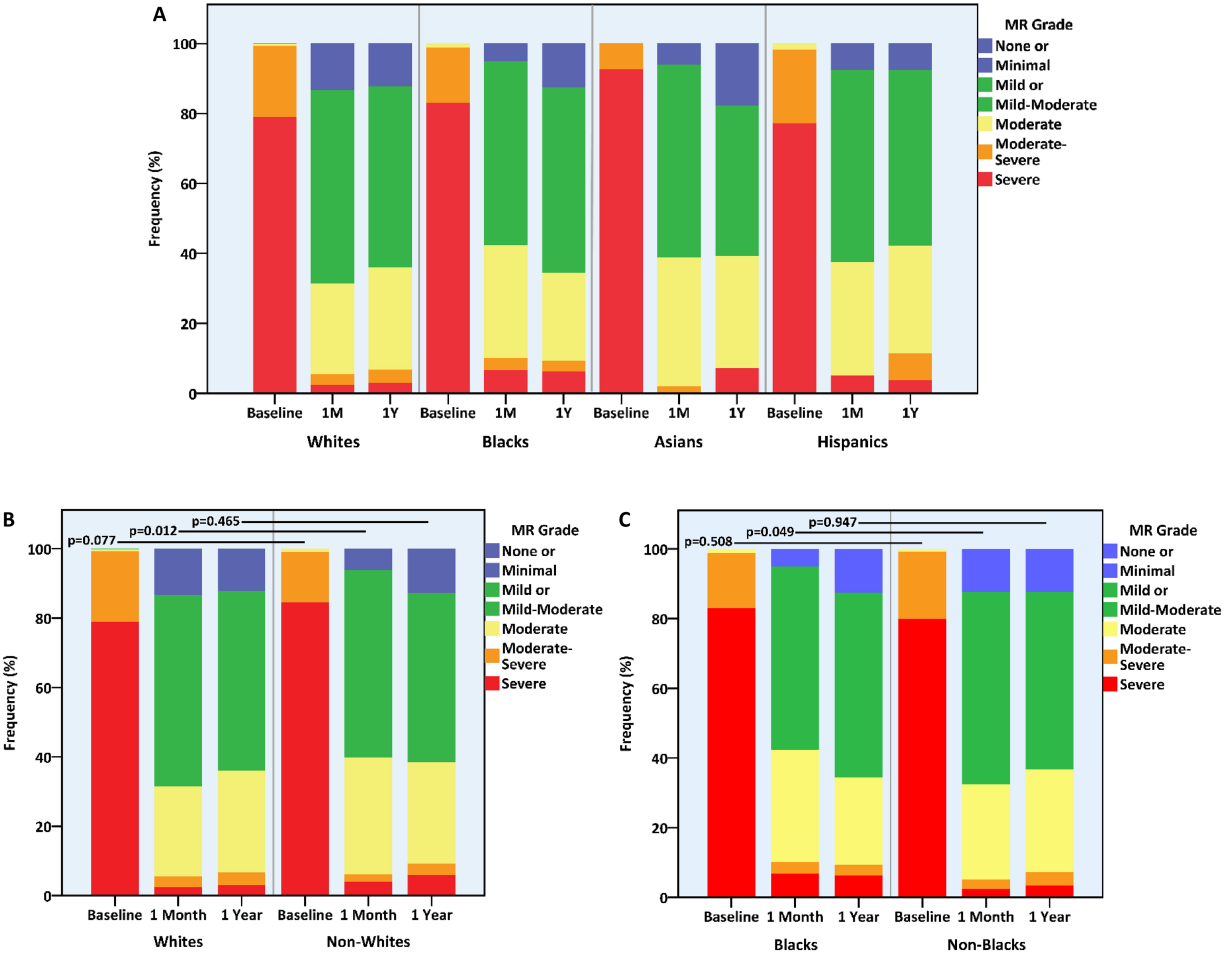
Mitral regurgitation grade at baseline and following mitral transcatheter edge-to-edge repair according to race. **(A)** All races, **(B)** whites vs. non-whites, **(C)** blacks vs. non-blacks. MR, mitral regurgitation.

MR of up to mild or up to moderate degree was equally maintained in the four racial groups at the 1-month and 1-year marks (Table 3). Nevertheless, 1-month MR was generally more severe in non-whites vs. whites (*p* = 0.012) and in blacks vs. non-blacks (*p* = 0.049) (Figure 3). Concurrently, 1-year LVMi proved lower in whites vs. non-whites (110.0 (IQR, 90.1–133.9) vs. 125.8 (IQR, 103.6–152.0) gr/m^2^, *p* = 0.001) and higher in Hispanics vs. non-Hispanics (140.5 (IQR, 116.4–165.8) vs. 111.0 (IQR, 91.1–137.1) gr/m^2^, *p* = 0.001) (Table 3). The relative reduction (i.e., improvement) in LVMi from baseline and over the span of a year, however, was unaffected by race.

Overall, whites (*n* = 324, 90%) and non-blacks (*n* = 377, 90.0%) enjoyed higher rates of freedom from either >mild MR or a NYHA class III-IV by 1-year compared to blacks (*n* = 29, 78.4%) (*p* = 0.049 for both comparisons) (Table 3).

### Subgroup analysis for functional and primary mitral regurgitation

Most race-related differences in baseline and procedural features, as observed in the entire cohort, were evident within the functional MR subgroup, which included 494 (51.2%) of the study patients (Supplementary Tables 3–5). However, RV dysfunction at baseline, hemodynamic status at presentation, and MR severity at hospital discharge were similar in the four racial groups. Hospitalization was still the lengthiest in blacks, but not statistically significant.

As in the total cohort, non-whites (vs. whites) and blacks (vs. non-blacks or whites) experienced higher cumulative incidence of the primary outcome, which again were accounted for by HF hospitalization events (Supplementary Table 6 and Supplementary Figures 3–5). Also, blacks faced less improvement in NYHA functional class (Supplementary Table 6 and Supplementary Figure 6). Other than a lower LVMi in whites vs. non-whites, no differences were noted in echocardiographic measures at 1 year (Supplementary Table 6 and Supplementary Figure 7). The combined 1-year rates of NYHA class I-II or MR of up to mild degree were non-significantly highest among whites and Asians and lowest in blacks and Hispanics.

Within the primary MR sub-cohort, there were fewer differences in baseline characteristics between the races (Supplementary Tables 7, 8). Still, whites were the oldest, most fully insured, highest-paid, most educated, and least diabetic. Also, they were less likely to present with acute HF or hemodynamic instability. Blacks had the lowest LVEF and highest PASP values, received more clips per procedure, and experienced the lowest rate of up to mild MR at discharge (Supplementary Table 9).

Overall, 1-year clinical and echocardiographic outcomes, as well as periprocedural adverse events, were non-different across different racial groups with primary MR (Supplementary Table 10 and Supplementary Figures 8–12). Notably, the primary MR subgroup as a whole exhibited significantly fewer primary outcome events compared to the functional MR subgroup (78/470, 16.6% vs. 161/494, 32.6%, *p* < 0.001). While 1-month MR was generally less severe in whites (vs. non-whites) and in non-blacks (vs. blacks), up to moderate MR was equally achieved.

A Cox regression analysis performed separately in each of the two main MR etiologic subgroups identified white race as an independent protective factor and black race as an independent risk factor for the primary outcome – but only in patients with functional MR (HR for white race 0.62, 95% CI 0.39–0.99, *p* = 0.047; HR for black race 2.07, 95% CI 1.28–3.35, *p* = 0.003) (Supplementary Table 2 and Table 4). Conversely, no race was associated with the risk for the combined endpoint of death or HF hospitalizations among primary MR patients.

### Propensity score matching

Within the 2 matched cohorts – one with 147 patients of either white or non-white race and one with 65 patients of either black or non-black race – most inter-racial differences in baseline characteristics and periprocedural aspects vanished (Supplementary Tables 11–14). Yet, non-whites (vs. whites) and blacks (vs. non-blacks) again experienced higher cumulative incidence of all-cause mortality, HF hospitalizations, and the composite of both, reaching statistical significance for HF hospitalizations and in blacks also for the primary outcome (Supplementary Table 14). Moreover, these two subgroups exhibited numerically lower rates of non-significant MR following TEER. After multivariable analysis, black race remained associated with a higher risk for the primary outcome in the blacks vs. non-blacks matched cohort (HR 1.73, 95% CI 1.02–3.33, *p* = 0.044), while in the whites vs. non-whites matched cohort its presence imposed a trend toward worse outcome (HR 1.84, 95% CI 0.99–3.42, *p* = 0.055); Non-white race did not demonstrate an independent predictive significance anymore (Supplementary Table 15).

## Discussion

**Figure 4:**
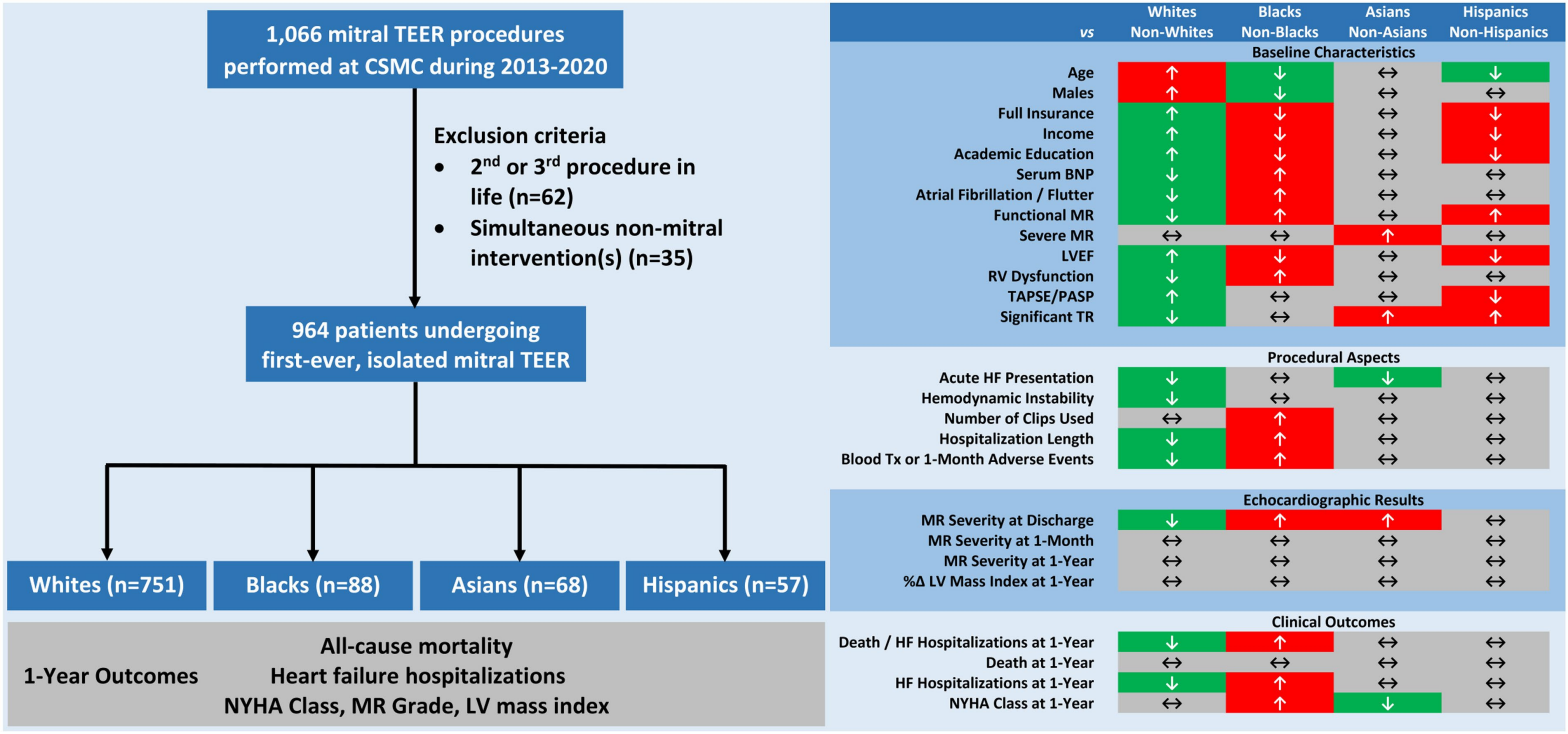
CENTRAL ILLUSTRATION Racial disparities in characteristics and outcomes of patients undergoing mitral transcatheter edge-to-edge repair. Among 964 patients undergoing isolated, first-time mitral transcatheter edge-to-edge repair, major inter-racial differences were note in pre-, intra-, and post-procedural features. At 1-year, non-whites and blacks experienced higher rates of all-cause mortality or heart failure hospitalizations and blacks also faced less improvement in functional status. Mitral regurgitation severity and left ventricular hypertrophy equally regressed in all groups. BNP, B-type natriuretic peptide; CSMC, Cedars-Sinai Medical Center; EF, ejection fraction; HF, heart failure; LV, left ventricular; MR, mitral regurgitation; NYHA, New York Heart Association; PASP, pulmonary arterial systolic pressure; RV, right ventricular; TAPSE, tricuspid annular plane systolic excursion; TR, tricuspid regurgitation.

Our study evaluated the characteristics and 1-year outcomes of patients referred to mitral TEER according to their race. Based on a large, contemporary, real-world registry, we made the following observations (Figure 4, CENTRAL ILLUSTRATION): (1) Non-whites were under-represented in the cohort; (2) Pre-, intra-, and post-procedural features, including demographics, comorbidities, baseline echocardiographic parameters, hemodynamic status at presentation, number of deployed clips, hospitalization length, and 1-month course differed substantially between races; (3) Non-whites (vs. whites) and blacks (vs. non-blacks or whites) experienced similar death rates but earlier, more frequent HF hospitalizations during the first postprocedural year; (4) One-year functional status was worse in blacks (vs. non-blacks) and in non-Asians (vs. Asians), however MR severity and reverse LV remodeling by TTE were comparable across races; (5) Non-white and black races independently imposed a higher risk for the combined outcome of 1-year all-cause mortality or HF hospitalizations; and (6) The prognostic implication and predictive ability of racial background were confined to the functional MR sub-cohort.

To the best of our knowledge, this is the first study to report on mitral TEER outcomes in various racial groups beyond the index hospitalization. Furthermore, it is among the first to explicitly consider in this context all four major races residing in the US, particularly Hispanics, who are increasingly being recognized as a distinct racial/ethnic group (18, 19). While previous studies (7–9) relied on an administrative, billing-focused database – the National Inpatient Sample (NIS) – we used a clinical, patient-level registry that was prospectively constructed by physicians, thus reinforcing clinical relevance which allowed for a pioneering MR etiology-based subgroup analysis. Also, our cohort consisted of patients undergoing an isolated mitral TEER for the first time and in one center, thus eliminating possible interactions with non-mitral procedures and with institution and operator-related factors, all of which may have been presented in the earlier nationwide works.

As previously shown in both mitral TEER (8, 9) and non-mitral TEER populations (5, 6), our findings demonstrated a discrepancy between racial segmentation of the general public and that of an interventional cardiology cohort, which further expanded as patients aged. While non-white individuals comprised 22.1% of patients who underwent mitral TEER at our institution between 2013 and 2020, their percentage among those aged ≥65 or ≥ 75 years was 18.7% or 15.3%, respectively. At the same time, the national percentage of non-whites steadily rose from 36.3% in 2010 to 42.2% in 2020 (19). Moreover, within the senior US population, non-whites constituted 22.8 and 24.0% of ≥65 and ≥ 75-year-old individuals in 2016, respectively (20), and by 2019, as high as 24% of elderly Americans were non-white (21). Compared to the State of California, in which (non-Hispanic) whites are a minority (19), our registry probably under-represented non-whites even further. As the prevalence of ≥moderate MR has been shown to be comparable between races (22), the disparities in mitral TEER utilization in the present study could have reflected gaps in access to medical care, and specifically high-volume centers as ours (23). Consistent with this assumption were the significant inter-racial differences in insurance coverage, as well as income and level of academic education, making whites the highest-paid, most educated and fully-insured group, and blacks and Hispanics – the least ones. Such differences, too, have been previously observed (24).

Further consistent with published data (6–9, 22, 25), blacks treated at our institution were relatively younger and more likely to be women compared to patients of other racial origins. As in those previous reports, they had an overall higher burden of comorbidities and biventricular dysfunction and suffered more commonly from functional MR. The earlier presentation, worse medical condition, and increased prevalence of functional MR within the black group possibly mirrored and accounted for one another. Considering the female predominance observed in blacks undergoing TEER, they could also have been brought about by prior peripartum cardiomyopathy, which is known to primarily affect young black women, and which may transform later in life to a chronic HF condition. Although not directly explored in our registry, and not clearly associated with outcomes according to a Cox regression model, non-ischemic cardiomyopathy did prove more common among blacks, and particularly in black females with functional MR (28/42, 66.7% vs. 75/90, 45.5%, *p* = 0.014). Apart from representing genuine medical interactions, the higher disease burden experienced by blacks may have implied a delay in diagnosis and treatment, which could again reflect inequality in access to medical care. While reasonable, this last notion remained hypothetical as our registry did not include information about the timing of mitral TEER in relation to the emergence of overt indications for intervention.

Adding to present-day evidence of a higher incidence of in-hospital complications (9) in non-whites and lower odds of next-day discharge (10) in black patients following mitral TEER, our study demonstrated a more complex course affecting these racial groups up to 1 year after the procedure, as well. This was likely the result of the worse health status, the more advanced HF and LV dysfunction, the increased prevalence of functional MR, and the lower socioeconomic indices that characterized non-whites and blacks prior to the procedure. In blacks, the less favorable outcome also could have represented the increased number of clips used and a possible attenuated effect of RAS inhibitors (26–28). Indeed, our comprehensive multivariable analysis suggested several comorbidities, as well as LV enlargement, functional MR, higher number of deployed clips, and no use of RAS inhibitors as independent risk factors for the 1-year composite endpoint of death or HF hospitalizations, similar to earlier works (17, 29–31). Yet, blacks received more clips regardless of MR etiology, were similarly hypertensive as non-blacks, and utilized comparable medical prescriptions in the periprocedural period. Furthermore, non-white and black races remained prognostically meaningful after adjusting for inter-racial differing parameters, all implying the presence of additional race-related factors not measured in our study that could have determined the post-TEER course. Some of these variables, such as more distant post-interventional management and adherence to treatment, also could have interacted with socioeconomic parameters discussed earlier. Importantly, MR severity itself did not play a role in this regard, as it was comparable across races and did not possess any association with the primary outcome.

Interestingly, racial disparities in patient outcomes were mainly observed between whites and non-whites and between blacks and non-blacks. Also, they were limited to the functional MR sub-cohort, in which non-white and black races independently conferred a higher risk for the primary outcome. One explanation for these findings may lie in statistical power, as racial groups other than whites and blacks were relatively small. Similarly, although comparable in size, the primary MR subgroup experienced fewer composite events of death or hospitalizations, again signifying a lower power that could have masked inter-racial differences. Besides statistical considerations, baseline and procedural characteristics, and specifically age, sex, and insurance, were less varied among patients of different races who had primary MR, potentially contributing to a more balanced and homogenous downstream course. The reason underlying the association between race and demographics specifically within the functional MR sub-cohort was unclear, and may again relate to the possibly different prevalence of non-ischemic cardiomyopathy.

## Limitations

First, our study represents a retrospective analysis from one center which did not employ an external core laboratory. Second, missing data regarding functional status and echocardiographic measures over the course of follow-up could have interfered with the interpretation of the results. However, these were similarly distributed among the various racial groups, making survival bias unlikely. Moreover, baseline characteristics and procedural details, as well as deaths and hospitalizations, were all documented for the entire cohort. Third, our matched cohorts were relatively small and may not fully control for all unmeasured confounders. Therefore, all multivariable analyses based on these matched populations should be considered exploratory. Fourth, although consistent with a real-world setting (32), medical therapy was suboptimal in present-day standards, making it difficult to extrapolate our observations to medically optimized patients. Fifth, our registry did not include direct information on socioeconomic variables other than insurance, neither did it include data on specific causes of death, thus preventing the appreciation of possible interactions with race. Sixth, we assigned only one race to each patient and based this categorization on self-reporting, not taking into consideration the possibility of mixed racial backgrounds. Lastly, our results may not apply to non-MitraClip systems as those were not employed in our center.

## Conclusion

In our single-center experience, major differences in pre-, intra-, and post-procedural features were observed between various racial groups undergoing mitral TEER. The 1-year composite outcome of all-cause mortality or HF hospitalizations occurred more frequently among non-whites (vs. whites) and blacks (vs. non-blacks or whites) and was independently associated with non-white and black races. Functional status improved to a lesser extent in blacks. The prognostic implication of race was restricted to the functional MR subgroup.

## Data availability statement

The raw data supporting the conclusions of this article will be made available by the authors, without undue reservation.

## Ethics statement

The studies involving human participants were reviewed and approved by the Cedars-Sinai Medical Center Institutional Review Board. Written informed consent for participation was not required for this study in accordance with the national legislation and the institutional requirements.

## Author contributions

AS has conceptualized the project, gathered data, performed analyses, and written the first draft of the manuscript. Other co-authors have assisted in revision of the text, as well as in the formulation of the statistical methodology. All authors contributed to the article and approved the submitted version.

## Funding

This study was supported in part by the California Chapter of the American College of Cardiology through the Save a Heart foundation.

## Conflict of interest

RM received grant support from Edwards Lifesciences Corporation, is a consultant for Abbott Vascular, Cordis, and Medtronic, and holds equity in Entourage Medical. TC is a consultant, proctor, and speaker for Edwards Lifesciences and Medtronic, is a consultant for Abbott Lifesciences, and is a consultant and speaker for Boston Scientific.

The remaining authors declare that the research was conducted in the absence of any commercial or financial relationships that could be construed as a potential conflict of interest.

## Publisher’s note

All claims expressed in this article are solely those of the authors and do not necessarily represent those of their affiliated organizations, or those of the publisher, the editors and the reviewers. Any product that may be evaluated in this article, or claim that may be made by its manufacturer, is not guaranteed or endorsed by the publisher.

## Abbreviations

BNP: B-type natriuretic peptide
HF: Heart failure
KCCQ: Kansas City Cardiomyopathy Questionnaire
LVEF: Left ventricular ejection fraction
MR: Mitral regurgitation
NYHA: New York Heart Association
TEER: Transcatheter edge-to-edge repair.
