# Supplementary material for: Racial disparities in characteristics and outcomes of patients undergoing mitral transcatheter edge-to-edge repair
Source: Front Cardiovasc Med. 2023 Mar 2;10:1111714. doi: 10.3389/fcvm.2023.1111714 (PMC10018123; doi:10.3389/fcvm.2023.1111714)
Supplement: Supplementary file 1 [file Data_Sheet_1.pdf]

Table of Contents

Tables

Supplemental Table 1 2

Supplemental Table 2 11

Supplemental Table 3 14

Supplemental Table 4 17

Supplemental Table 5 19

Supplemental Table 6 22

Supplemental Table 7 41

Supplemental Table 8 44

Supplemental Table 9 46

Supplemental Table 10 49

Supplemental Table 11 69

Supplemental Table 12 71

Supplemental Table 13 73

Supplemental Table 14 75

Supplemental Table 15 77

Figures

Supplemental Figure 1 5

Supplemental Figure 2 8

Supplemental Figure 3 24

Supplemental Figure 4 29

Supplemental Figure 5 32

Supplemental Figure 6 35

Supplemental Figure 7 38

Supplemental Figure 8 51

Supplemental Figure 9 56

Supplemental Figure 10 59

Supplemental Figure 11 62

Supplemental Figure 12 65

Supplemental Table 1. Procedural Details and Results Observed in the Total Cohort According to Race

	Whites (N=751)	Blacks (N=88)	Asians (N=68)	Hispanics (N=57)	P-Value				
					**Whites vs Non-Whites**	**Blacks vs Non-Blacks**	**Asians vs Non-Asians**	**Hispanics vs Non-Hispanics**	**Whites vs Blacks**
**Presentation to Procedure**									
Acute Decompensated Heart Failure	106 (14.1)	19 (21.6)	10 (14.7)	16 (28.1)	**0.013**	0.109	0.822	**0.008**	0.062
Cardiogenic Shock	22 (2.9)	3 (3.4)	3 (4.4)	1 (1.8)	0.788	0.742	0.452	0.568	0.739
Medical and/or Mechanical Hemodynamic Support	38 (5.1)	8 (9.1)	8 (11.8)	4 (7.0)	**0.019**	0.203	0.057	0.771	0.133
Urgent Procedure	132 (17.6)	21 (23.9)	17 (25.0)	7 (12.3)	0.238	0.162	0.142	0.222	0.148
**Procedural Aspects**									
Number of Clips Deployed									
0 (Aborted / Not Deployed)	20 (2.7)	1 (1.1)	1 (1.5)	0 (0.0)	0.193	0.713	1.000	0.635	0.715
1	321 (42.7)	22 (25.0)	32 (47.1)	30 (52.6)	0.388	**0.001**	0.382	0.094	**0.001**
2	306 (40.7)	45 (51.1)	30 (44.1)	22 (38.6)	0.211	0.063	0.688	0.613	0.062
≥2	410 (54.6)	65 (73.9)	35 (51.5)	27 (47.6)	0.192	**<0.001**	0.466	0.191	**0.001**
≥3	104 (13.8)	20 (22.7)	5 (7.4)	5 (8.8)	0.930	**0.012**	0.105	0.249	**0.026**
Median	2 (1-2)	2 (1-2)	2 (1-2)	1 (1-2)	0.223	**0.001**	0.265	0.203	**<0.001**
Device Generation									
1	239 (31.8)	22 (25.0)	19 (27.9)	25 (43.9)	0.816	0.160	0.496	**0.041**	0.191
2	242 (32.2)	28 (31.8)	23 (33.8)	24 (42.1)	0.413	0.823	0.864	0.127	0.939
3	191 (25.4)	25 (28.4)	15 (22.1)	2 (3.5)	0.086	0.330	0.673	**<0.001**	0.546
4	79 (10.5)	13 (14.8)	11 (16.2)	6 (10.5)	0.147	0.282	0.188	0.848	0.227
Intervention Site									

A1P1	23 (3.1)	0 (0.0)	4 (5.9)	0 (0.0)	0.355	0.164	0.116	0.399	0.159
A2P2	716 (95.3)	86 (97.7)	61 (89.7)	57 (100.0)	0.788	0.421	**0.030**	0.104	0.416
A3P3	46 (6.1)	4 (4.5)	7 (10.3)	1 (1.8)	0.790	0.543	0.178	0.248	0.811
Total Duration (min)	110 (90-138)	119 (88-133)	106 (84-142)	115 (87-138)	0.775	0.308	0.459	0.949	0.355
Fluoroscopy Duration (min)	19 (14-26)	20 (15-31)	19 (13-27)	17 (13-24)	0.573	0.195	0.817	0.399	0.212
Conversion to Surgery	1 (0.1)	0 (0.0)	0 (0.0)	0 (0.0)	1.000	1.000	1.000	1.000	1.000
**Echocardiographic and Hemodynamic Effects**									
Mitral Regurgitation Severity Reduction to ≤Mild									
Immediately after Clip Deployment	538 (71.6)	59 (67.0)	48 (70.6)	43 (75.4)	0.724	0.347	0.883	0.484	0.368
At Discharge	594 (81.4)	61 (69.3)	45 (69.2)	42 (75.0)	**0.001**	**0.019**	**0.045**	0.446	**0.008**
Transmitral Mean Pressure Gradient (mmHg)									
Immediately after Clip Deployment	3 (2-4)	3 (2-4)	3 (2-4)	3 (2-5)	0.951	0.686	0.735	0.454	0.707
At 1-Month	4 (3-5)	5 (3-6)	3 (3-5)	4 (3-6)	0.713	**0.036**	0.112	0.889	0.050
Pulmonary Venous Flow Pattern Normalization on ≥1 Side	424 (63.4)	52 (67.5)	38 (66.7)	28 (59.6)	0.652	0.471	0.637	0.539	0.473
Delta V wave (mmHg)	-7 (-18-[-1])	-13 (-30-[-4])	-13 (-27-0)	-12 (-27-0)	**<0.001**	**0.001**	0.054	0.512	**<0.001**
Delta Mean Left Atrial Pressure (LAP) (mmHg)	-3 (-8-1)	-5 (-12-[-1])	-6 (-13-1)	-5 (-11-0)	**<0.001**	**0.003**	**0.041**	0.144	**0.001**
Delta Mean Pulmonary Arterial Pressure (PAP) (mmHg)	0 (-6-3)	-1 (-8-4)	-1 (-11-4)	-3 (-8-3)	0.171	0.721	0.322	0.382	0.603
**Post-Procedural Course**									
Intensive Cardiac Unit (ICU) Stay Duration (hours)	16.5±98.7	42.0±167.6	21.8±79.4	8.2±22.9	0.266	0.163	0.809	0.421	0.167
Hospitalization Length (days)	1 (1-4)	3 (1-9)	1 (1-5)	2 (1-8)	**0.001**	**0.001**	0.991	0.050	**0.001**
Discharge Home	688 (93.4)	81 (94.2)	65 (98.5)	51 (91.1)	0.477	0.831	0.116	0.393	0.767
Blood Transfusion or Any 1-Month Non-Fatal Adverse Event*	80 (10.7)	17 (19.3)	12 (17.6)	8 (14.0)	**0.008**	**0.030**	0.149	0.651	**0.016**
**Medical Treatment at 1-Month**									
Beta Blockers	416 (69.2)	52 (71.2)	33 (61.1)	33 (70.2)	0.817	0.854	0.427	0.948	0.881

Renin Angiotensin System (RAS) Inhibitors	311 (52.2)	40 (55.6)	28 (52.8)	21 (44.7)	0.919	0.536	0.910	0.294	0.588
Mineralocorticoid Receptor Antagonists (MRAs)	112 (18.6)	23 (31.5)	10 (18.5)	13 (27.1)	**0.027**	**0.013**	0.727	0.232	**0.009**
Loop Diuretics	419 (69.7)	60 (82.2)	39 (72.2)	34 (70.8)	0.106	**0.028**	0.855	0.962	**0.026**
Anti-Arrhythmics	130 (21.8)	19 (26.4)	10 (18.9)	13 (27.1)	0.661	0.639	0.799	0.683	0.619
Hydralazine + Nitrates	11 (1.8)	6 (8.1)	1 (1.8)	2 (4.2)	**0.027**	**0.008**	0.706	0.349	**0.006**
Oral Anticoagulants	307 (51.1)	28 (38.4)	23 (42.6)	17 (35.4)	**0.004**	0.073	0.382	0.065	**0.040**

Data are presented as number (percentage), median (interquartile range), or mean±standard deviation, where appropriate.

* 1-month non-fatal adverse events included the following: tamponade, cardiac arrest, myocardial infarction, stroke, transient ischemic attack, MVARC bleeding, and vascular complications.

MVARC = mitral valve academy research consortium

Supplemental Figure 1. One-Year Cumulative Incidence of Heart Failure Hospitalizations Following Mitral Transcatheter Edge-to-Edge Repair According to Race

A All Races

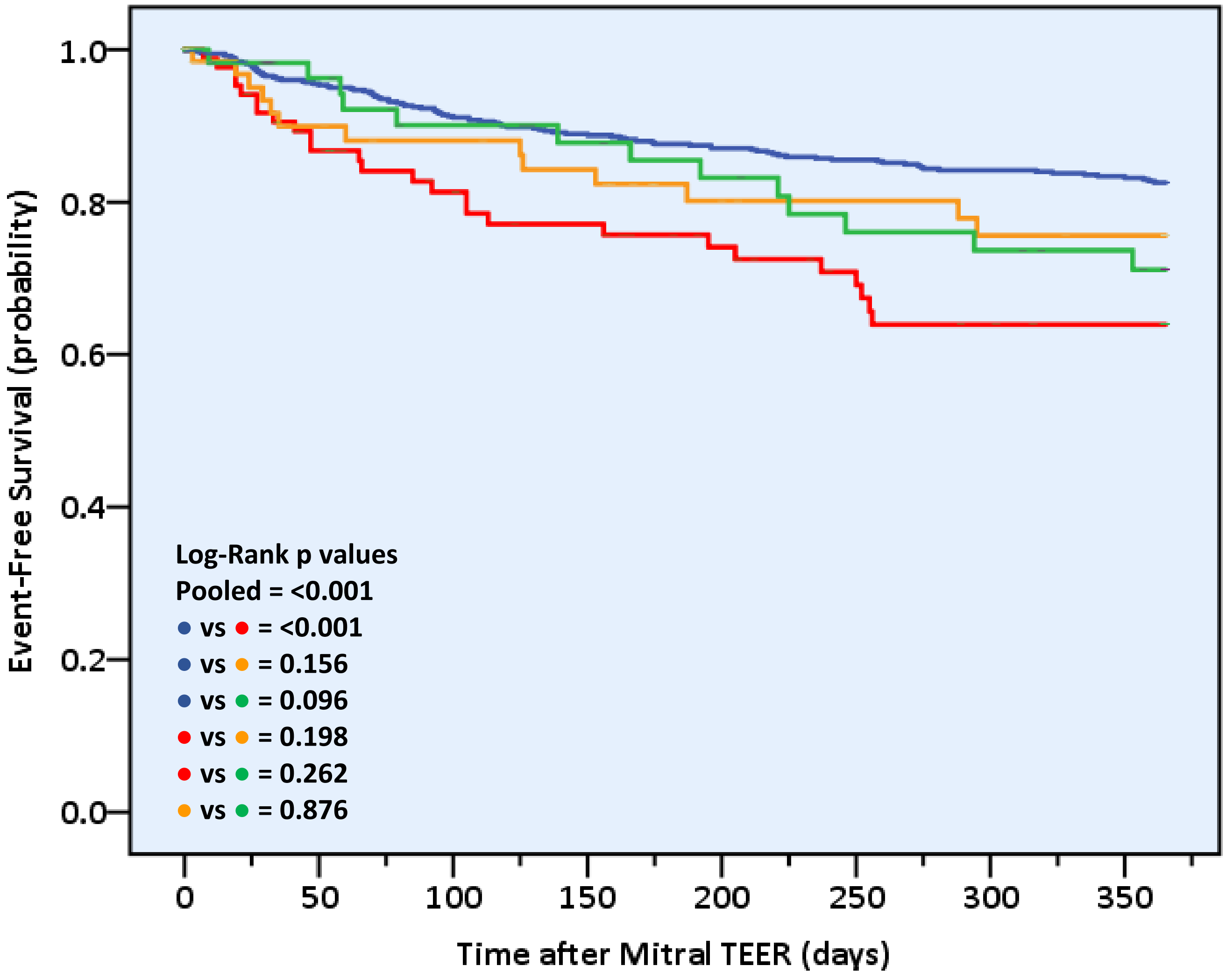

**No. at Risk**								
**Whites**	751	577	517	488	460	439	425	404
**Blacks**	88	69	59	54	47	40	36	36
**Asians**	68	50	47	44	36	35	33	32
**Hispanics**	57	48	42	39	36	32	31	29

B Whites vs non-Whites

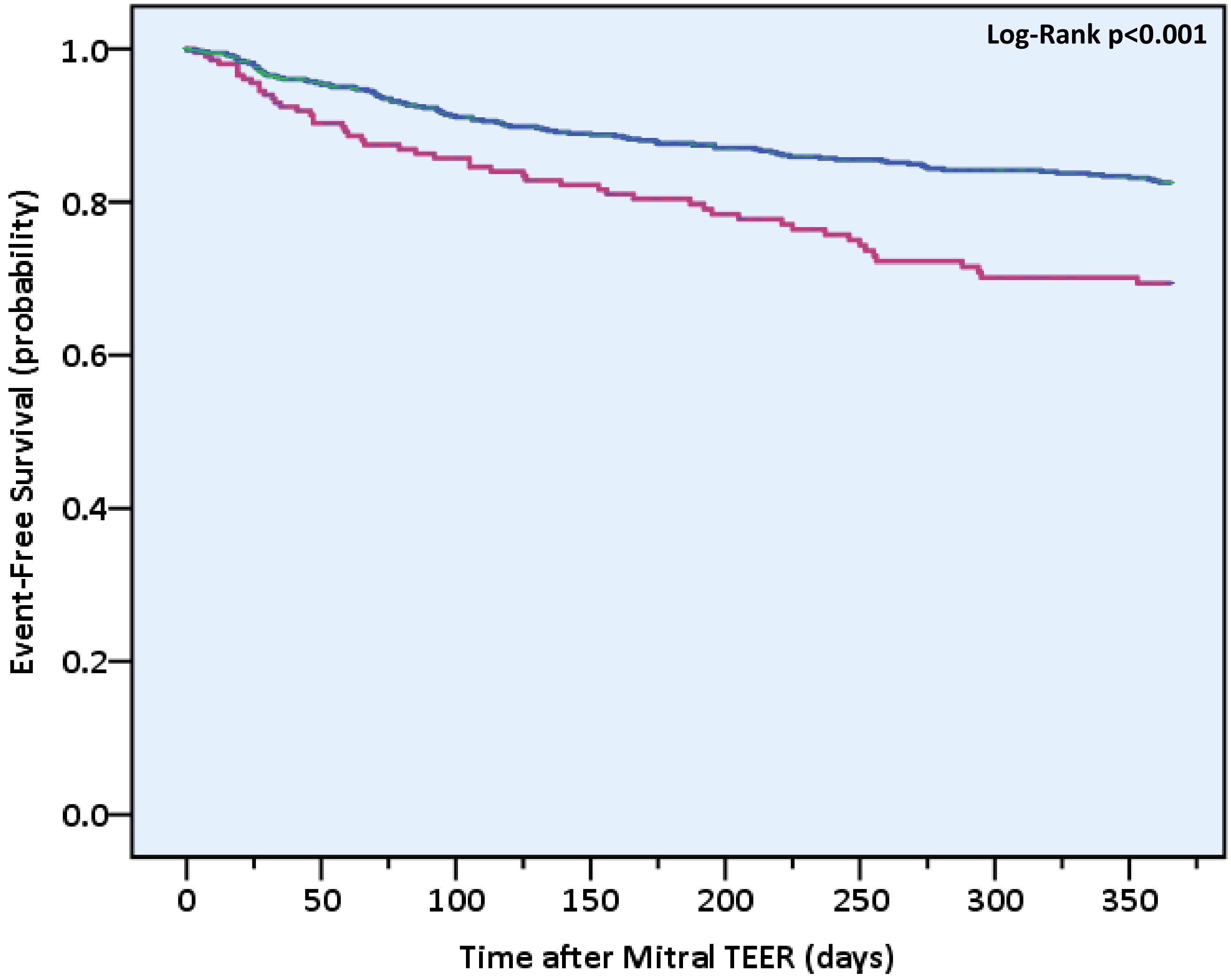

**No. at Risk**								
Whites	751	577	517	488	460	439	425	404
Non-Whites	213	167	148	137	119	107	100	95

C Blacks vs non-Blacks

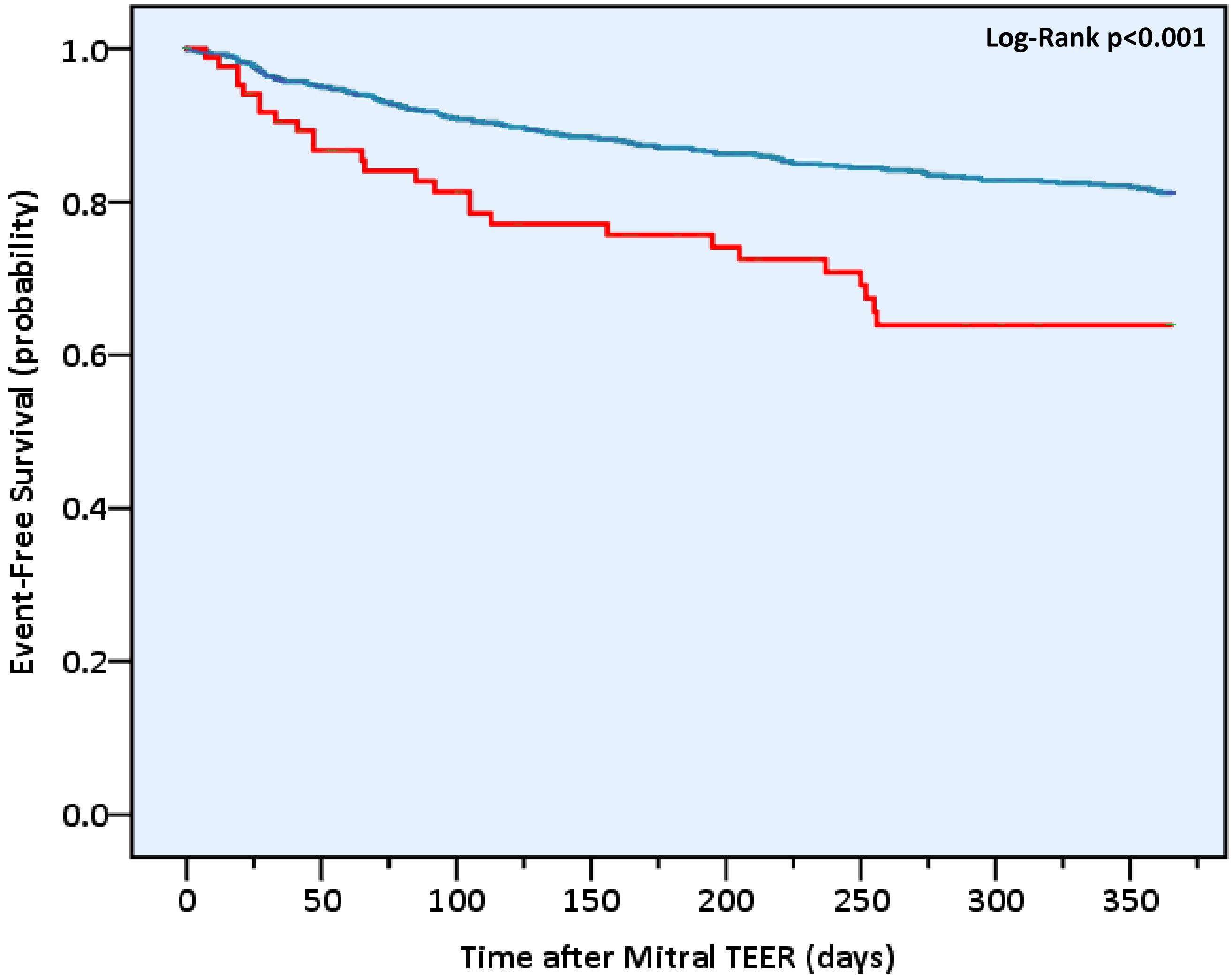

**No. at Risk**								
**Non-Blacks**	876	675	606	571	532	506	489	465
**Blacks**	88	69	59	54	47	40	36	36

TEER = transcatheter edge-to-edge repair

Supplemental Figure 2. One-Year Cumulative Incidence of All-Cause Mortality Following Mitral Transcatheter Edge-to-Edge Repair According to Race

A All Races

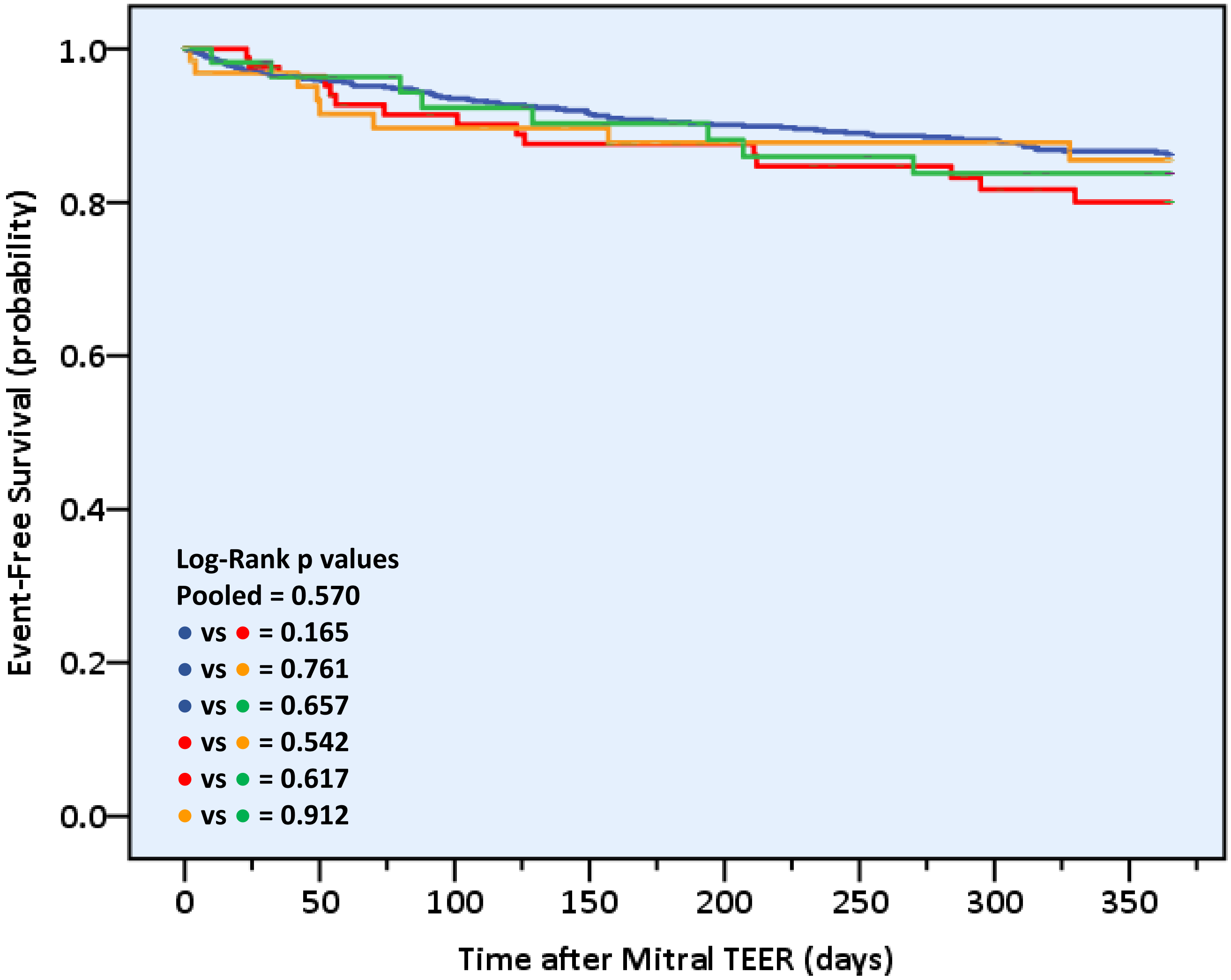

No. at Risk								
Whites	751	603	563	536	514	495	484	465
Blacks	88	78	71	68	62	57	53	50
Asians	68	51	49	47	41	40	39	38
Hispanics	57	50	46	44	41	40	38	36

B Whites vs non-Whites

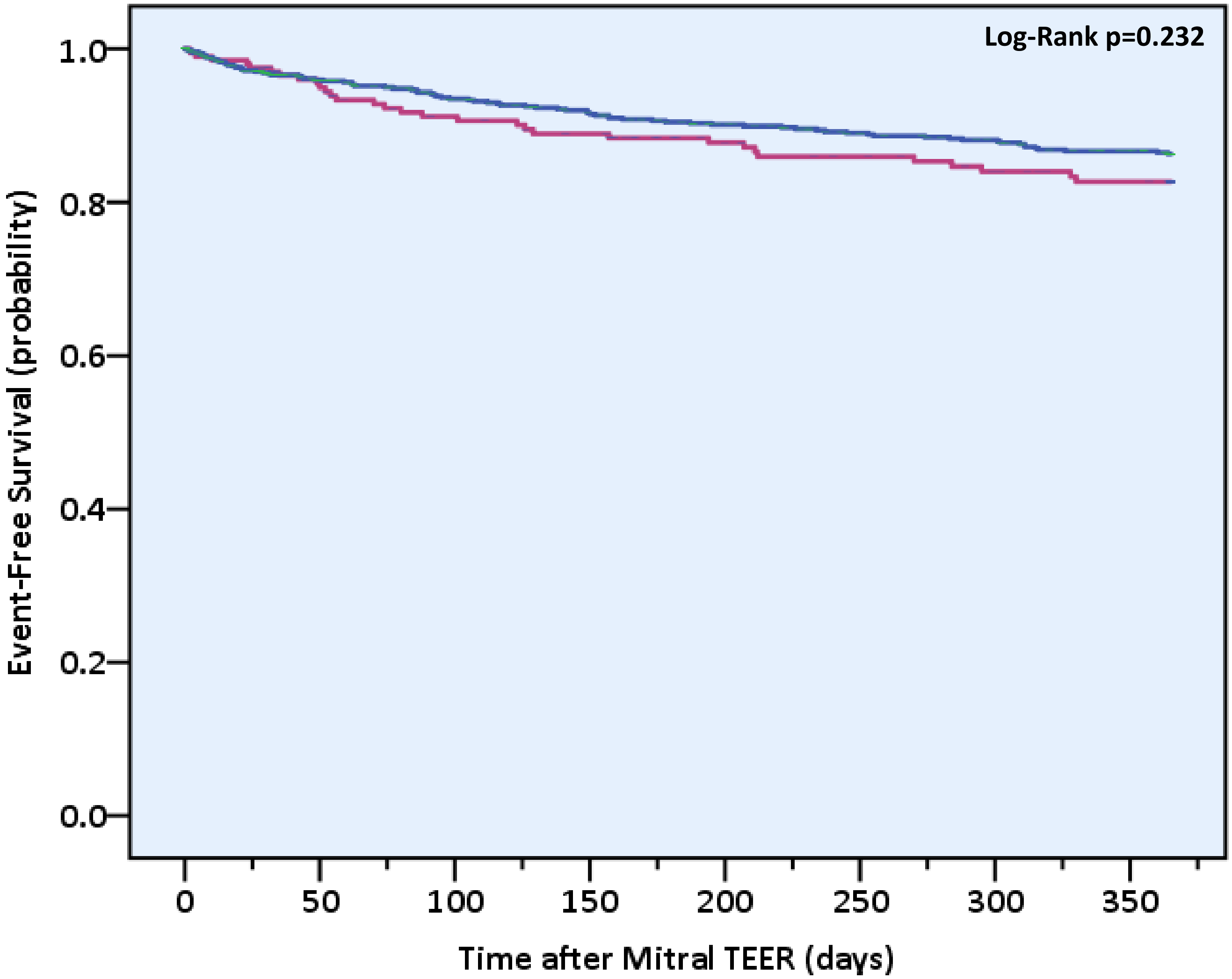

**No. at Risk**								
**Whites**	751	603	563	536	514	495	484	465
**Non-Whites**	213	179	166	159	144	137	130	124

C Blacks vs non-Blacks

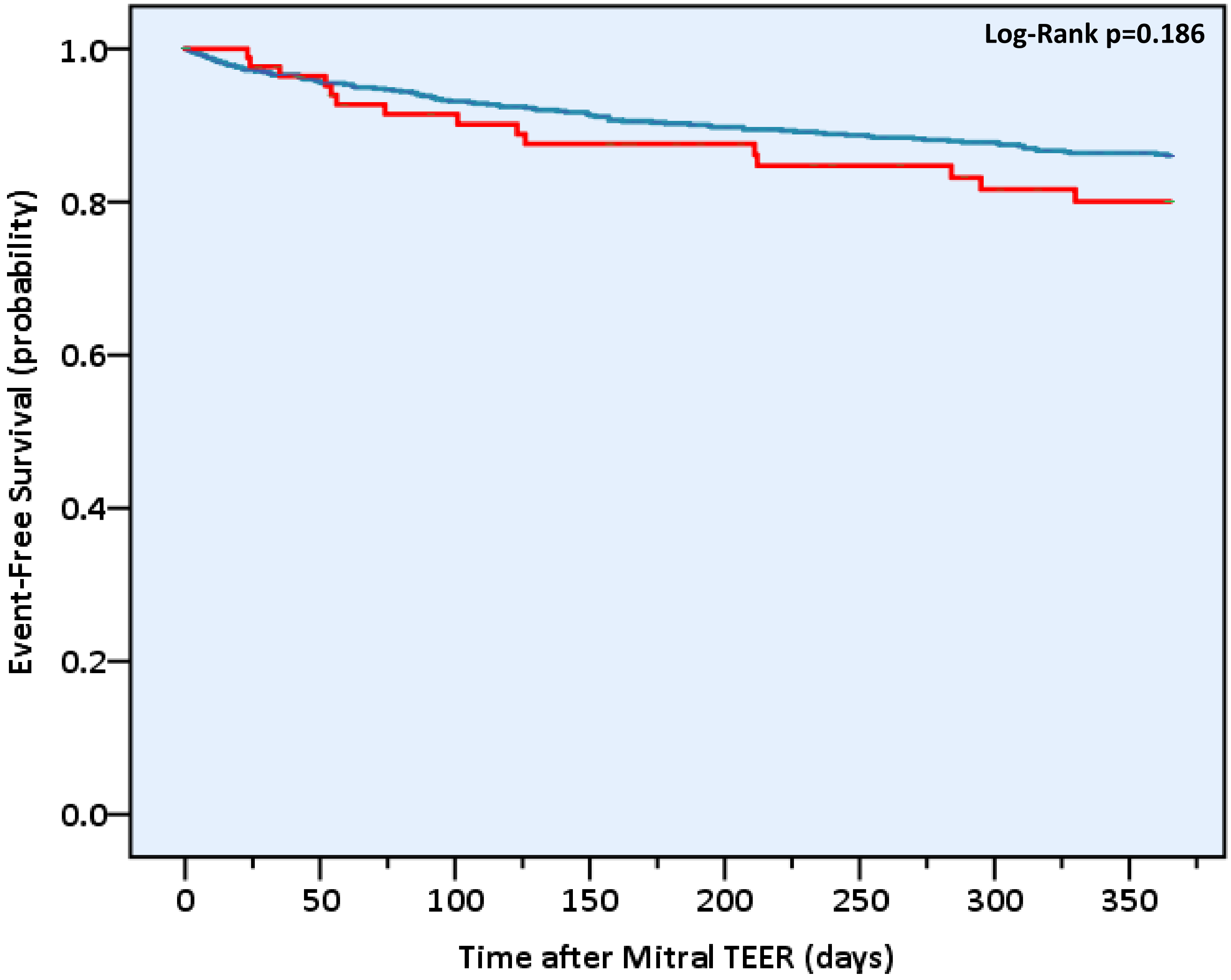

No. at Risk								
Non-Blacks	876	704	658	627	596	575	561	539
Blacks	88	78	71	68	62	57	53	50

TEER = transcatheter edge-to-edge repair

Supplemental Table 2. Univariable Cox Proportional Hazard Model for the Combined Outcome of All-Cause Mortality or Heart Failure Hospitalizations at 1 Year Following Mitral Transcatheter Edge-to-Edge Repair

	Total Cohort		Functional MR		Primary MR	
	**HR (95% CI)**	**P-Value**	**HR (95% CI)**	**P-Value**	**HR (95% CI)**	**P-Value**
**Baseline Clinical Variables**						
Age						
Continuous	0.99 (0.98-0.99)	0.038	1.00 (0.99-1.01)	0.546	1.00 (0.98-1.02)	0.742
≥75 years	0.84 (0.65-1.08)	0.175	0.96 (0.70-1.30)	0.771	1.20 (0.71-2.03)	0.497
Sex Male	0.94 (0.72-1.21)	0.608	0.94 (0.69-1.29)	0.705	1.03 (0.65-1.63)	0.997
Race						
White vs non-White	0.66 (0.50-0.87)	0.003	0.72 (0.52-0.99)	0.041	0.94 (0.51-1.73)	0.833
Black vs non-Black	1.77 (1.23-2.54)	0.002	1.51 (1.02-2.23)	0.040	1.05 (0.33-3.33)	0.934
Asian vs non-Asian	1.14 (0.71-1.84)	0.591	0.98 (0.52-1.89)	0.959	1.50 (0.72-3.13)	0.274
Hispanic vs non-Hispanic	1.23 (0.76-1.98)	0.407	1.23 (0.74-2.04)	0.418	0.33 (0.05-2.36)	0.268
Insurance						
None vs Low-Income or Regular / Full	1.96 (0.49-7.89)	0.344	2.22 (0.55-8.96)	0.265	2.08 (0.86-2.23)	0.774
Low-Income vs Regular / Full	1.57 (1.13-2.18)	0.008	1.40 (0.97-2.03)	0.076	1.18 (0.55-2.58)	0.665
None or Low-Income vs Regular / Full	1.59 (1.15-2.20)	0.005	1.44 (1.02-2.08)	0.048	1.14 (0.53-2.48)	0.738
Median Yearly Household Income* (continuous)	0.97 (0.96-0.99)	0.019	0.99 (0.98-1.01)	0.449	1.00 (0.99-1.01)	0.102
Percentage of Adults with Academic Degree* (continuous)	0.99 (0.98-1.01)	0.087	1.00 (0.99-1.01)	0.635	0.99 (0.98-1.02)	0.092
Obesity	0.99 (0.71-1.38)	0.939	1.07 (0.74-1.56)	0.722	0.60 (0.26-1.22)	0.143
Diabetes Mellitus	1.59 (1.22-2.09)	0.001	1.43 (1.04-1.96)	0.030	1.46 (0.87-2.46)	0.152
Hypertension	1.08 (0.76-1.52)	0.676	0.98 (0.64-1.49)	0.914	1.16 (0.64-2.10)	0.633
Smoking History	1.31 (0.71-2.40)	0.384	1.46 (0.77-2.77)	0.246	0.45 (0.06-3.24)	0.429
Previous MI, PCI, or CABG	1.42 (1.10-1.83)	0.007	1.07 (0.79-1.46)	0.665	1.55 (0.99-2.43)	0.057
Prior Stroke or Transient Ischemic Attack (TIA)	1.29 (0.91-1.83)	0.147	1.17 (0.78-1.77)	0.451	1.33 (0.70-2.51)	0.387
Peripheral Arterial Disease (PAD)	1.08 (0.69-1.69)	0.736	1.11 (0.65-1.89)	0.702	0.98 (0.42-2.25)	0.956
Atrial Fibrillation / Flutter	1.09 (0.85-1.41)	0.498	0.98 (0.72-1.34)	0.894	1.31 (0.83-2.06)	0.242
Chronic Obstructive Pulmonary Disease (COPD)	1.55 (1.12-2.15)	0.009	1.36 (0.91-2.03)	0.134	1.82 (1.04-3.20)	0.037
Anemia+	3.23 (2.32-4.51)	<0.001	4.18 (2.64-6.61)	<0.001	2.07 (1.25-3.41)	0.004

Stage ≥III Chronic Kidney Disease	1.73 (1.26-2.39)	0.001	1.45 (1.01-2.09)	0.044	3.33 (1.60-6.92)	0.001
Non-Ischemic Cardiomyopathy	1.23 (0.95-1.59)	0.117	1.21 (0.88-1.66)	0.234	NA	NA
New York Heart Association (NYHA) Class						
III-IV	3.05 (1.36-6.85)	0.007	1.70 (0.70-4.14)	0.243	7.83 (1.09-56.28)	0.041
IV	1.80 (1.38-2.36)	<0.001	1.81 (1.31-2.51)	<0.001	1.55 (0.99-2.43)	0.055
Serum B-type Natriuretic Peptide Level (continuous)	1.02 (1.01-1.03)	<0.001	1.02 (1.01-1.03)	<0.001	1.03 (1.01-1.05)	<0.001
No Use of Renin Angiotensin System (RAS) Inhibitors	1.72 (1.33-2.23)	<0.001	2.03 (1.48-2.79)	<0.001	1.49 (0.95-2.36)	0.085
No Use of Mineralocorticoid Receptor Antagonists (MRAs)	0.97 (0.71-1.33)	0.856	1.18 (0.83-1.68)	0.349	1.26 (0.58-2.74)	0.558
Furosemide-Equivalent Dose (continuous)	1.01 (1.01-1.08)	<0.001	1.00 (0.99-1.01)	0.127	1.01 (1.01-1.02)	<0.001
Hydralazine + Nitrates Prescription	1.44 (0.64-3.23)	0.382	1.03 (0.42-2.52)	0.941	2.25 (0.31-16.25)	0.420
Oral Anticoagulants Prescription	0.89 (0.69-1.15)	0.389	0.71 (0.52-0.98)	0.035	1.23 (0.79-1.91)	0.367
Cardiac Implantable Electronic Device (CIED)						
Any	1.90 (1.48-2.45)	<0.001	1.09 (0.80-1.49)	0.576	3.40 (2.16-5.36)	<0.001
Cardiac Resynchronization Therapy/Defibrillator (CRT/D)	1.32 (0.92-1.88)	0.126	0.81 (0.56-1.17)	0.262	2.24 (1.03-4.86)	0.042
**Baseline Echocardiographic Variables**						
Functional Mitral Regurgitation	2.16 (1.65-2.83)	<0.001	NA	NA	NA	NA
Severe Mitral Regurgitation	0.99 (0.72-1.35)	0.945	1.19 (0.82-1.74)	0.354	0.85 (0.48-1.49)	0.559
Mitral Regurgitation PISA EROA						
Continuous	2.63 (1.39-6.25)	0.005	1.43 (0.61-3.33)	0.417	5.00 (1.23-20.00)	0.024
≥0.40cm^2	1.14 (0.86-1.49)	0.366	1.08 (0.78-1.51)	0.640	1.18 (0.72-1.89)	0.522
Transmitral Mean Pressure Gradient (TMPG) (continuous)	0.96 (0.88-1.05)	0.411	1.04 (0.93-1.16)	0.514	0.94 (0.81-1.10)	0.465
≥Moderate Mitral Annulus Calcification (MAC)	1.02 (0.67-1.56)	0.913	1.05 (0.58-1.90)	0.873	1.28 (0.69-2.37)	0.432
Left Ventricular Ejection Fraction (LVEF)						
Continuous	0.98 (0.97-0.99)	<0.001	0.99 (0.98-0.99)	0.015	0.98 (0.96-0.99)	0.003
<60%	2.15 (1.59-2.89)	<0.001	1.58 (0.95-2.61)	0.076	1.65 (1.06-2.57)	0.027
<40%	1.88 (1.46-2.42)	<0.001	1.19 (0.87-1.63)	0.287	2.36 (1.32-4.21)	0.004
Left Ventricular End-Systolic Diameter (LVESD)						
Continuous	1.21 (1.11-1.32)	<0.001	1.07 (0.97-1.19)	0.196	1.17 (0.92-1.49)	0.199
≥0.4cm	1.61 (1.25-2.08)	<0.001	1.12 (0.80-1.56)	0.526	1.35 (0.81-2.25)	0.245
Left Ventricular Mass Index, ASE Formula (continuous)	1.00 (0.99-1.01)	0.284	1.00 (0.99-1.01)	0.956	1.00 (0.99-1.01)	0.949
Left Atrial Volume Index (LAVi)						
Continuous	1.00 (1.00-1.01)	0.068	1.00 (1.00-1.01)	0.070	1.00 (0.99-1.01)	0.238
>60cm^3/m^2	1.17 (0.90-1.51)	0.234	1.16 (0.85-1.58)	0.359	1.38 (0.87-2.18)	0.168

≥Moderate Right Ventricular Dysfunction	1.67 (1.23-2.27)	0.001	1.49 (1.04-2.13)	0.029	1.33 (0.70-2.53)	0.385
≥Moderate-Severe Tricuspid Regurgitation	1.79 (1.37-2.35)	<0.001	1.84 (1.34-2.54)	<0.001	1.37 (0.81-2.31)	0.248
TAPSE/PASP						
Continuous	0.13 (0.06-0.31)	<0.001	0.16 (0.05-0.46)	0.001	0.22 (0.06-0.88)	0.032
≤0.37mm/mmHg (total cohort median)	2.28 (1.65-3.14)	<0.001	2.30 (1.56-3.39)	<0.001	1.74 (0.97-3.13)	0.065
**Procedural Variables**						
Acute Heart Failure Presentation, Cardiogenic Shock, Hemodynamic Support, or Urgent Procedure	3.07 (2.38-3.96)	<0.001	3.08 (2.25-4.21)	<0.001	1.96 (1.17-3.29)	0.011
Number of Clips Deployed						
Continuous	1.26 (1.07-1.47)	0.006	1.30 (1.07-1.59)	0.009	1.06 (0.79-1.42)	0.703
≥2	1.26 (0.97-1.63)	0.080	1.28 (0.93-1.77)	0.128	1.01 (0.65-1.58)	0.961
≥3	1.63 (1.18-2.25)	0.013	1.62 (1.11-2.38)	0.013	1.49 (0.82-2.71)	0.187
Use of 1st Generation Device	0.98 (0.75-1.28)	0.859	1.09 (0.76-1.54)	0.645	1.23 (0.79-1.92)	0.357

* Per zip code

+ Anemia was defined as a blood hemoglobin of <13mg/dL in men or <12mg/dL in women.

ASE = American Society of Echocardiography; CABG = coronary artery bypass grafting; CI = confidence interval; EROA = effective regurgitant orifice area; HR = hazard ratio; MI = myocardial infarction; NA = not applicable; PASP = pulmonary arterial systolic pressure; PCI = percutaneous coronary intervention; PISA = proximal isovelocity surface area; TAPSE = tricuspid annular plane systolic excursion

Supplemental Table 3. Baseline Clinical Characteristics of Patients with Functional Mitral Regurgitation According to Race

					P-Value				
	**Whites (N=352)**	**Blacks (N=71)**	**Asians (N=31)**	**Hispanics (N=40)**	**Whites vs Non-Whites**	**Blacks vs Non-Blacks**	**Asians vs Non-Asians**	**Hispanics vs Non-Hispanics**	**Whites vs Blacks**
**Demographic Details**									
Age (years)	76 (69-83)	66 (57-75)	70 (66-79)	69 (59-79)	**<0.001**	**<0.001**	0.363	**0.009**	**<0.001**
Sex Male	220 (62.5)	29 (40.8)	16 (51.6)	22 (55.0)	**0.002**	**0.001**	0.450	0.679	**0.001**
Insurance									
None	4 (1.1)	0 (0.0)	0 (0.0)	1 (2.5)	0.664	0.357	0.561	0.327	0.367
Low-Income	44 (12.5)	20 (28.2)	6 (19.4)	18 (45.0)	**<0.001**	**0.014**	0.817	**<0.001**	**0.001**
Regular / Full	304 (86.4)	51 (71.8)	25 (80.6)	21 (52.5)	**<0.001**	**0.030**	0.938	**<0.001**	**0.002**
Median Yearly Household Income* (K USD)	81.7 (63.6-100.6)	57.1 (50.0-75.4)	78.9 (52.3-95.8)	64.4 (49.6-88.9)	**<0.001**	**<0.001**	0.695	**0.012**	**<0.001**
Percentage of Adults with Academic Degree*	40.6 (24.9-56.1)	26.0 (16.3-35.0)	36.4 (21.5-48.1)	24.3 (18.7-39.3)	**<0.001**	**<0.001**	0.607	**0.003**	**<0.001**
**Comorbidities**									
Obesity (Body Mass Index $\geq$30kg/m^2)	70 (19.9)	18 (25.4)	3 (9.7)	10 (25.0)	0.628	0.268	0.125	0.456	0.301
Diabetes Mellitus	110 (31.3)	24 (33.8)	13 (41.9)	19 (47.5)	0.081	0.969	0.310	0.052	0.673
Hypertension	301 (85.5)	60 (84.5)	22 (71.0)	36 (90.0)	0.499	0.937	**0.037**	0.341	0.827
Smoking History	18 (5.1)	3 (4.2)	0 (0.0)	3 (7.5)	0.678	0.789	0.388	0.431	0.753
Previous MI, PCI, or CABG	205 (58.2)	24 (33.8)	16 (51.6)	25 (62.5)	**0.012**	**<0.001**	0.725	0.299	**<0.001**
Prior Stroke or Transient Ischemic Attack (TIA)	52 (14.8)	12 (16.9)	8 (25.8)	0 (0.0)	0.844	0.548	0.108	**0.006**	0.648
Peripheral Arterial Disease (PAD)	29 (8.2)	6 (8.5)	2 (6.5)	3 (7.5)	0.856	0.906	0.729	0.885	0.953
Atrial Fibrillation / Flutter	200 (56.8)	27 (38.0)	16 (51.6)	17 (42.5)	**0.003**	**0.008**	0.907	0.181	**0.004**
Chronic Obstructive Pulmonary Disease (COPD)	48 (13.6)	16 (22.5)	4 (12.9)	3 (7.5)	0.463	**0.034**	0.810	0.196	0.056

Anemia+	234 (66.5)	51 (71.8)	19 (61.3)	33 (82.5)	0.191	0.480	0.392	**0.043**	0.380
Stage ≥III Chronic Kidney Disease	244 (71.8)	48 (68.6)	26 (86.7)	26 (65.0)	0.941	0.534	0.060	0.328	0.591
**Heart Failure Indices**									
Non-Ischemic Cardiomyopathy	140 (39.8)	45 (63.4)	11 (35.5)	20 (50.0)	**0.005**	**<0.001**	0.339	0.404	**<0.001**
New York Heart Association (NYHA) Class									
II	20 (5.7)	1 (1.4)	2 (6.5)	0 (0.0)	0.088	0.227	0.649	0.243	0.226
III	140 (39.8)	25 (35.2)	8 (25.8)	21 (52.5)	0.719	0.449	0.113	0.074	0.472
IV	189 (53.7)	45 (63.4)	21 (67.7)	19 (47.5)	0.212	0.147	0.155	0.290	0.134
Kansas City Cardiomyopathy Questionnaire 12 Score	35.4 (17.2-55.2)	35.4 (15.0-52.2)	32.3 (12.5-61.5)	26.0 (11.5-43.8)	0.255	0.998	0.980	0.084	0.819
6-Minute Walk Test Distance (m)	244 (104-335)	137 (39-297)	259 (191-382)	219 (122-366)	0.774	0.143	0.302	0.813	0.170
Serum B-type Natriuretic Peptide (pg/mL)	818 (338-1,908)	1,284 (530-2,371)	935 (473-2,720)	887 (320-2,337)	**0.036**	**0.030**	0.303	0.809	**0.020**
**Risk Status**									
STS Score for Mitral Valve Repair	6.2 (3.1-10.1)	5.9 (2.0-9.2)	4.9 (2.2-11.8)	4.7 (2.0-8.7)	**0.025**	0.152	0.718	0.119	0.091
MitraScore	4 (3-5)	4 (3-4)	3 (2-4)	4 (3-4)	0.454	0.973	0.268	0.827	0.833
**Treatment**									
Medications									
Beta Blockers	266 (75.6)	56 (78.9)	22 (71.0)	33 (82.5)	0.538	0.584	0.469	0.337	0.551
Renin Angiotensin System (RAS) Inhibitors	180 (51.1)	42 (59.2)	15 (48.4)	19 (47.5)	0.631	0.181	0.693	0.588	0.217
Mineralocorticoid Receptor Antagonists (MRAs)	94 (26.7)	26 (36.6)	7 (22.6)	14 (35.0)	0.154	0.103	0.448	0.346	0.091
Loop Diuretics									
Frequency	286 (81.3)	57 (80.3)	26 (83.9)	27 (67.5)	0.340	0.978	0.816	**0.036**	0.849
Furosemide-Equivalent Dose (mg/day)	40 (40-80)	40 (40-80)	40 (40-45)	80 (40-160)	0.193	0.466	0.279	**0.019**	0.373
Anti-Arrhythmics	91 (25.9)	17 (23.9)	8 (25.8)	7 (17.5)	0.440	0.841	0.904	0.259	0.737
Hydralazine + Nitrates	11 (3.1)	7 (9.9)	1 (3.2)	2 (5.0)	0.051	**0.021**	0.770	0.684	**0.019**

Oral Anticoagulants	176 (60.0)	28 (39.4)	17 (54.8)	8 (20.0)	**0.011**	0.206	0.328	**<0.001**	0.104
Cardiac Implantable Electronic Device (CIED)									
Total	160 (45.5)	43 (60.6)	13 (41.9)	13 (32.5)	0.527	**0.009**	0.610	0.067	**0.020**
Pacemaker	44 (12.5)	5 (7.0)	4 (12.9)	0 (0.0)	**0.045**	0.278	0.762	**0.015**	0.190
Implantable Cardioverter Defibrillator (ICD)	33 (9.4)	16 (22.5)	2 (6.5)	5 (12.5)	**0.030**	**0.001**	0.560	0.795	**0.002**
CRT/Defibrillator (CRT/D)	83 (23.6)	22 (31.0)	7 (22.6)	8 (20.0)	0.561	0.155	0.819	0.509	0.188

Data are presented as number (percentage) or median (interquartile range), where appropriate.

* Per zip code

+ Anemia was defined as a blood hemoglobin of <13mg/dL in men or <12mg/dL in women.

CABG = coronary bypass artery grafting; CRT = cardiac resynchronization therapy; GFR = glomerular filtration rate; MI = myocardial infarction; PCI = percutaneous coronary intervention; STS = Society of Thoracic Surgeons; USD = United States Dollars

Supplemental Table 4. Baseline Echocardiographic Data of Patients with Functional Mitral Regurgitation According to Race

					P-Value				
	**Whites (N=352)**	**Blacks (N=71)**	**Asians (N=31)**	**Hispanics (N=40)**	**Whites vs Non-Whites**	**Blacks vs Non-Blacks**	**Asians vs Non-Asians**	**Hispanics vs Non-Hispanics**	**Whites vs Blacks**
**Mitral Valve**									
Mitral Regurgitation Severity									
Moderate-Severe	85 (24.3)	13 (18.3)	3 (9.7)	8 (20.0)	0.074	0.399	0.084	0.732	0.277
Severe	261 (74.6)	57 (80.3)	28 (90.3)	31 (77.5)	0.091	0.431	0.063	0.892	0.307
Mitral Regurgitation PISA EROA (cm^2)	0.33 (0.25-0.45)	0.33 (0.25-0.40)	0.34 (0.24-0.42)	0.30 (0.22-0.29)	0.329	0.732	0.978	0.245	0.585
Mitral Regurgitation PISA RVol (mL)	49.9 (34.6-63.1)	49.0 (35.6-59.1)	50.1 (36.8-64.5)	39.3 (30.4-55.5)	0.719	0.907	0.511	0.160	1.000
Transmitral Mean Pressure Gradient (TMPG) (mmHg	2 (2-3)	2 (3-4)	2 (2-4)	2 (2-3)	0.531	0.178	0.864	0.389	0.194
≥Moderate Mitral Annulus Calcification (MAC)	34 (9.7)	3 (4.2)	1 (3.2)	1 (2.5)	**0.022**	0.215	0.498	0.352	0.139
**Left Heart**									
Left Ventricular Ejection Fraction (LVEF) (%)	35 (21-54)	25 (16-40)	38 (25-50)	35 (17-45)	**0.009**	**<0.001**	0.283	0.445	**<0.001**
Left Ventricular End-Systolic Diameter (LVESD) (cm)	4.6 (3.6)	5.4 (4.3-6.3)	4.8 (3.8-5.3)	4.8 (3.7-5.8)	**0.003**	**<0.001**	0.636	0.779	**<0.001**
Left Ventricular Mass Index, ASE Formula (gr/m^2)	133.3 (106.7-159.4)	132.1 (113.4-165.5)	126.0 (109.6-181.2)	142.0 (116.1-160.7)	0.154	0.382	0.682	0.379	0.292
Left Atrial Volume Index (LAVi) (cm^3/m^2)	55.2 (43.8-72.9)	50.2 (40.0-73.2)	54.0 (46.0-70.0)	60.1 (49.7-75.2)	0.958	0.395	0.724	0.177	0.470
**Right Heart**									
≥Moderate Right Ventricular Dysfunction	71 (24.8)	22 (36.1)	11 (42.3)	13 (32.5)	**0.018**	0.146	0.102	0.538	0.072
≥Moderate-Severe Tricuspid Regurgitation	76 (21.7)	22 (31.0)	10 (32.3)	21 (52.5)	**<0.001**	0.318	0.425	**<0.001**	0.089
**Right Ventricular (RV)-Pulmonary Arterial (PA) Coupling**									
Tricuspid Annular Plane Systolic Excursion (TAPSE) (mm)	16 (13-18)	16 (14-21)	16 (12-18)	15 (13-19)	0.976	0.328	0.448	0.568	0.420
Pulmonary Arterial Systolic Pressure (PASP) (mmHg)	47 (35-58)	51 (35-64)	45 (32-54)	50 (39-64)	0.147	0.180	0.511	0.203	0.152

TAPSE/PASP (mm/mmHg)	0.34 (0.25-0.50)	0.30 (0.22-0.50)	0.33 (0.26-0.50)	0.31 (0.22-0.45)	0.209	0.463	0.944	0.208	0.375

Data are presented as number (percentage) or median (interquartile range), where appropriate.

ASE = American Society of Echocardiography; EROA = effective regurgitant orifice area; PISA = proximal isovelocity surface area; RVol = regurgitant volume

Supplemental Table 5. Procedural Details and Results Observed in Patients with Functional Mitral Regurgitation According to Race

	Whites (N=352)	Blacks (N=71)	Asians (N=31)	Hispanics (N=40)	P-Value<br>Whites vs Non-Whites	Blacks vs Non-Blacks	Asians vs Non-Asians	Hispanics vs Non-Hispanics	Whites vs Blacks
**Presentation to Procedure**									
Acute Decompensated Heart Failure	71 (20.2)	17 (23.9)	4 (12.9)	12 (30.0)	0.449	0.518	0.250	0.148	0.475
Cardiogenic Shock	18 (5.1)	3 (4.2)	2 (6.5)	1 (2.5)	0.678	0.789	0.657	0.710	0.753
Medical and/or Mechanical Hemodynamic Support	28 (8.0)	7 (9.9)	4 (12.9)	4 (10.0)	0.352	0.709	0.333	0.768	0.595
Urgent Procedure	89 (25.3)	18 (25.4)	8 (25.8)	7 (17.5)	0.633	0.890	0.882	0.271	0.990
**Procedural Aspects**									
Number of Clips Deployed									
0 (Aborted / Not Deployed)	12 (3.4)	1 (1.4)	0 (0.0)	0 (0.0)	0.122	0.704	0.344	0.613	0.705
1	138 (39.2)	19 (26.8)	16 (51.6)	17 (42.5)	0.593	**0.029**	0.120	0.584	**0.048**
2	150 (42.6)	35 (49.3)	14 (45.2)	18 (45.0)	0.354	0.325	0.886	0.887	0.300
≥2	202 (57.4)	51 (71.8)	15 (48.4)	23 (57.5)	0.279	**0.017**	0.219	0.850	**0.024**
≥3	52 (14.8)	16 (22.5)	1 (3.2)	5 (12.5)	0.839	0.054	0.067	0.647	0.104
Median	1 (1-2)	2 (1-2)	1 (1-2)	2 (1-2)	0.272	**0.009**	0.117	0.866	**0.015**
Device Generation									
1	82 (23.3)	17 (23.9)	5 (16.1)	13 (32.5)	0.749	0.956	0.307	0.171	0.906
2	128 (36.4)	21 (29.6)	10 (32.3)	19 (47.5)	0.809	0.221	0.651	0.115	0.275
3	103 (29.3)	21 (29.6)	10 (32.3)	2 (5.0)	0.175	0.676	0.543	**0.001**	0.957
4	39 (11.1)	12 (16.9)	6 (19.4)	6 (15.0)	0.079	0.257	0.264	0.657	0.169
Intervention Site									

A1P1	5 (1.4)	0 (0.0)	0 (0.0)	0 (0.0)	0.328	1.000	1.000	1.000	0.595
A2P2	346 (98.3)	70 (98.6)	31 (100.0)	40 (100.0)	0.679	0.995	0.491	0.429	0.858
A3P3	18 (5.1)	2 (2.8)	1 (3.2)	1 (2.5)	0.263	0.755	0.732	0.532	0.549
Total Duration (min)	109 (87-137)	108 (86-141)	98 (78-136)	119 (92-145)	0.927	0.939	0.136	0.282	0.968
Fluoroscopy Duration (min)	20 (14-26)	18 (15-31)	16 (12-26)	18 (14-27)	0.571	0.733	0.234	0.747	0.885
Conversion to Surgery	0 (0.0)	0 (0.0)	0 (0.0)	0 (0.0)	NA	NA	NA	NA	NA
**Echocardiographic and Hemodynamic Effects**									
Mitral Regurgitation Severity Reduction to ≤Mild									
Immediately after Clip Deployment	233 (66.2)	50 (70.4)	20 (64.5)	28 (70.0)	0.546	0.508	0.761	0.674	0.490
At Discharge	266 (78.9)	52 (73.2)	21 (72.4)	28 (70.0)	0.109	0.422	0.551	0.276	0.293
Transmitral Mean Pressure Gradient (mmHg)									
Immediately after Clip Deployment	3 (2-4)	3 (2-4)	3 (2-4)	3 (2-5)	0.317	0.452	0.184	0.625	0.392
At 1-Month	4 (3-5)	5 (3-6)	3 (3-5)	4 (3-5)	0.962	0.076	0.127	0.373	0.131
Pulmonary Venous Flow Pattern Normalization on ≥1 Side	166 (53.9)	42 (66.7)	11 (44.0)	18 (52.9)	0.419	**0.046**	0.250	0.790	0.063
Delta V wave (mmHg)	-8 (-18-0)	-13 (-27-[-5])	-13 (-30-0)	-13 (-27-[-2])	**0.001**	**0.011**	0.283	0.171	**0.003**
Delta Mean Left Atrial Pressure (LAP) (mmHg)	-3 (-9-1)	-5 (-13-[-1])	-8 (-15-0)	-6 (-11-[-1])	**0.001**	**0.045**	0.192	0.131	**0.015**
Delta Mean Pulmonary Arterial Pressure (PAP) (mmHg)	-1 (-7-3)	-2 (-8-4)	0 (-4-6)	-3 (-11-1)	0.304	0.667	0.440	0.120	0.549
**Post-Procedural Course**									
Intensive Cardiac Unit (ICU) Stay Duration (hours)	19.2±75.1	51.4±185.6	17.7±62.5	6.9±20.6	0.309	0.137	0.764	0.278	0.154
Hospitalization Length (days)	1 (1-8)	4 (1-10)	1 (1-5)	3 (1-10)	0.110	0.088	0.308	0.173	0.075
Discharge Home	317 (93.0)	65 (94.2)	30 (96.8)	36 (92.3)	0.428	0.806	0.246	0.731	0.709
Blood Transfusion or Any 1-Month Non-Fatal Adverse Event*	41 (11.6)	15 (21.1)	3 (9.7)	6 (15.0)	0.118	**0.038**	0.784	0.719	**0.032**
**Medical Treatment at 1-Month**									
Beta Blockers	216 (78.8)	41 (70.7)	18 (75.0)	25 (78.1)	0.450	0.395	0.932	0.949	0.369

Renin Angiotensin System (RAS) Inhibitors	151 (55.3)	31 (54.4)	11 (45.8)	16 (50.0)	0.475	0.968	0.399	0.623	0.898
Mineralocorticoid Receptor Antagonists (MRAs)	77 (28.1)	21 (36.2)	7 (29.2)	10 (30.3)	0.330	0.229	0.965	0.922	0.219
Loop Diuretics	214 (78.1)	46 (79.3)	17 (70.8)	25 (75.8)	0.733	0.740	0.409	0.787	0.839
Anti-Arrhythmics	70 (25.5)	16 (28.1)	8 (33.3)	8 (24.2)	0.607	0.741	0.418	0.780	0.693
Hydralazine + Nitrates	7 (2.5)	6 (10.3)	1 (4.0)	2 (6.1)	**0.023**	**0.019**	0.992	0.633	**0.013**
Oral Anticoagulants	151 (55.1)	21 (36.2)	12 (50.0)	9 (27.3)	**0.001**	**0.027**	0.959	**0.007**	**0.009**

Data are presented as number (percentage) or median (interquartile range), or mean±standard deviation, where appropriate

* 1-month non-fatal adverse events included the following: tamponade, cardiac arrest, myocardial infarction, stroke, transient ischemic attack, MVARC bleeding, and vascular complications.

MVARC = mitral valve academy research consortium; NA = not applicable

Supplemental Table 6. Outcomes and Trends following Transcatheter Edge-to-Edge Repair for Functional Mitral Regurgitation According to Race

					P-Value				
	**Whites (N=352)**	**Blacks (N=71)**	**Asians (N=31)**	**Hispanics (N=40)**	**Whites vs Non-Whites**	**Blacks vs Non-Blacks**	**Asians vs Non-Asians**	**Hispanics vs Non-Hispanics**	**Whites vs Blacks**
**Primary Outcome**									
All-Cause Mortality or Heart Failure Hospitalizations at 1-Year	103 (29.3)	31 (43.7)	10 (32.3)	17 (42.5)	**0.013**	**0.031**	0.967	0.163	**0.017**
Event-Free Survival (days)	279±8	239±18	278±24	264±21	**0.040**	**0.038**	0.959	0.417	**0.027**
**Secondary Outcomes**									
Clinical									
All-Cause Mortality at 1-Year	47 (13.4)	14 (19.7)	3 (9.7)	7 (17.5)	0.309	0.165	0.600	0.556	0.164
Heart Failure Hospitalizations at 1-Year	63 (17.9)	23 (32.4)	8 (25.8)	12 (30.0)	**0.002**	**0.015**	0.542	0.170	**0.006**
New York Heart Association Class ≤II									
At 1-Month	177 (70.8)	31 (62.0)	17 (73.9)	23 (82.1)	0.925	0.147	0.723	0.164	0.218
At 1-Year	118 (67.4)	17 (51.5)	16 (88.9)	13 (68.4)	0.797	**0.043**	**0.040**	0.886	0.079
Echocardiographic									
Mitral Regurgitation Severity ≤Mild									
At 1-Month	142 (62.0)	27 (13.8)	12 (52.2)	15 (60.0)	0.386	0.644	0.397	0.958	0.559
At 1-Year	65 (50.8)	16 (59.3)	6 (46.2)	7 (36.8)	0.836	0.312	0.758	0.217	0.423
Mitral Regurgitation Severity ≤Moderate									
At 1-Month	219 (98.2)	41 (93.2)	23 (100.0)	25 (100.0)	0.421	0.060	0.453	0.432	0.090
At 1-Year	124 (96.9)	25 (92.6)	13 (100.0)	16 (84.2)	0.143	0.620	0.401	0.051	0.280

Left Ventricular Mass Index (gr/m^2)									
At 1-Month	130.7 (100.4-154.5)	132.1 (108.8-177.0)	142.7 (127.7-157.6)	144.2 (107.2-153.0)	0.095	0.291	0.189	0.861	0.217
At 1-Year	115.0 (92.3-151.4)	126.4 (105.6-152.6)	133.4 (104.6-155.7)	143.4 (115.7-174.5)	**0.035**	0.537	0.702	**0.031**	0.292
Combined Clinical and Echocardiographic									
New York Heart Association Class ≤II or Mitral Regurgitation Severity ≤Mild									
At 1-Month	211 (89.0)	39 (79.6)	17 (77.3)	25 (92.6)	0.112	0.086	0.179	0.552	0.070
At 1-Year	135 (84.9)	24 (77.4)	16 (100.0)	14 (73.7)	0.565	0.294	0.082	0.199	0.296
**Trends**									
Absolute Change in New York Heart Association Class									
At 1-Month	-1.3±0.9	-1.2±0.9	-1.7±0.7	-1.3±0.7	0.827	0.349	**0.038**	0.955	0.374
At 1-Year	-1.3±1.0	-1.1±0.7	-1.6±0.8	-1.2±0.9	0.723	0.093	0.120	0.694	0.131
P-Value for 1-Year vs Baseline	**<0.001**	**<0.001**	**<0.001**	**<0.001**	NA	NA	NA	NA	NA
Relative Change in Left Ventricular Mass Index (%)									
At 1-Month	-1.3 (-16.7-15.5)	9.0 (-8.9-27.4)	-1.6 (-12.3-26.7)	0.2 (-10.5-14.5)	0.074	**0.029**	0.491	0.606	**0.025**
At 1-Year	-7.3 (-22.7-19.5)	0.6 (-24.8-23.5)	-4.5 (-26.3-46.4)	-8.1 (-15.9-32.0)	0.321	0.763	0.760	0.351	0.617

Data are presented as number (percentage), median (interquartile range), or mean±standard deviation, where appropriate.

NA = not applicable

Supplemental Figure 3. One-Year Cumulative Incidence of the Combined Outcome of All-Cause Mortality or Heart Failure Hospitalizations Following Transcatheter Edge-to-Edge Repair for Functional Mitral Regurgitation According to Race

A All Races

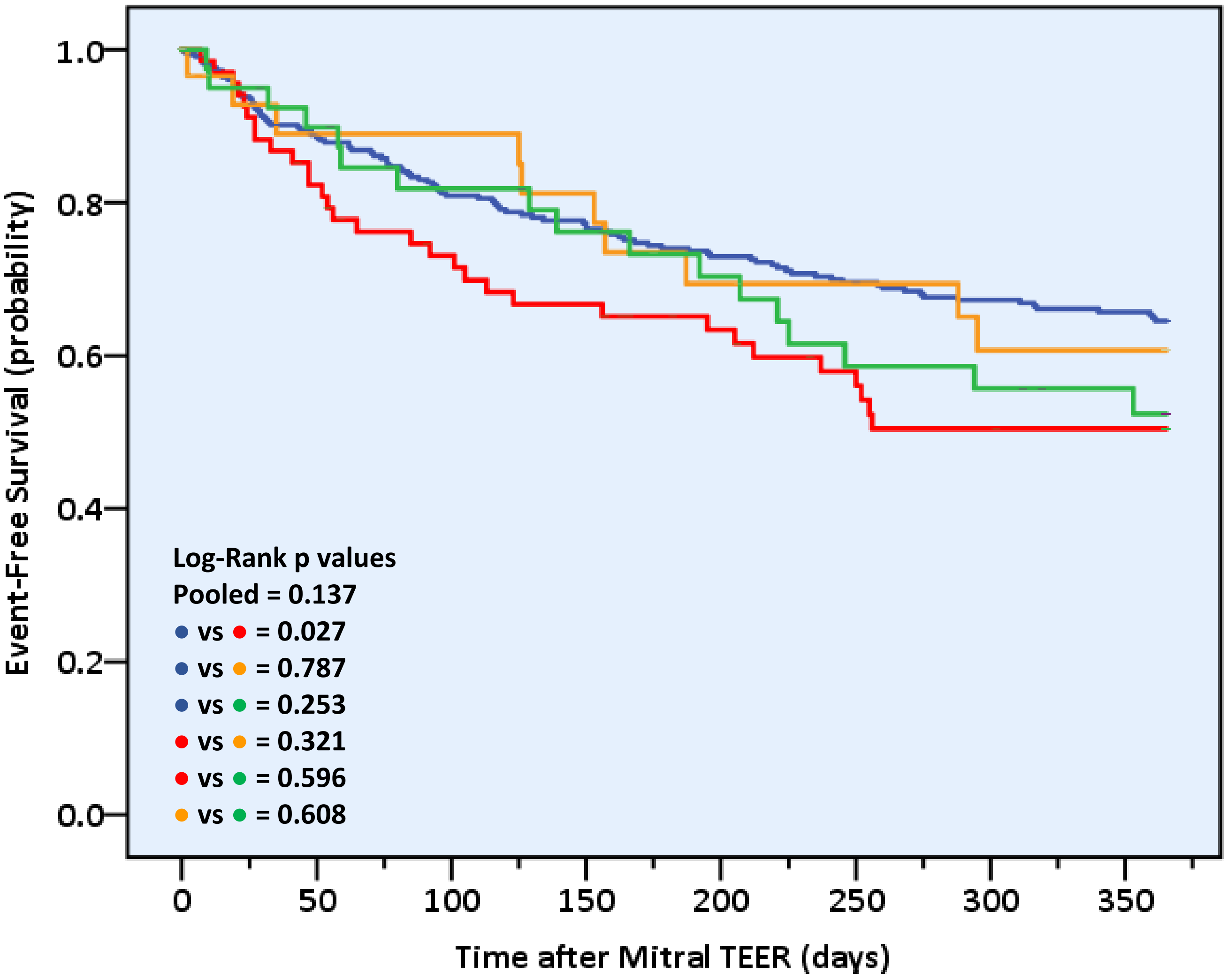

No. at Risk								
Whites	352	266	229	212	193	183	176	165
Blacks	71	55	46	42	36	30	27	26
Asians	31	23	23	21	17	16	14	14
Hispanics	40	35	30	27	24	20	19	17

B Whites vs non-Whites

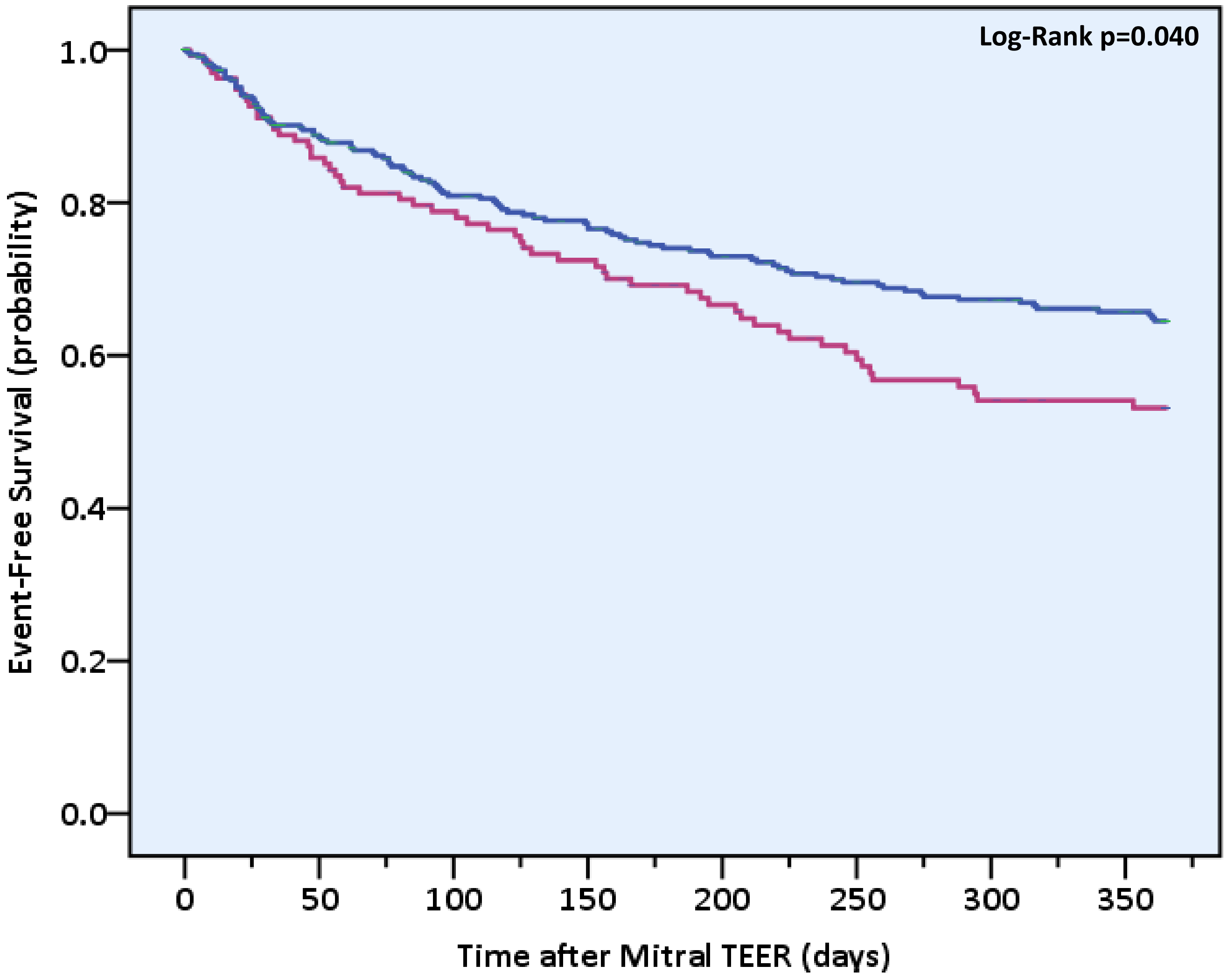

No. at Risk								
Whites	352	266	229	212	193	183	176	165
Non-Whites	142	113	99	90	77	66	60	57

C Blacks vs non-Blacks

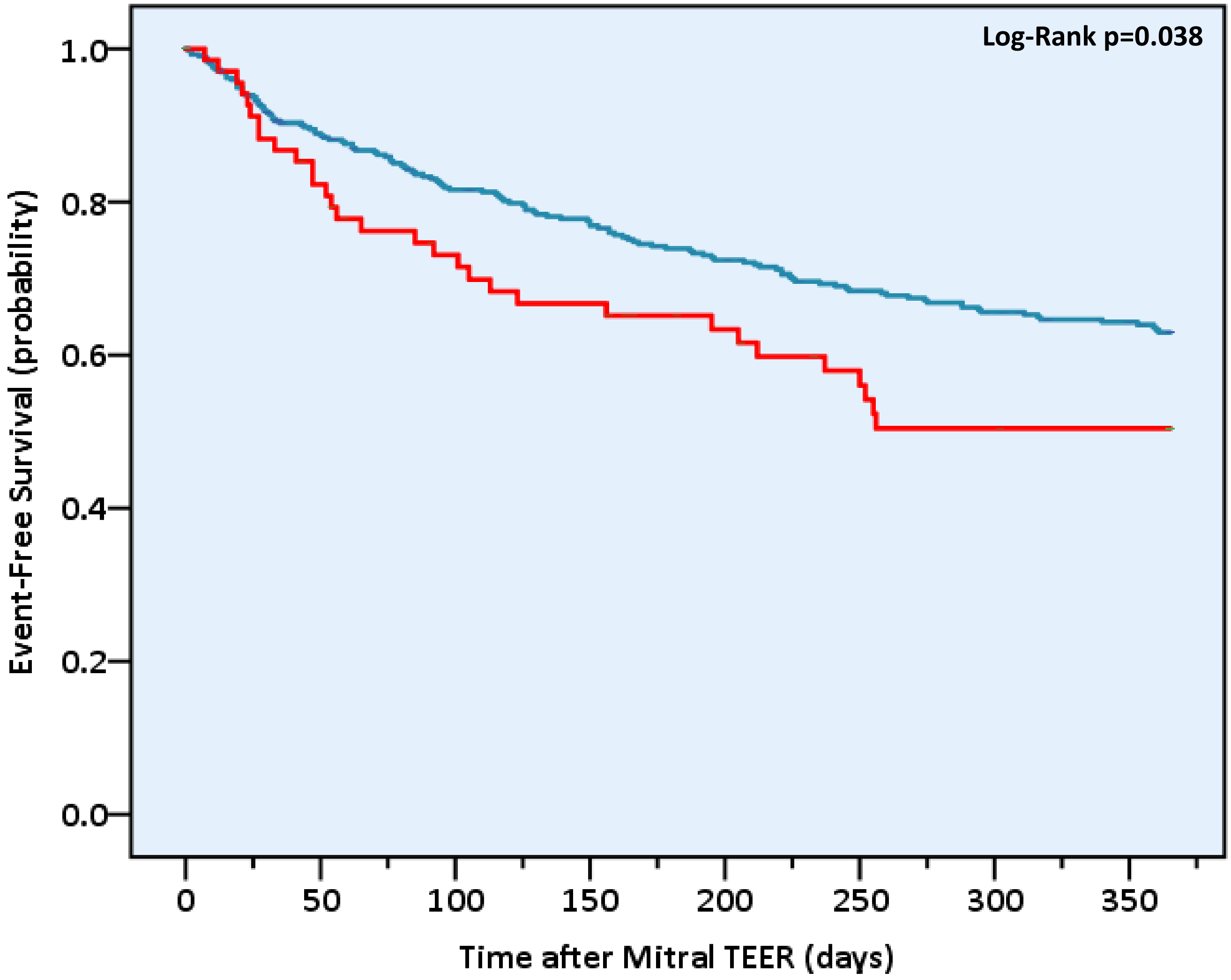

No. at Risk								
Non-Blacks	423	324	282	260	239	219	209	191
Blacks	71	55	46	42	36	30	27	26

D Asians vs non-Asians

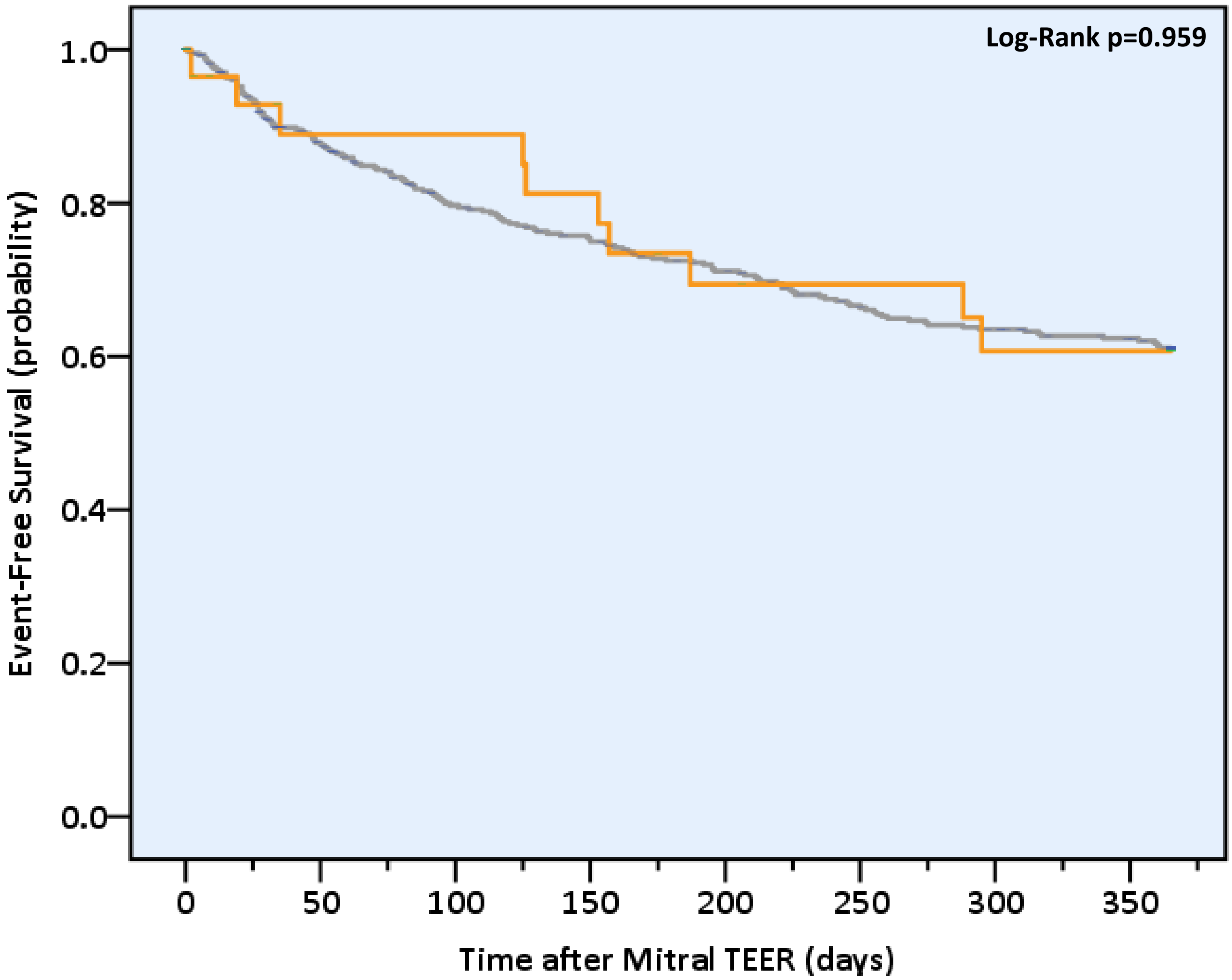

**No. at Risk**								
Non-Asians	463	356	305	281	258	233	222	208
Asians	31	23	23	21	17	16	14	14

E Hispanics vs non-Hispanics

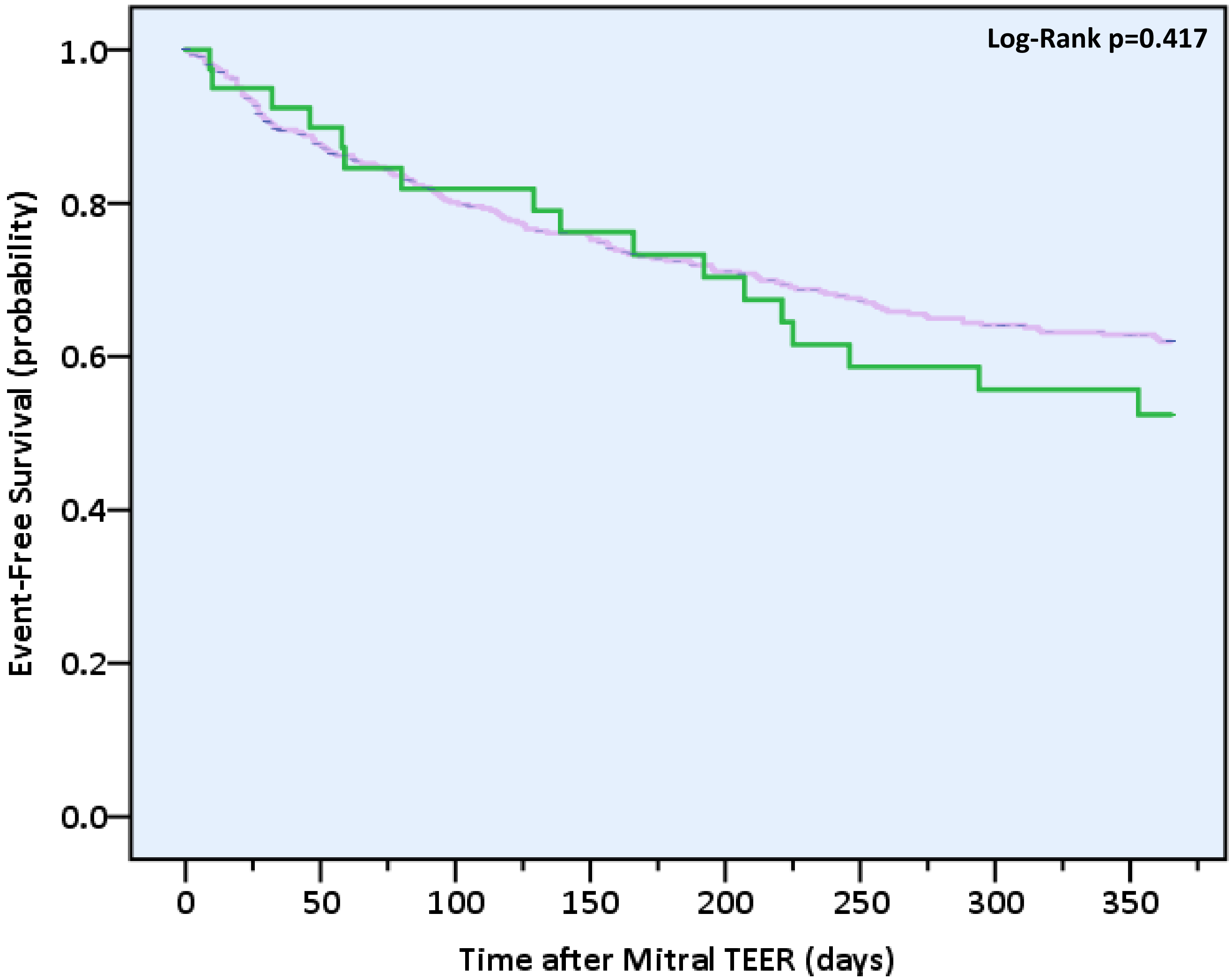

No. at Risk								
Non-Hispanics	454	344	298	275	251	229	217	205
Hispanics	40	35	30	27	24	20	19	17

TEER = transcatheter edge-to-edge repair

Supplemental Figure 4. One-Year Cumulative Incidence of All-Cause Mortality Following Transcatheter Edge-to-Edge Repair for Functional Mitral Regurgitation According to Race

A All Races

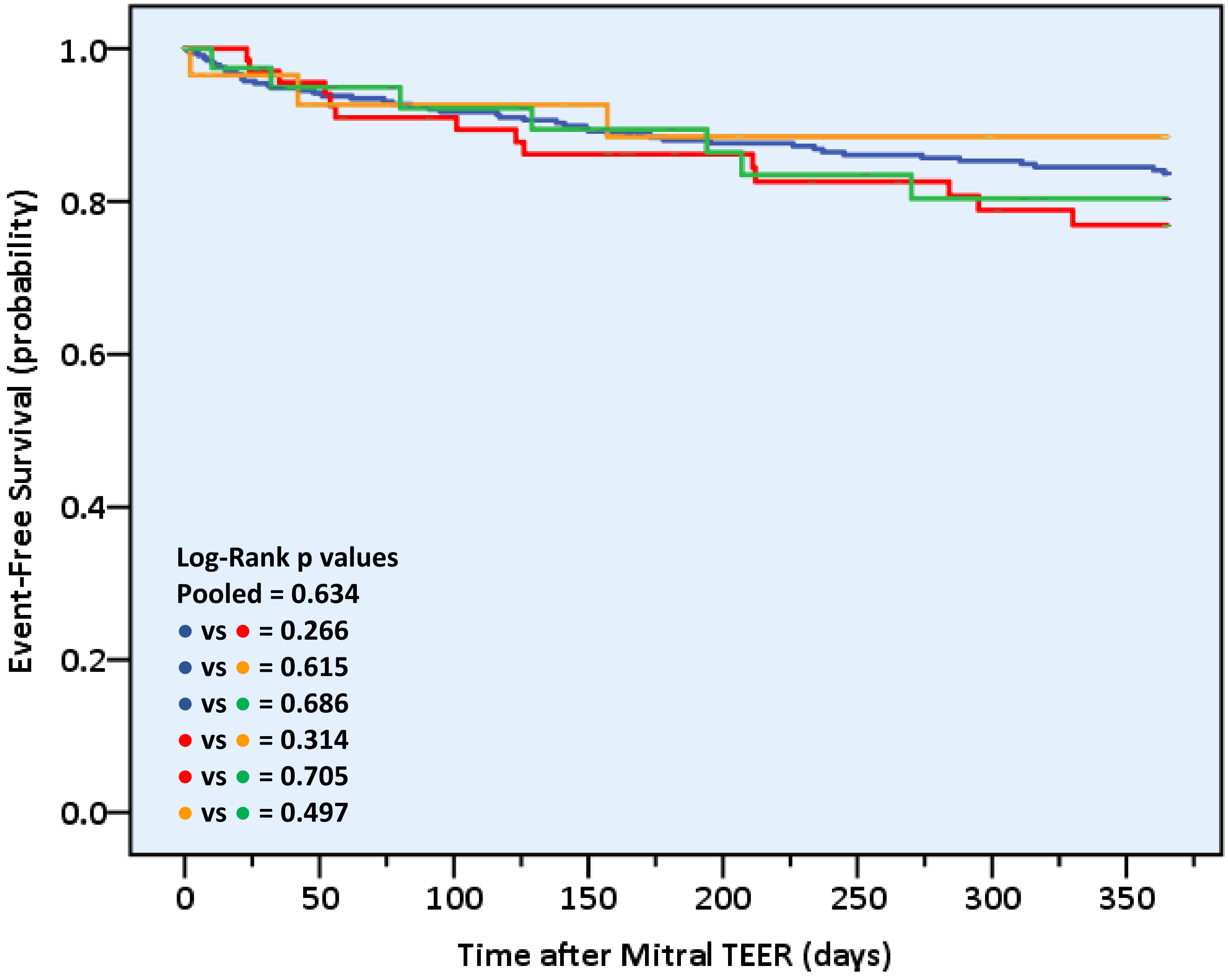

No. at Risk								
Whites	352	282	259	240	229	218	214	205
Blacks	71	63	57	54	49	45	42	40
Asians	31	23	23	23	20	19	18	18
Hispanics	40	37	34	32	29	28	26	24

B Whites vs non-Whites

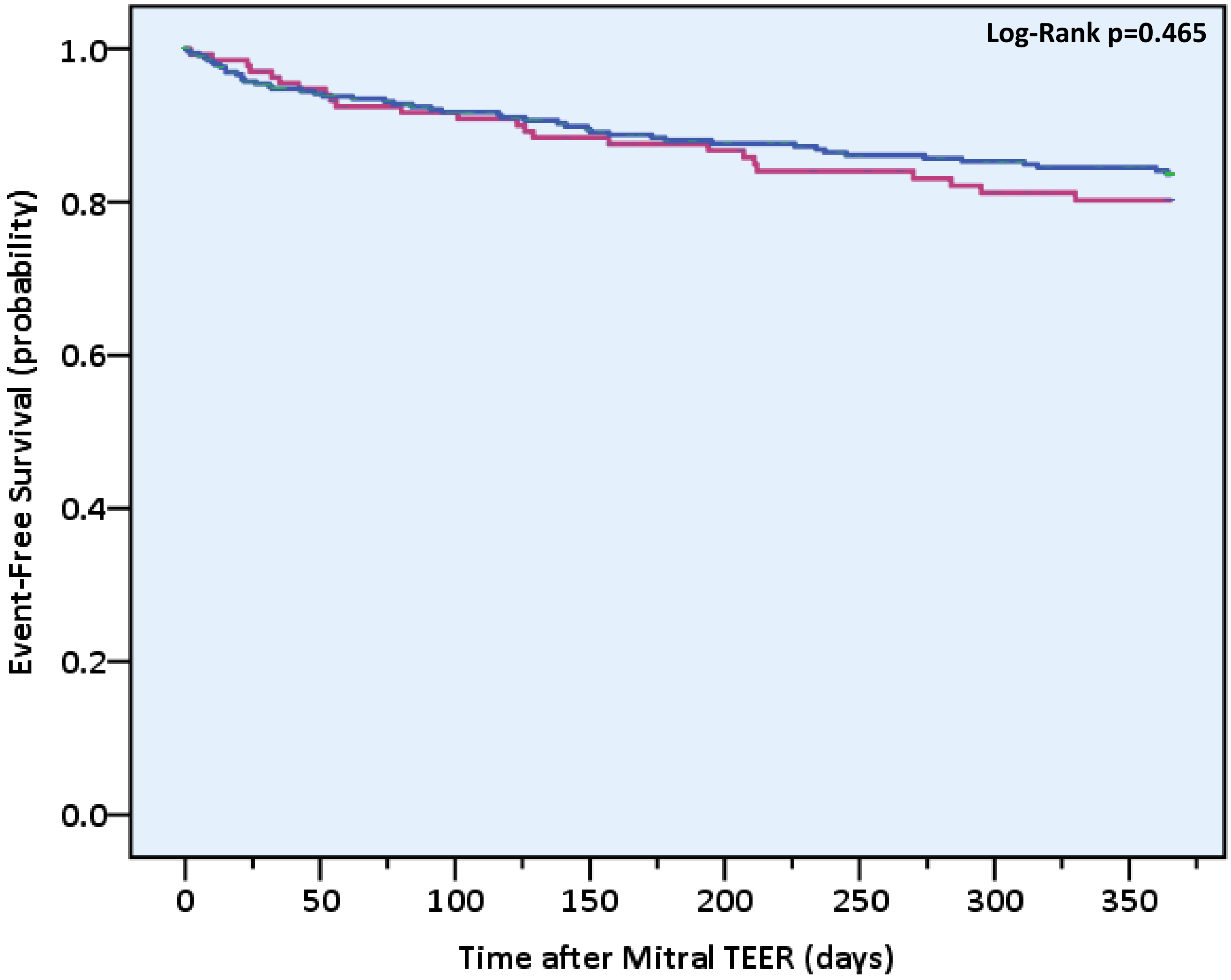

No. at Risk								
Whites	352	282	259	240	229	218	214	205
Non-Whites	142	123	114	108	98	92	86	82

C Blacks vs non-Blacks

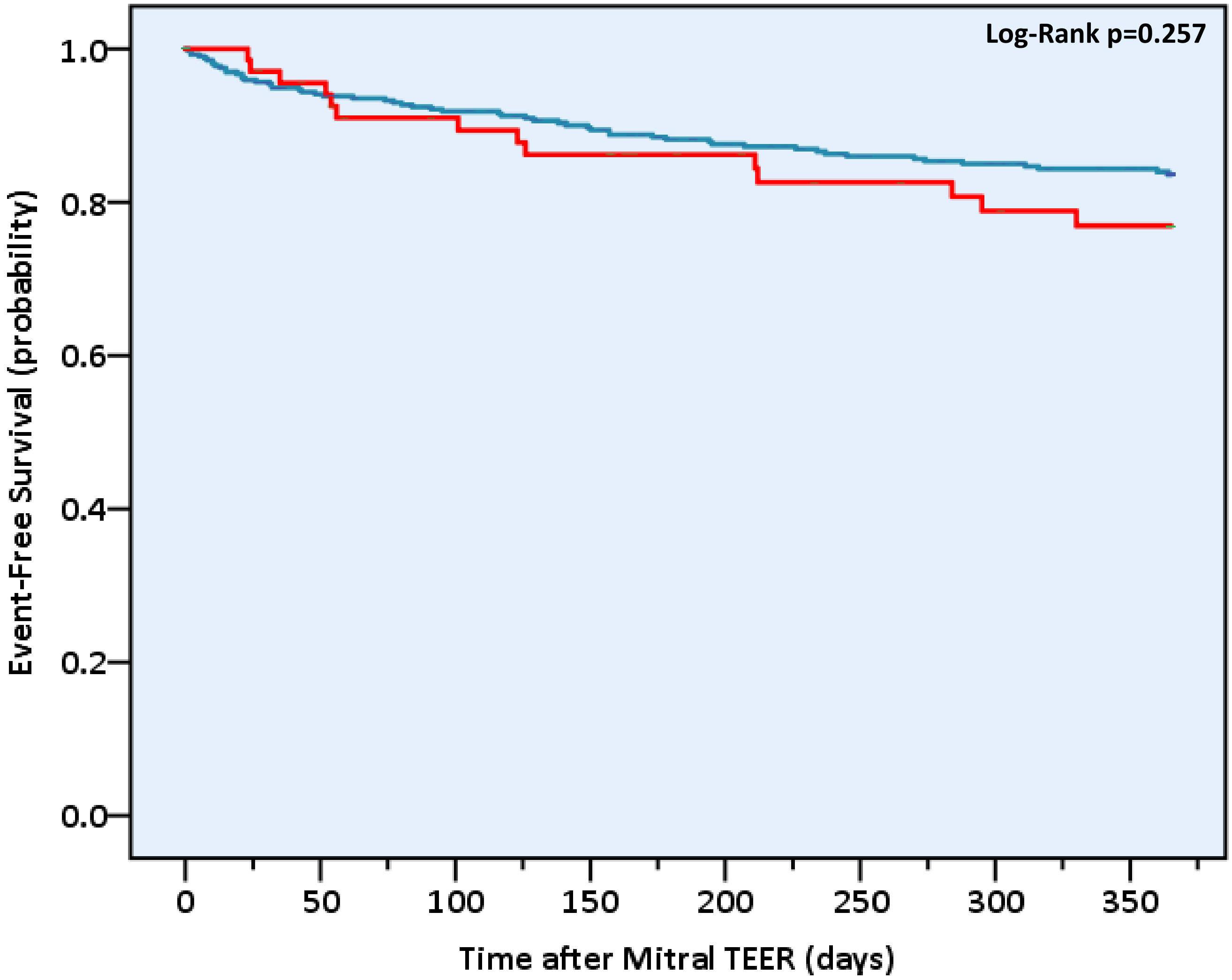

No. at Risk								
Non-Blacks	423	342	316	294	278	265	258	247
Blacks	71	63	57	54	49	45	42	40

TEER = transcatheter edge-to-edge repair

Supplemental Figure 5. One-Year Cumulative Incidence of Heart Failure Hospitalizations Following Transcatheter Edge-to-Edge Repair for Functional Mitral Regurgitation According to Race

A All Races

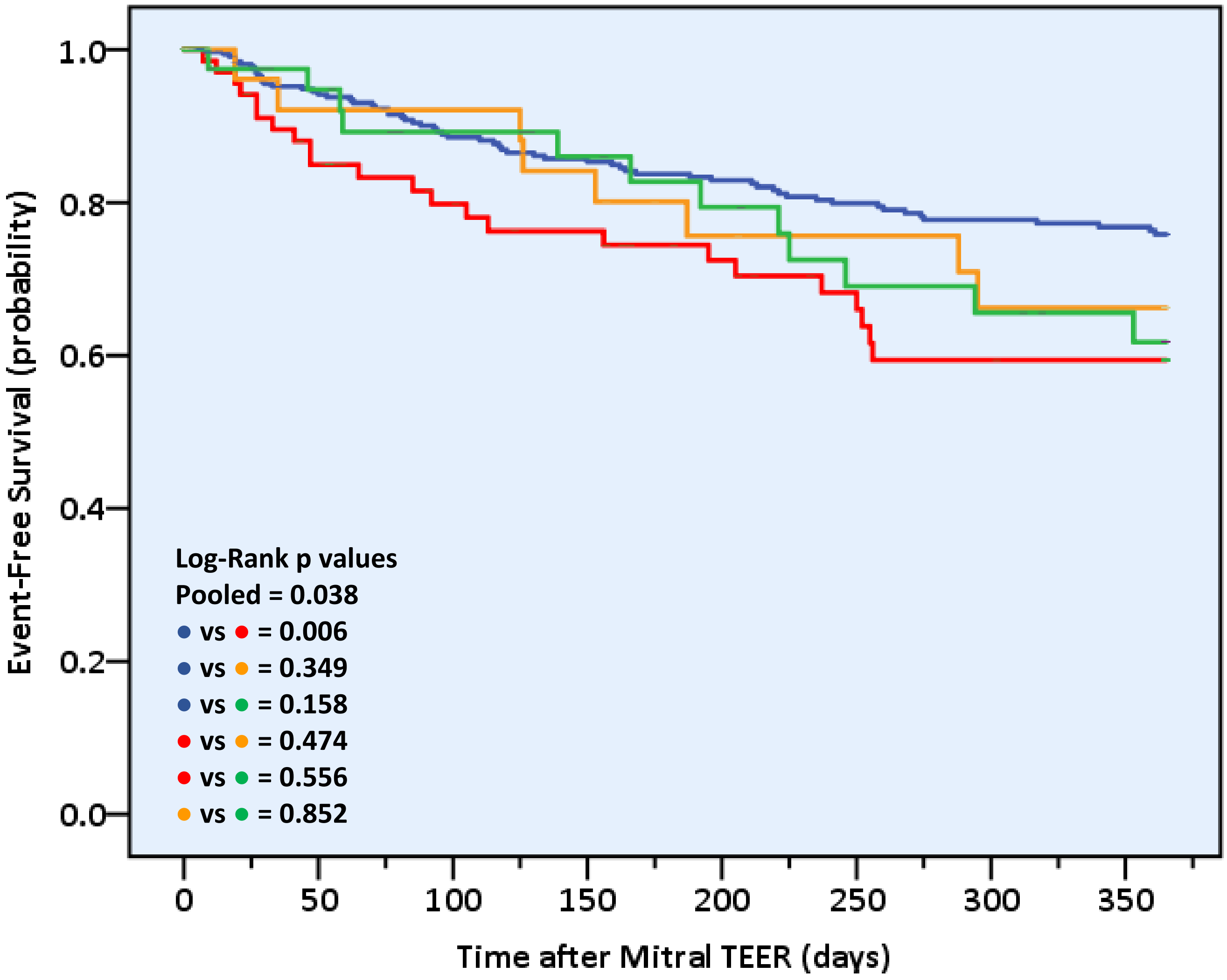

No. at Risk								
Whites	352	266	229	212	198	183	176	165
Blacks	71	55	46	42	36	30	27	26
Asians	31	23	23	21	17	16	14	14
Hispanics	40	35	30	27	24	20	19	17

B Whites vs non-Whites

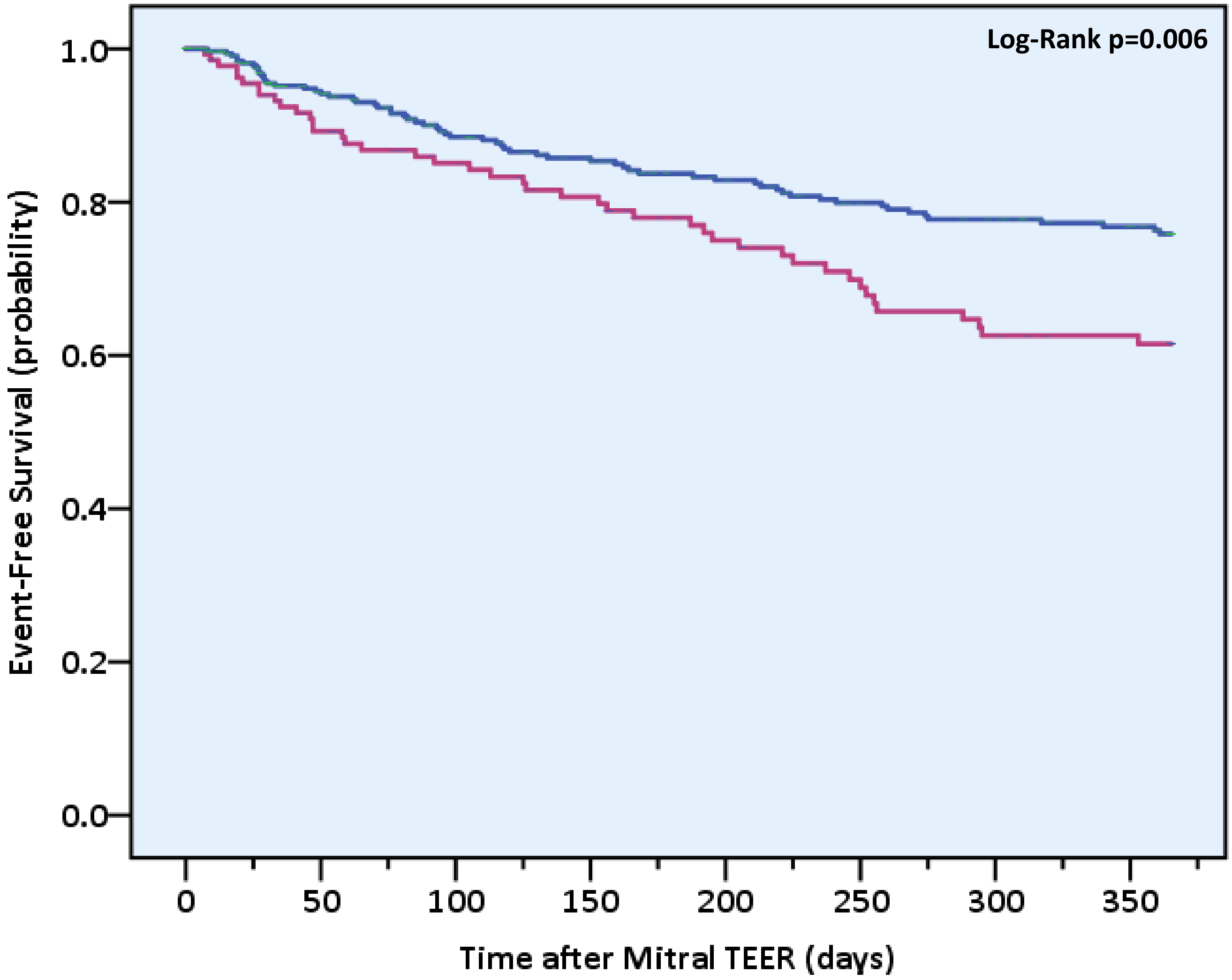

**No. at Risk**								
**Whites**	352	266	229	212	198	183	176	165
**Non-Whites**	142	113	99	90	77	66	60	57

C Blacks vs non-Blacks

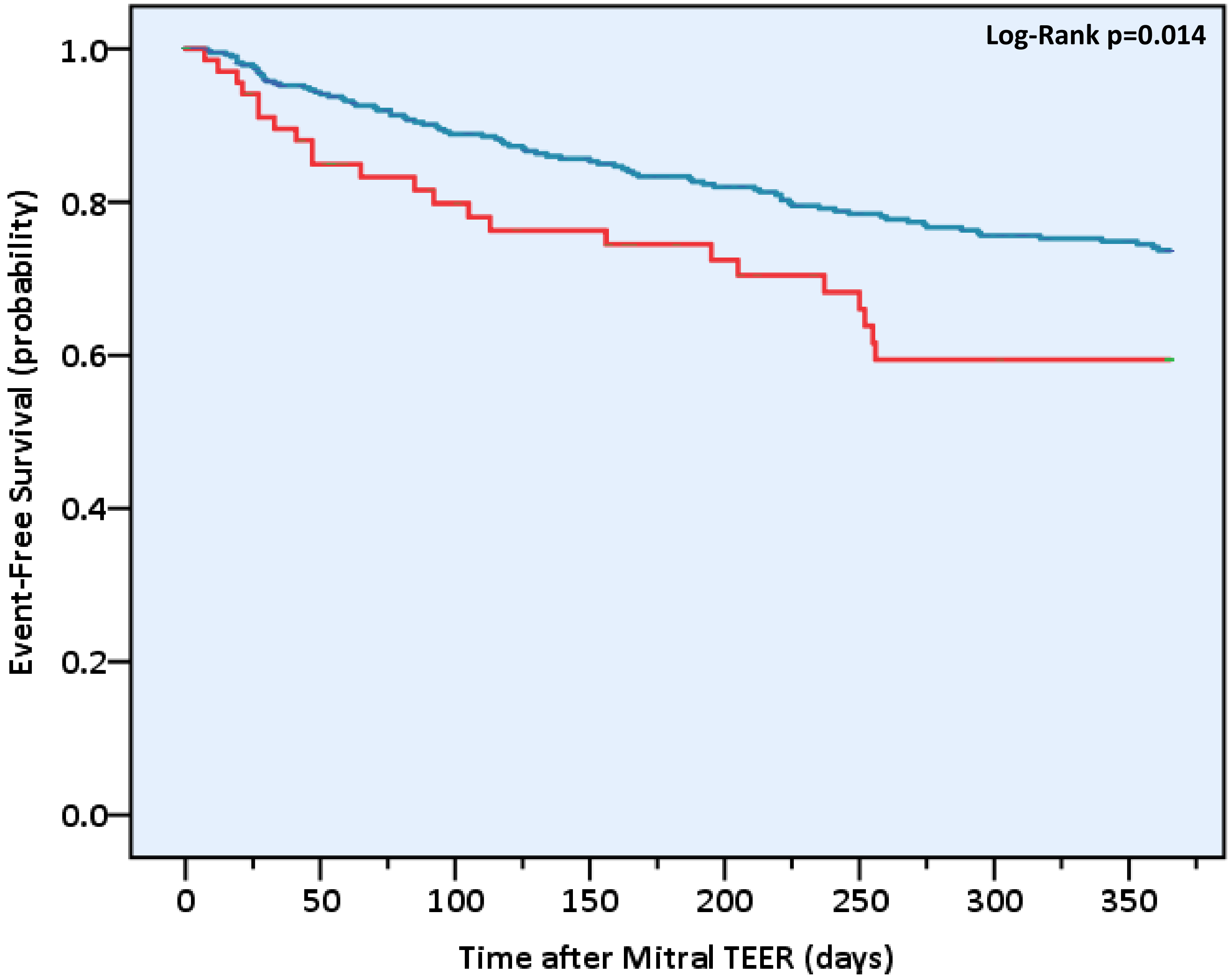

**No. at Risk**								
**Non-Blacks**	423	324	282	273	223	219	209	196
**Blacks**	71	55	46	42	36	30	27	26

TEER = transcatheter edge-to-edge repair

Supplemental Figure 6. Functional Status at Baseline and Following Transcatheter Edge-to-Edge Repair for Functional Mitral Regurgitation According to Race

A All Races

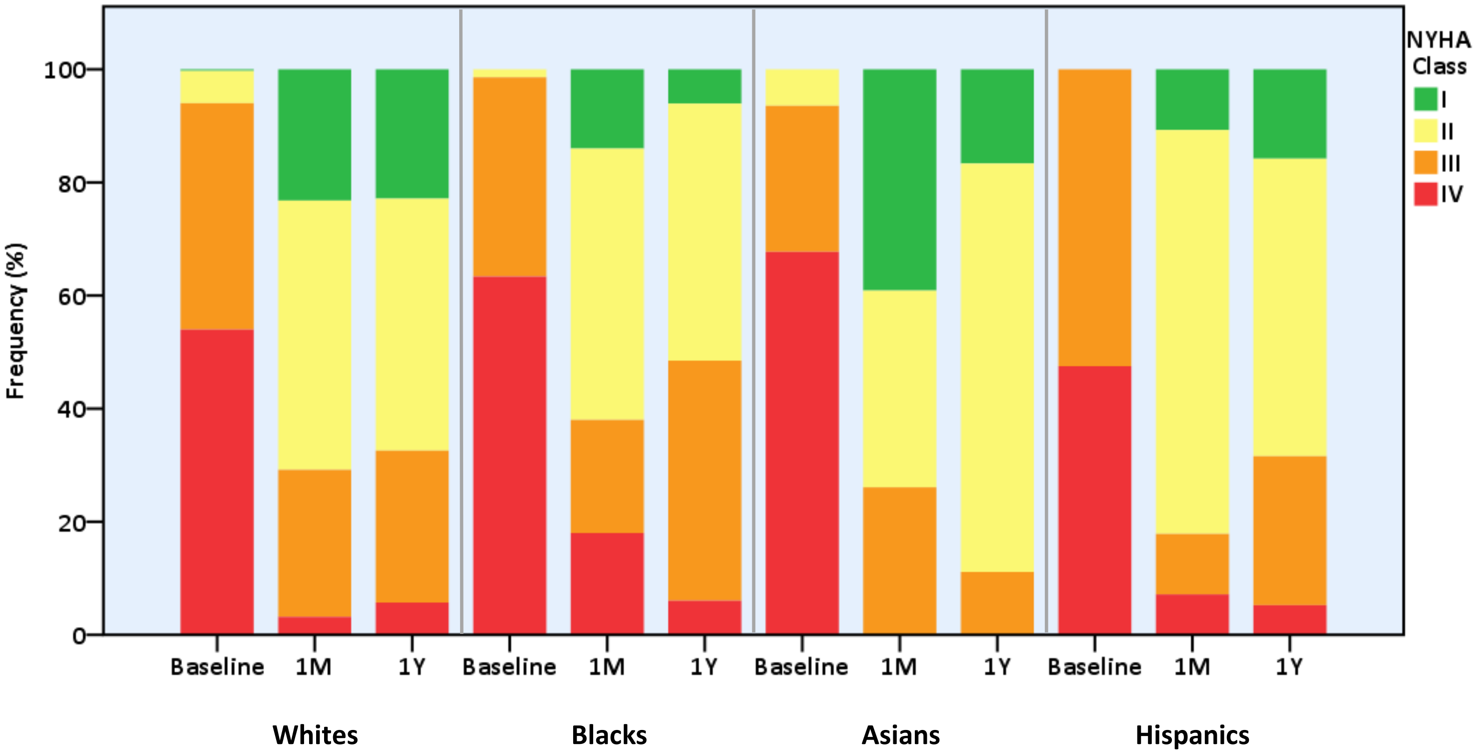

B Whites vs non-Whites

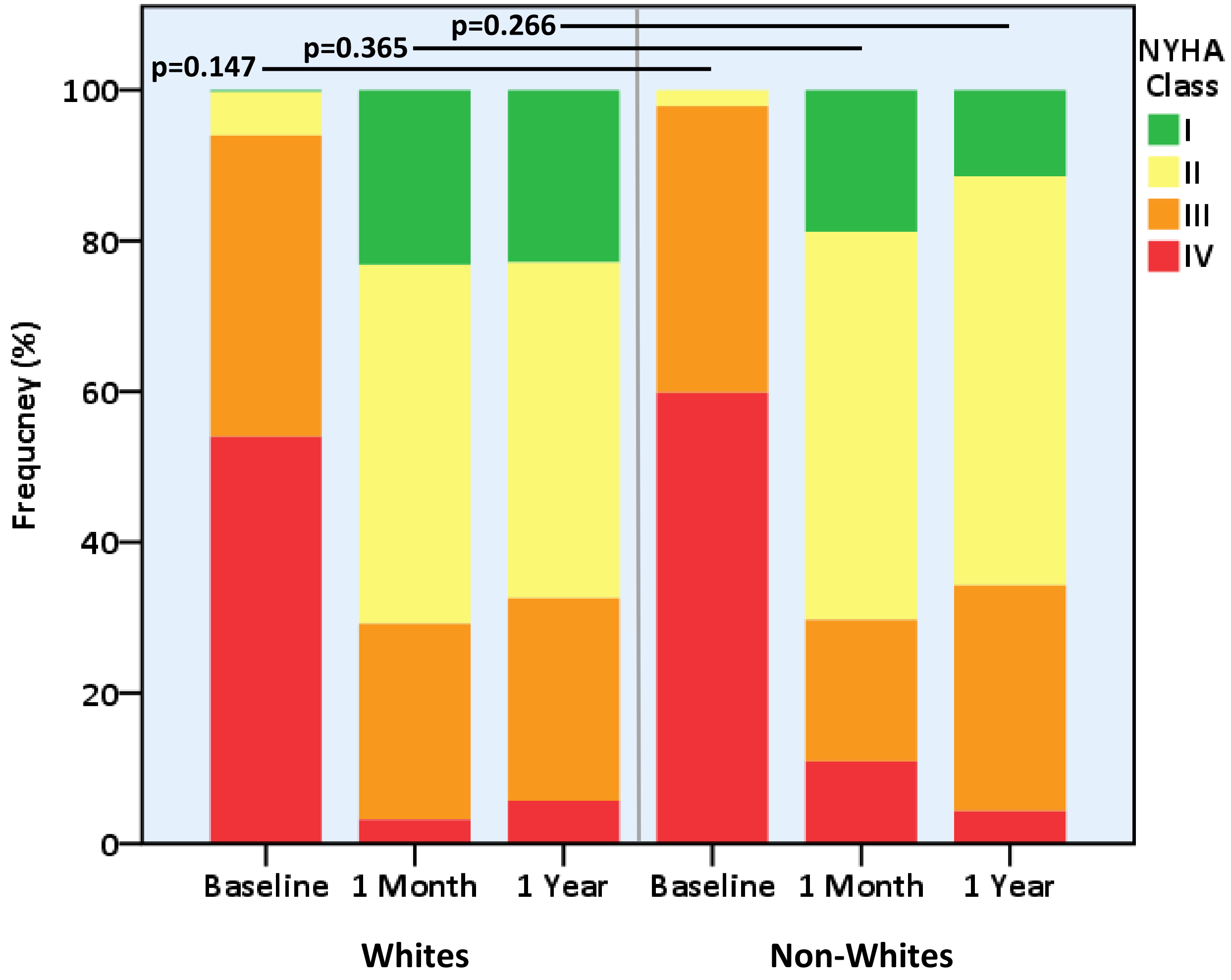

C Blacks vs non-Blacks

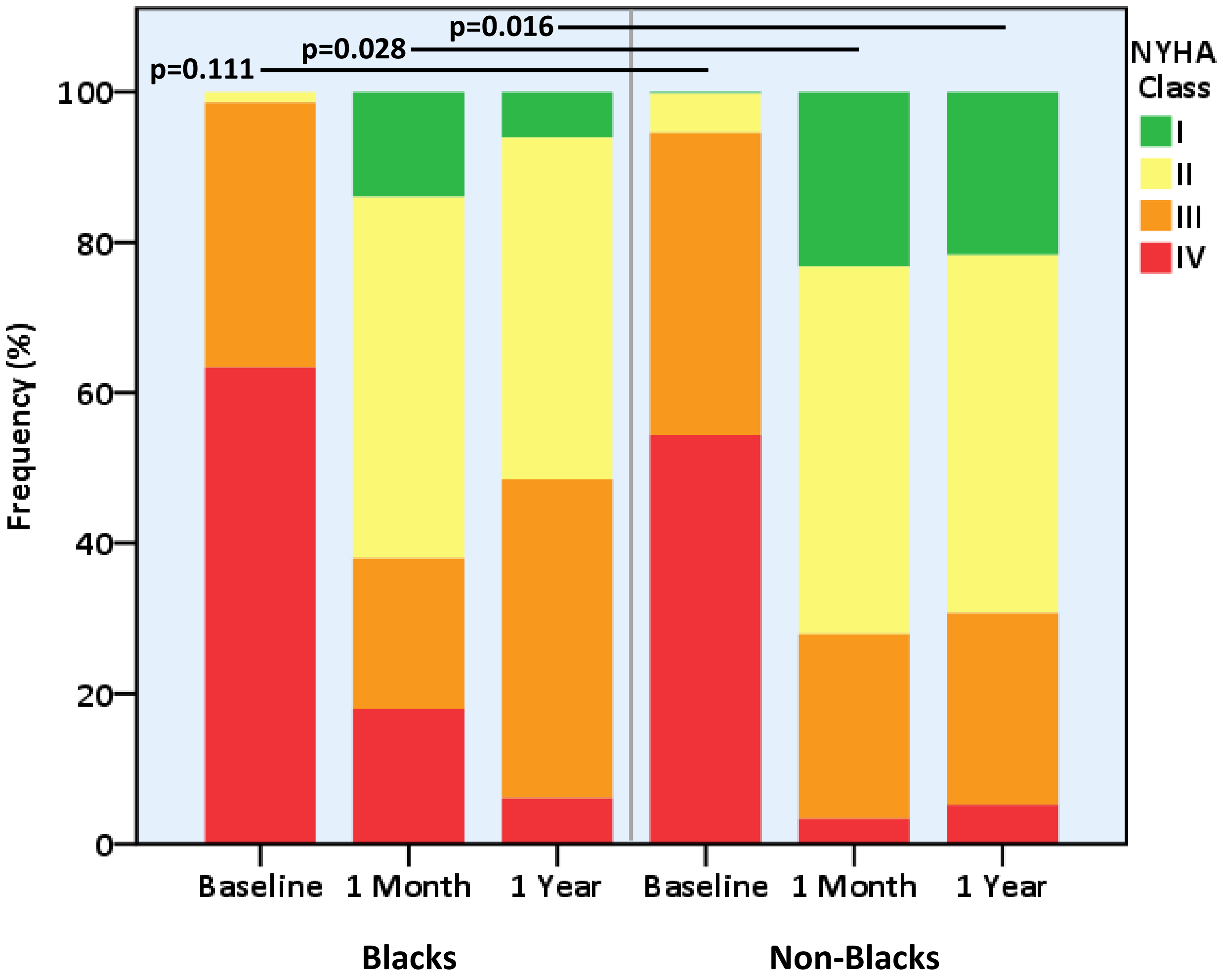

NYHA = New York Heart Association

Supplemental Figure 7. Mitral Regurgitation Grade at Baseline and Following Transcatheter Edge-to-Edge Repair for Functional Mitral Regurgitation According to Race

A All Races

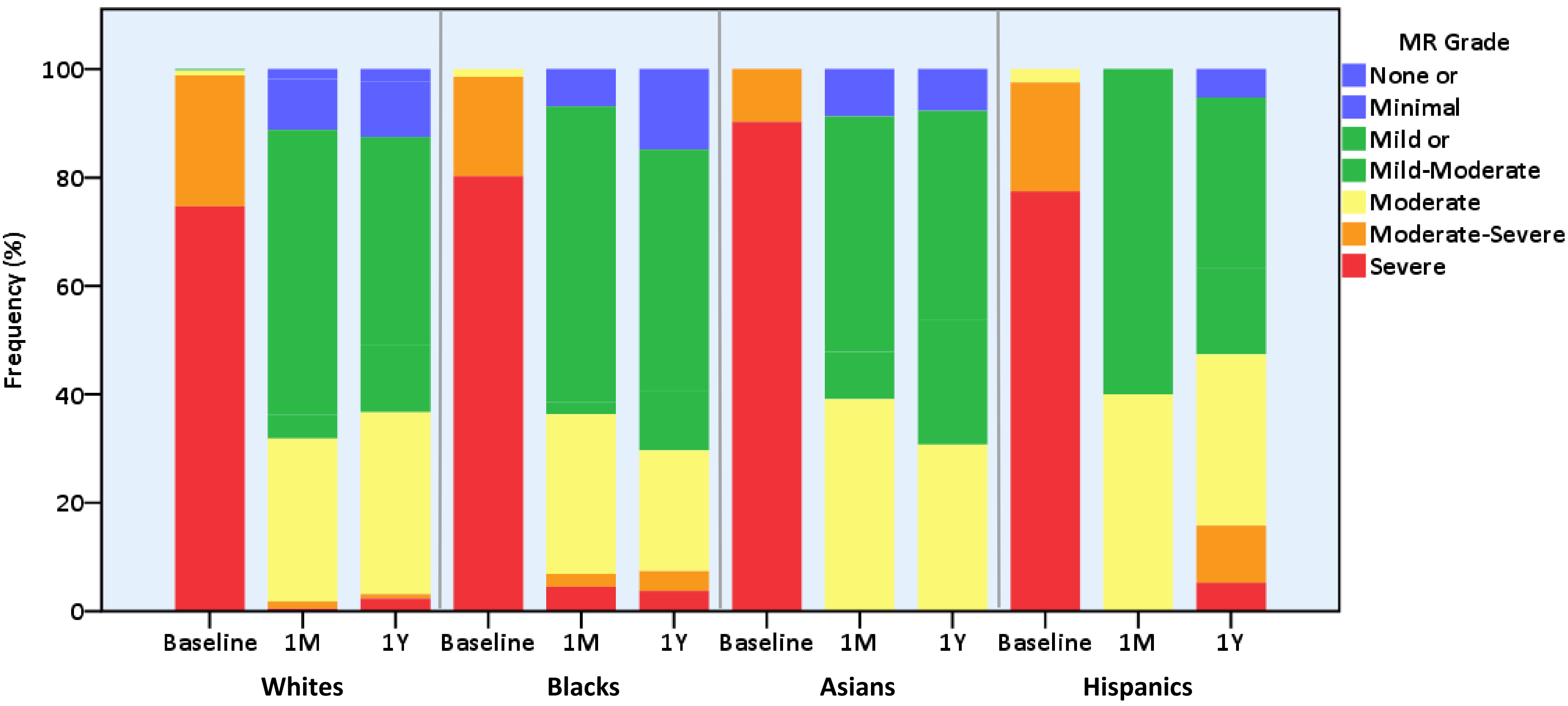

B Whites vs non-Whites

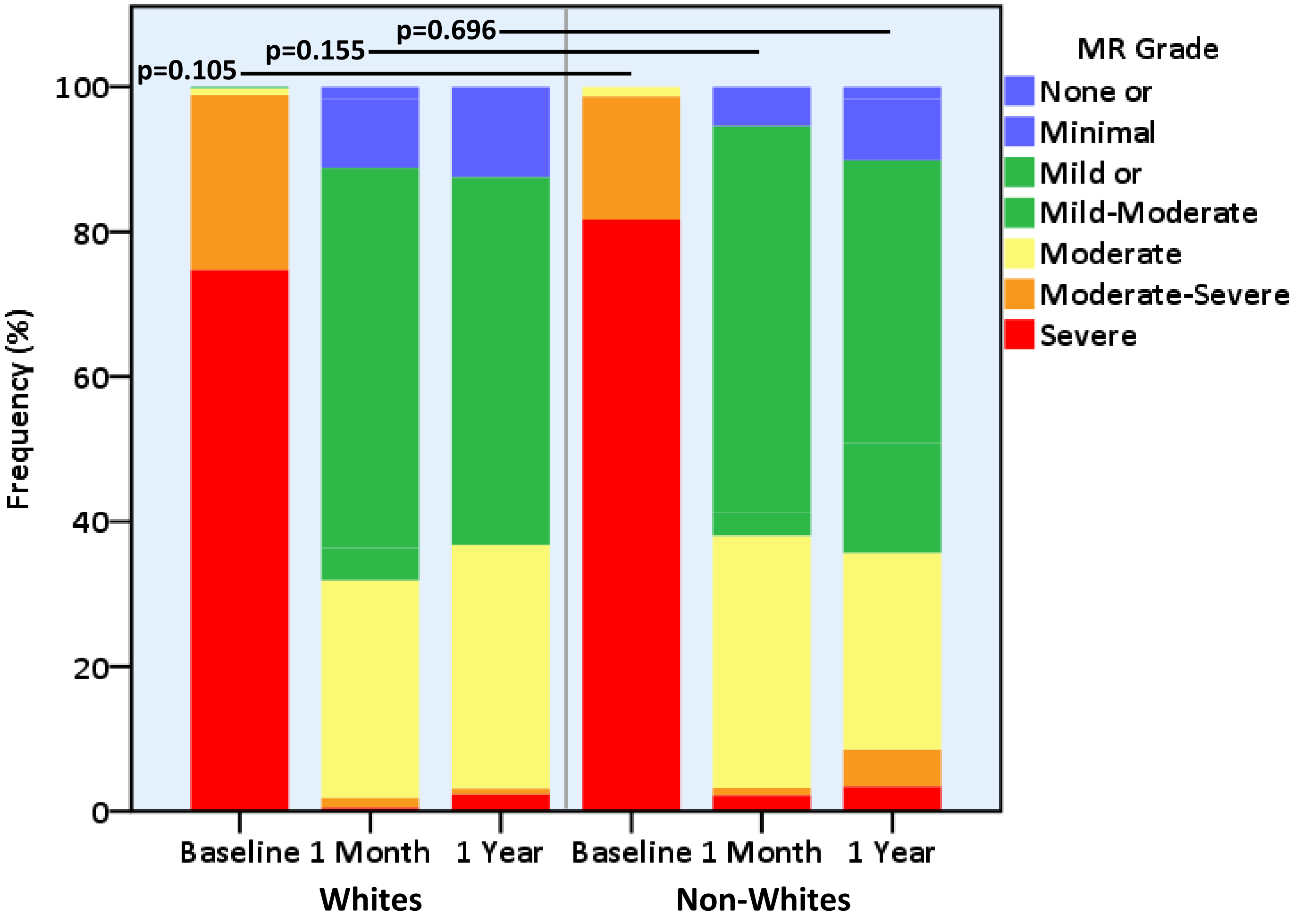

C Blacks vs non-Blacks

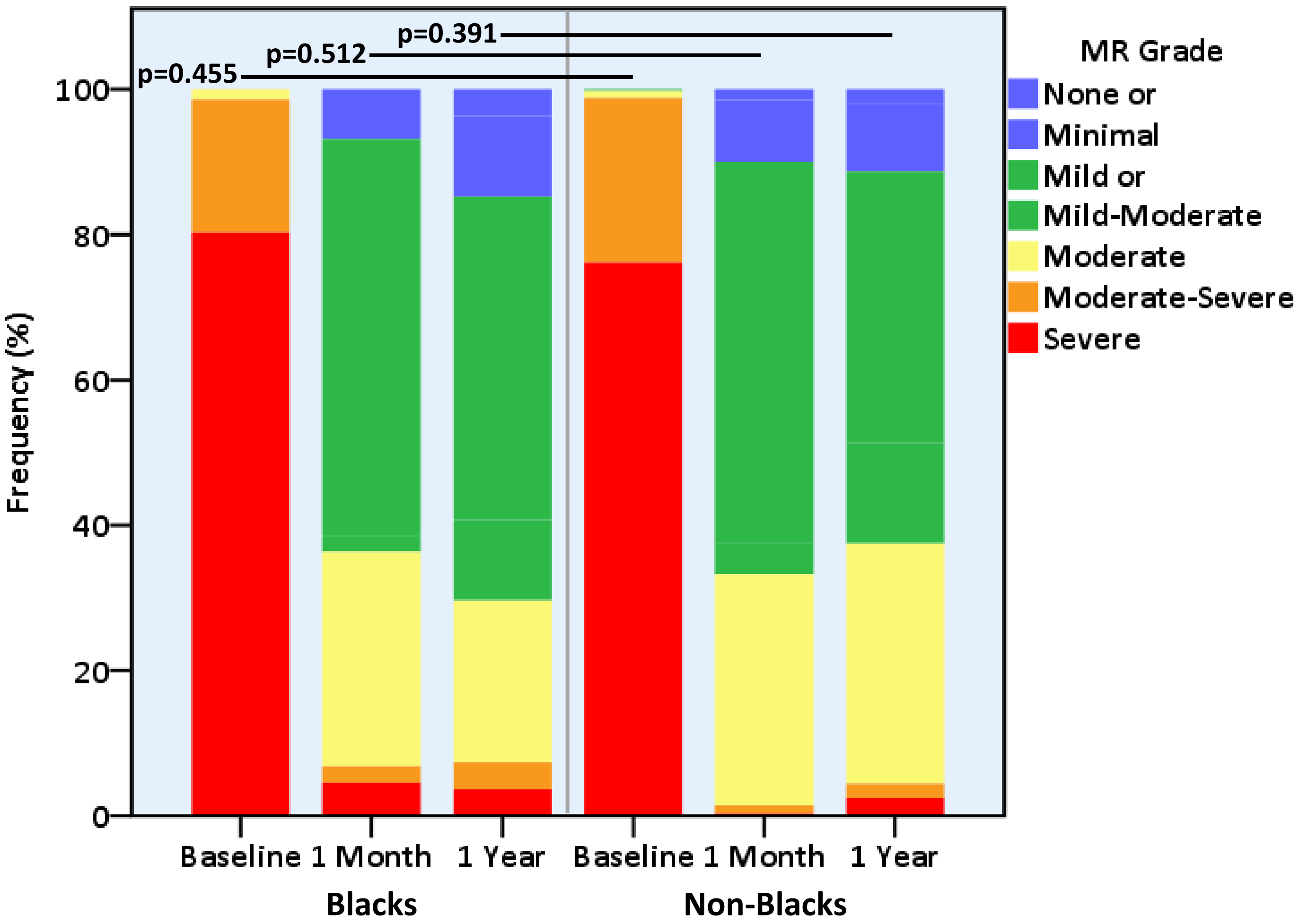

MR = mitral regurgitation

Supplemental Table 7. Baseline Clinical Characteristics of Patients with Primary Mitral Regurgitation According to Race

					P-Value				
	**Whites (N=399)**	**Blacks (N=17)**	**Asians (N=37)**	**Hispanics (N=17)**	**Whites vs Non-Whites**	**Blacks vs Non-Blacks**	**Asians vs Non-Asians**	**Hispanics vs Non-Hispanics**	**Whites vs Blacks**
**Demographic Details**									
Age (years)	83 (76-88)	78 (61-87)	81 (74-86)	82 (71-88)	**0.040**	0.058	0.205	0.831	0.052
Sex Male	249 (62.4)	11 (64.7)	21 (56.8)	10 (58.8)	0.603	0.809	0.501	0.789	0.848
Insurance									
None	1 (0.3)	0 (0.0)	0 (0.0)	0 (0.0)	0.672	0.846	0.769	0.846	0.836
Low-Income	23 (5.8)	1 (5.9)	8 (21.6)	6 (35.3)	**<0.001**	0.731	**0.006**	**0.001**	0.988
Regular / Full	373 (94.0)	16 (94.1)	29 (78.4)	11 (64.7)	**<0.001**	0.710	**0.007**	**0.001**	0.978
Median Yearly Household Income* (K USD)	87.0 (65.7-105.8)	58.8 (53.7-71.4)	79.8 (59.8-102.6)	84.8 (65.7-100.5)	**0.011**	**0.001**	0.262	0.898	**0.001**
Percentage of Adults with Academic Degree*	47.2 (32.2-63.7)	19.0 (11.9-43.3)	40.0 (31.1-48.1)	39.4 (19.0-58.9)	**<0.001**	**0.001**	0.062	0.222	**0.001**
**Comorbidities**									
Obesity (Body Mass Index ≥30kg/m^2)	57 (14.3)	3 (17.6)	3 (8.1)	2 (11.8)	0.497	0.717	0.294	0.802	0.722
Diabetes Mellitus	65 (16.4)	4 (23.5)	14 (37.8)	6 (35.3)	**0.001**	0.544	**0.002**	0.109	0.502
Hypertension	326 (81.7)	15 (93.8)	27 (73.0)	14 (82.4)	0.735	0.326	0.167	0.922	0.325
Smoking History	14 (3.5)	0 (0.0)	0 (0.0)	0 (0.0)	0.243	0.460	0.614	0.460	0.431
Previous MI, PCI, or CABG	125 (31.3)	2 (11.8)	12 (32.4)	8 (47.1)	0.954	0.077	0.874	0.153	0.086
Prior Stroke or Transient Ischemic Attack (TIA)	47 (11.8)	1 (5.9)	5 (13.5)	4 (23.5)	0.584	0.707	0.793	0.139	0.706
Peripheral Arterial Disease (PAD)	31 (7.8)	2 (11.8)	2 (5.4)	1 (5.9)	0.828	0.381	0.589	0.777	0.636
Atrial Fibrillation / Flutter	224 (56.1)	6 (35.3)	15 (40.5)	9 (52.9)	**0.031**	0.114	0.086	0.926	0.090
Chronic Obstructive Pulmonary Disease (COPD)	53 (13.3)	1 (5.9)	2 (5.4)	1 (5.9)	0.069	0.707	0.292	0.707	0.710

Anemia*	225 (56.4)	12 (70.6)	22 (59.5)	11 (64.7)	0.272	0.264	0.796	0.537	0.247
Stage ≥III Chronic Kidney Disease	290 (74.0)	13 (76.5)	33 (89.2)	13 (81.3)	0.065	0.928	**0.044**	0.771	0.819
**Heart Failure Indices**									
New York Heart Association (NYHA) Class									
II	33 (8.3)	2 (11.8)	2 (5.4)	1 (5.9)	0.726	0.639	0.757	0.734	0.645
III	170 (42.6)	7 (41.2)	16 (43.2)	9 (52.9)	0.699	0.878	0.973	0.398	0.907
IV	196 (49.1)	8 (47.1)	19 (51.4)	7 (41.2)	0.848	0.875	0.759	0.514	0.868
Kansas City Cardiomyopathy Questionnaire 12 Score	41.7 (20.4-63.8)	38.5 (12.5-53.9)	42.7 (19.5-77.6)	37.5 (6.3-75.0)	0.666	0.481	0.692	0.511	0.457
6-Minute Walk Test Distance (m)	244 (122-335)	183 (122-335)	335 (152-366)	198 (110-349)	0.715	0.648	0.222	0.585	0.670
Serum B-type Natriuretic Peptide (pg/mL)	328 (169-635)	752 (159-1,440)	359 (224-689)	306 (230-486)	0.103	0.067	0.312	0.841	0.059
**Risk Status**									
STS Score for Mitral Valve Repair	5.0 (2.7-7.7)	5.7 (2.2-8.6)	7.0 (4.1-10.1)	7.8 (2.5-13.4)	**0.008**	0.754	**0.010**	0.089	0.921
MitraScore	2 (3-4)	4 (3-4)	3 (2-4)	3 (2-4)	0.565	0.523	0.884	0.799	0.517
**Treatment**									
Medications									
Beta Blockers	248 (62.2)	12 (70.6)	20 (54.1)	10 (58.8)	0.632	0.443	0.319	0.804	0.482
Renin Angiotensin System (RAS) Inhibitors	187 (46.9)	7 (41.2)	18 (48.6)	8 (47.1)	0.952	0.635	0.815	0.983	0.645
Mineralocorticoid Receptor Antagonists (MRAs)	41 (10.3)	3 (17.6)	5 (13.5)	4 (23.5)	0.104	0.424	0.592	0.113	0.407
Loop Diuretics									
Frequency	271 (67.9)	16 (94.1)	29 (78.4)	12 (70.6)	**0.037**	**0.026**	0.268	0.942	**0.022**
Furosemide-Equivalent Dose (mg/day)	40 (20-60)	40 (25-70)	40 (20-40)	40 (20-80)	0.956	0.562	0.95	0.304	0.577
Anti-Arrhythmics	68 (17.1)	3 (17.6)	6 (16.2)	4 (23.5)	0.801	0.967	0.860	0.511	0.952
Hydralazine + Nitrates	5 (1.3)	0 (0.0)	0 (0.0)	0 (0.0)	0.350	0.670	0.520	0.660	0.650
Oral Anticoagulants	179 (44.9)	7 (41.2)	13 (35.1)	8 (47.1)	0.396	0.808	0.256	0.799	0.765

Cardiac Implantable Electronic Device (CIED)									
Total	77 (19.3)	6 (35.3)	3 (8.1)	4 (23.5)	0.845	0.110	0.075	0.546	0.121
Pacemaker	51 (12.8)	4 (23.5)	1 (2.7)	3 (17.6)	0.723	0.250	0.069	0.460	0.260
Implantable Cardioverter Defibrillator (ICD)	7 (1.8)	1 (5.9)	0 (0.0)	1 (5.9)	0.631	0.284	1.000	0.284	0.286
CRT/Defibrillator (CRT/D)	19 (4.8)	1 (5.9)	2 (5.4)	0 (0.0)	1.000	0.564	0.688	1.000	0.575

Data are presented as number (percentage) or median (interquartile range), where appropriate.

* Per zip code

+ Anemia was defined as a blood hemoglobin of <13mg/dL in men or <12mg/dL in women.

CABG = coronary bypass artery grafting; CRT = cardiac resynchronization therapy; GFR = glomerular filtration rate; MI = myocardial infarction; PCI = percutaneous coronary intervention; STS = Society of Thoracic Surgeons; USD = United States Dollars

Supplemental Table 8. Baseline Echocardiographic Data of Patients with Primary Mitral Regurgitation According to Race

					P-Value				
	**Whites (N=399)**	**Blacks (N=17)**	**Asians (N=37)**	**Hispanics (N=17)**	**Whites vs Non-Whites**	**Blacks vs Non-Blacks**	**Asians vs Non-Asians**	**Hispanics vs Non-Hispanics**	**Whites vs Blacks**
**Mitral Valve**									
Mitral Regurgitation Severity									
Moderate-Severe	67 (16.9)	1 (5.9)	2 (5.4)	4 (23.5)	0.136	0.494	0.071	0.325	0.328
Severe	328 (82.6)	16 (94.1)	35 (94.6)	13 (76.5)	0.114	0.330	0.063	0.498	0.327
Mitral Regurgitation PISA EROA (cm^2)	0.40 (0.29-0.52)	0.40 (0.26-0.86)	0.39 (0.28-0.42)	0.30 (0.22-0.49)	0.211	0.528	0.253	0.186	0.588
Mitral Regurgitation PISA RVol (mL)	54.5 (41.4-76.6)	43.7 (37.2-121.0)	53.8 (44.1-73.9)	45.2 (35.8-72.6)	0.418	0.931	0.633	0.373	0.944
Transmitral Mean Pressure Gradient (TMPG) (mmHg)	3 (2-4)	3 (2-4)	3 (2-4)	4 (2-4)	0.133	0.879	0.095	0.511	0.961
≥Moderate Mitral Annulus Calcification (MAC)	49 (12.3)	1 (5.9)	4 (10.8)	4 (23.5)	0.926	0.708	0.768	0.145	0.706
**Left Heart**									
Left Ventricular Ejection Fraction (LVEF) (%)	62 (55-67)	56 (41-63)	65 (58-70)	62 (51-73)	0.960	**0.025**	0.216	0.576	**0.029**
Left Ventricular End-Systolic Diameter (LVESD) (cm)	3.2 (2.8-3.9)	3.4 (3.0-4.2)	3.2 (2.7-3.8)	3.3 (2.6-4.4)	0.699	0.113	0.601	0.916	0.119
Left Ventricular Mass Index, ASE Formula (gr/m^2)	116.5 (92.5-140.8)	115.2 (98.6-146.6)	133.4 (106.2-156.7)	120.9 (110.6-155.7)	**0.016**	0.962	**0.033**	0.132	0.800
Left Atrial Volume Index (LAVi) (cm^3/m^2)	59.0 (43.0-76.9)	53.4 (45.2-70.5)	62.0 (46.4-74.8)	62.0 (52.1-82.8)	0.709	0.392	0.690	0.316	0.441
**Right Heart**									
≥Moderate Right Ventricular Dysfunction	44 (12.4)	4 (26.7)	4 (11.4)	0 (0.0)	0.917	0.100	0.867	0.246	0.115
≥Moderate-Severe Tricuspid Regurgitation	68 (17.1)	3 (17.6)	13 (36.1)	3 (17.6)	0.047	0.916	**0.005**	0.916	1.000
**Right Ventricular (RV)-Pulmonary Arterial (PA) Coupling**									
Tricuspid Annular Plane Systolic Excursion (TAPSE) (mm)	18 (15-22)	20 (17-22)	19 (16-22)	16 (15-23)	0.550	0.260	0.796	0.678	0.257
Pulmonary Arterial Systolic Pressure (PASP) (mmHg)	43 (32-56)	54 (46-60)	46 (32-65)	47 (32-60)	**0.031**	**0.060**	0.192	0.709	**0.044**

TAPSE/PASP (mm/mmHg)	0.42 (0.29-0.61)	0.40 (0.29-0.55)	0.40 (0.31-0.54)	0.34 (0.22-0.43)	0.273	0.811	0.737	0.156	0.755

Data are presented as number (percentage) or median (interquartile range), where appropriate.

ASE = American Society of Echocardiography; EROA = effective regurgitant orifice area; PISA = proximal isovelocity surface area; Rvol = regurgitant volume

Supplemental Table 9. Procedural Details and Results Observed in Patients with Primary Mitral Regurgitation According to Race

					P-Value				
	**Whites (N=399)**	**Blacks (N=17)**	**Asians (N=37)**	**Hispanics (N=17)**	**Whites vs Non-Whites**	**Blacks vs Non-Blacks**	**Asians vs Non-Asians**	**Hispanics vs Non-Hispanics**	**Whites vs Blacks**
**Presentation to Procedure**									
Acute Decompensated Heart Failure	35 (8.8)	2 (11.8)	6 (16.2)	4 (23.5)	**0.035**	0.683	0.246	0.079	0.656
Cardiogenic Shock	4 (1.0)	0 (0.0)	1 (2.7)	0 (0.0)	0.561	1.000	0.338	1.000	1.000
Medical and/or Mechanical Hemodynamic Support	10 (2.5)	1 (5.9)	4 (10.8)	0 (0.0)	0.060	0.429	**0.024**	1.000	0.372
Urgent Procedure	43 (10.8)	3 (17.6)	9 (24.3)	0 (0.0)	0.139	0.435	**0.028**	0.241	0.418
**Procedural Aspects**									
Number of Clips Deployed									
0 (Aborted / Not Deployed)	8 (2.0)	0 (0.0)	1 (2.7)	0 (0.0)	1.000	1.000	0.525	1.000	1.000
1	183 (45.9)	3 (17.6)	16 (43.2)	13 (76.5)	0.901	**0.018**	0.750	**0.010**	**0.022**
2	156 (39.1)	10 (58.8)	16 (43.2)	4 (23.5)	0.616	0.098	0.634	0.168	0.104
≥2	208 (52.1)	14 (82.4)	20 (54.1)	4 (23.5)	0.829	**0.012**	0.828	**0.015**	**0.014**
≥3	52 (13.0)	4 (23.5)	4 (10.8)	0 (0.0)	0.681	0.253	0.710	0.147	0.265
Median	2 (1-2)	2 (2-3)	2 (1-2)	1 (1-2)	0.953	**0.011**	0.995	**0.016**	**0.016**
Device Generation									
1	157 (39.3)	5 (29.4)	14 (37.8)	12 (70.6)	0.494	0.364	0.780	**0.009**	0.411
2	114 (28.6)	7 (41.2)	13 (35.1)	5 (29.4)	0.259	0.286	0.440	0.988	0.280
3	88 (22.1)	4 (23.5)	5 (13.5)	0 (0.0)	0.072	0.762	0.265	**0.030**	1.000
4	40 (10.0)	1 (5.9)	5 (13.5)	0 (0.0)	0.681	0.581	0.391	0.394	0.575
Intervention Site									

A1P1	18 (4.5)	0 (0.0)	4 (10.8)	0 (0.0)	0.759	0.352	0.085	1.000	0.371
A2P2	370 (92.7)	16 (94.1)	30 (81.1)	17 (100.0)	0.249	0.756	**0.019**	0.384	0.829
A3P3	28 (7.0)	2 (11.8)	6 (16.2)	0 (0.0)	0.215	0.380	0.053	0.631	0.351
Total Duration (min)	110 (90-139)	136 (111-179)	110 (97-143)	98 (82-130)	0.361	**0.011**	0.747	0.204	**0.011**
Fluoroscopy Duration (min)	19 (13-26)	22 (17-33)	20 (16-30)	16 (11-21)	0.176	0.089	0.133	0.202	0.084
Conversion to Surgery	1 (0.3)	0 (0.0)	0 (0.0)	0 (0.0)	1.000	1.000	1.000	1.000	1.000
**Echocardiographic and Hemodynamic Effects**									
Mitral Regurgitation Severity ≤Mild									
Immediately after Clip Deployment	305 (76.4)	9 (52.9)	28 (75.7)	15 (88.2)	0.551	**0.038**	0.967	0.384	**0.041**
At Discharge	328 (83.5)	9 (52.9)	24 (66.7)	14 (87.5)	**0.003**	**0.006**	**0.020**	0.747	**0.004**
Transmitral Mean Pressure Gradient (mmHg)									
Immediately after Clip Deployment	3 (2-4)	3 (3-5)	3 (2-5)	4 (2-5)	0.118	0.296	0.429	0.422	0.256
At 1-Month	4 (3-5)	5 (3-6)	4 (3-5)	5 (3-6)	0.550	0.249	0.454	0.383	0.249
Pulmonary Venous Flow Pattern Normalization on ≥1 Side	258 (71.5)	10 (71.4)	27 (84.4)	10 (76.9)	0.191	0.919	0.121	0.724	0.997
Delta V wave (mmHg)	-7 (-18-[-2])	-17 (-32-[-4])	-14 (-27-0)	-1 (-28-7)	0.089	0.071	0.082	0.340	0.059
Delta Mean Left Atrial Pressure (LAP) (mmHg)	-2 (-7-1)	-6 (-9-[-1])	-5 (-13-1)	0 (-12-4)	0.051	0.076	0.117	0.812	0.055
Delta Mean Pulmonary Arterial Pressure (PAP) (mmHg)	0 (-5-3)	3 (-8-5)	-4 (-14-3)	2 (-6-9)	0.580	0.683	0.056	0.384	0.714
**Post-Procedural Course**									
Intensive Cardiac Unit (ICU) Stay Duration (hours)	14.4±115.5	2.7±7.8	25.2±92.0	11.2±28.1	0.886	0.647	0.547	0.893	0.677
Hospitalization Length (days)	1 (1-2)	1 (1-5)	1 (1-5)	1 (1-5)	0.077	0.250	0.153	0.859	0.209
Discharge Home	371 (93.7)	16 (94.1)	35 (97.2)	15 (88.2)	0.848	0.953	0.716	0.286	0.943
Blood Transfusion or Any 1-Month Non-Fatal Adverse Event*	39 (9.8)	2 (11.8)	6 (16.2)	2 (11.8)	0.305	0.925	0.113	0.925	0.679
**Medical Treatment at 1-Month**									
Beta Blockers	200 (61.2)	11 (73.3)	15 (50.0)	8 (53.3)	0.513	0.298	0.222	0.564	0.343

Renin Angiotensin System (RAS) Inhibitors	160 (49.5)	9 (60.0)	17 (58.6)	5 (33.3)	0.671	0.429	0.334	0.188	0.428
Mineralocorticoid Receptor Antagonists (MRAs)	35 (10.7)	2 (13.3)	3 (10.0)	3 (20.0)	0.551	0.677	0.840	0.226	0.671
Loop Diuretics	205 (62.7)	14 (93.3)	22 (73.3)	9 (60.0)	0.067	**0.018**	0.298	0.704	**0.016**
Anti-Arrhythmics	60 (18.6)	3 (20.0)	2 (6.9)	5 (33.3)	0.884	0.961	0.253	0.288	0.964
Hydralazine + Nitrates	4 (1.2)	0 (0.0)	0 (0.0)	0 (0.0)	0.381	0.677	0.554	0.687	0.656
Oral Anticoagulants	156 (47.7)	7 (46.7)	11 (36.7)	8 (53.3)	0.533	0.977	0.236	0.618	

Data are presented as number (percentage) or median (interquartile range), or mean±standard deviation, where appropriate.

* 1-month non-fatal adverse events included the following: tamponade, cardiac arrest, myocardial infarction, stroke, transient ischemic attack, MVARC bleeding, and vascular complications.

MVARC = mitral valve academy research consortium

Supplemental Table 10. Outcomes and Trends following Transcatheter Edge-to-Edge Repair for Primary Mitral Regurgitation According to Race

					**P-Value**				
	**Whites (N=399)**	**Blacks (N=17)**	**Asians (N=37)**	**Hispanics (N=17)**	**Whites vs Non-Whites**	**Blacks vs Non-Blacks**	**Asians vs Non-Asians**	**Hispanics vs Non-Hispanics**	**Whites vs Blacks**
**Primary Outcome**									
All-Cause Mortality or Heart Failure Hospitalizations at 1-Year	66 (16.5)	3 (17.6)	8 (21.6)	1 (5.9)	0.940	0.906	0.392	0.329	0.904
Event-Free Survival (days)	318±6	307±30	294±23	343±21	0.833	0.934	0.271	0.243	0.921
**Secondary Outcomes**									
Clinical									
All-Cause Mortality at 1-Year	37 (9.3)	1 (5.9)	5 (13.5)	1 (5.9)	0.876	0.616	0.374	0.616	0.635
Heart Failure Hospitalizations at 1-Year	38 (9.5)	3 (17.6)	5 (13.5)	1 (5.9)	0.415	0.236	0.400	0.564	0.229
New York Heart Association Class ≤II									
At 1-Month	271 (87.7)	15 (100.0)	18 (64.3)	13 (86.7)	0.087	0.239	**0.002**	0.973	0.232
At 1-Year	176 (88.0)	5 (83.3)	16 (100.0)	6 (66.7)	0.775	0.544	0.229	0.081	0.545
Echocardiographic									
Mitral Regurgitation Severity ≤Mild									
At 1-Month	202 (65.6)	6 (40.0)	14 (53.8)	10 (66.7)	0.085	0.051	0.276	0.809	**0.043**
At 1-Year	93 (56.0)	1 (20.0)	6 (40.0)	5 (71.4)	0.263	0.180	0.243	0.458	0.176
Mitral Regurgitation Severity ≤Moderate									
At 1-Month	277 (91.7)	12 (80.0)	25 (96.2)	13 (86.7)	0.603	0.131	0.714	0.379	0.137
At 1-Year	150 (90.4)	4 (80.0)	13 (86.7)	7 (100.0)	0.734	0.408	0.647	0.373	0.411

Left Ventricular Mass Index (gr/m^2)									
At 1-Month	109.8 (88.3-133.3)	115.0 (93.7-159.4)	114.6 (103.5-160.2)	111.8 (89.2-138.1)	0.082	0.597	0.121	0.539	0.524
At 1-Year	106.5 (88.3-126.3)	89.6 (82.0-137.2)	117.9 (89.4-133.6)	121.1 (116.6-151.6)	0.218	0.568	0.512	0.067	0.627
Combined Clinical and Echocardiographic									
New York Heart Association Class ≤II or Mitral Regurgitation Severity ≤Mild									
At 1-Month	294 (94.2)	15 (100.0)	23 (82.1)	14 (93.3)	0.240	0.611	**0.027**	0.977	0.339
At 1-Year	189 (94.0)	5 (83.3)	16 (100.0)	7 (87.5)	0.700	0.316	0.606	0.398	0.326
**Trends**									
Absolute Change in New York Heart Association Class									
At 1-Month	-1.5±0.8	-1.6±0.6	-1.3±0.8	-1.9±0.9	0.957	0.760	0.074	**0.046**	0.322
At 1-Year	-1.5±0.9	-1.0±0.9	-1.2±0.6	-1.4±0.9	0.994	0.130	0.121	0.798	0.149
P-Value for 1-Year vs Baseline	**<0.001**	**0.041**	**<0.001**	**0.001**	NA	NA	NA	NA	NA
Relative Change in Left Ventricular Mass Index (%)									
At 1-Month	-3.3 (-22.6-14.2)	-4.3 (-17.9-15.9)	-3.0 (-27.1-10.1)	-12.0 (-27.8-12.5)	0.386	0.841	0.485	0.386	0.908
At 1-Year	-2.6 (-19.9-15.9)	-0.3 (-19.8-27.1)	-21.6 (-30.9-[-11.8])	4.1 (-20.4-7.3)	0.073	0.802	**0.005**	0.699	0.916

Data are presented as number (percentage), median (interquartile range), or mean±standard deviation, where appropriate.

NA = not applicable

Supplemental Figure 8. One-Year Cumulative Incidence of the Combined Outcome of All-Cause Mortality or Heart Failure Hospitalizations Following Transcatheter Edge-to-Edge Repair for Primary Mitral Regurgitation According to Race

A All Races

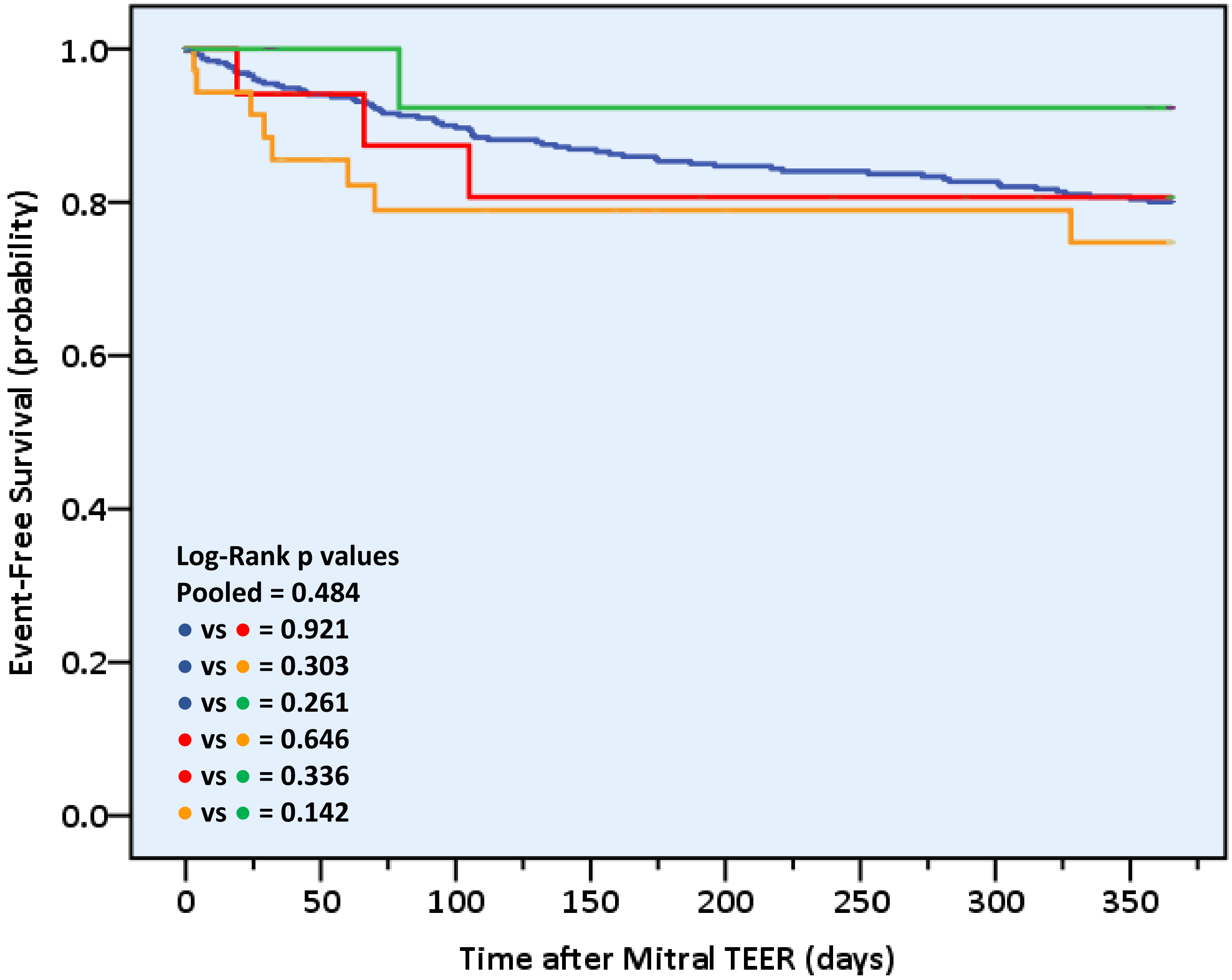

No. at Risk								
Whites	399	311	288	276	262	256	249	239
Blacks	17	14	13	12	11	10	9	8
Asians	37	27	24	23	19	19	19	18
Hispanics	17	13	12	12	12	12	12	12

B Whites vs non-Whites

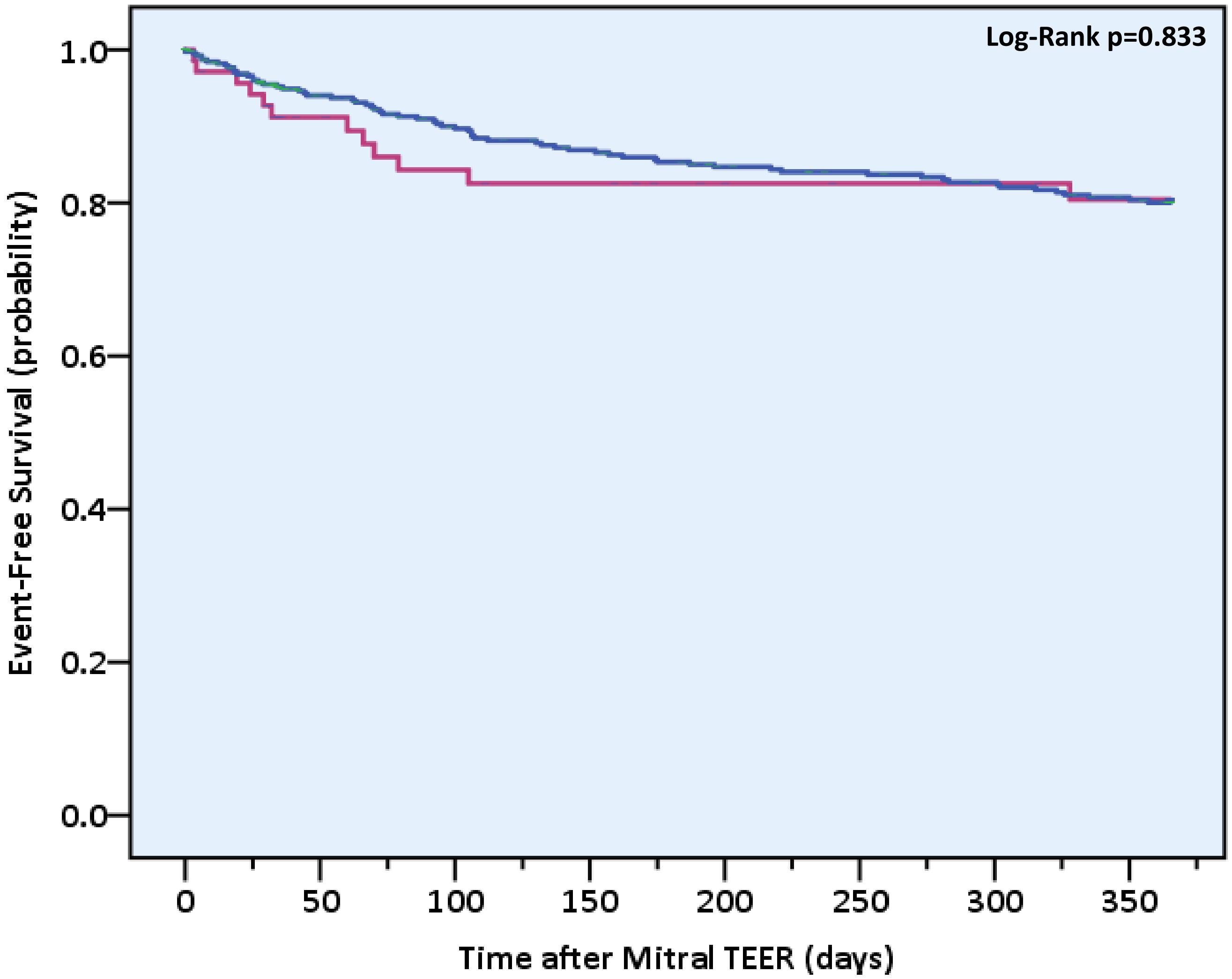

No. at Risk								
Whites	399	311	288	276	262	256	249	239
Non-Whites	71	54	49	47	42	41	40	38

C Blacks vs non-Blacks

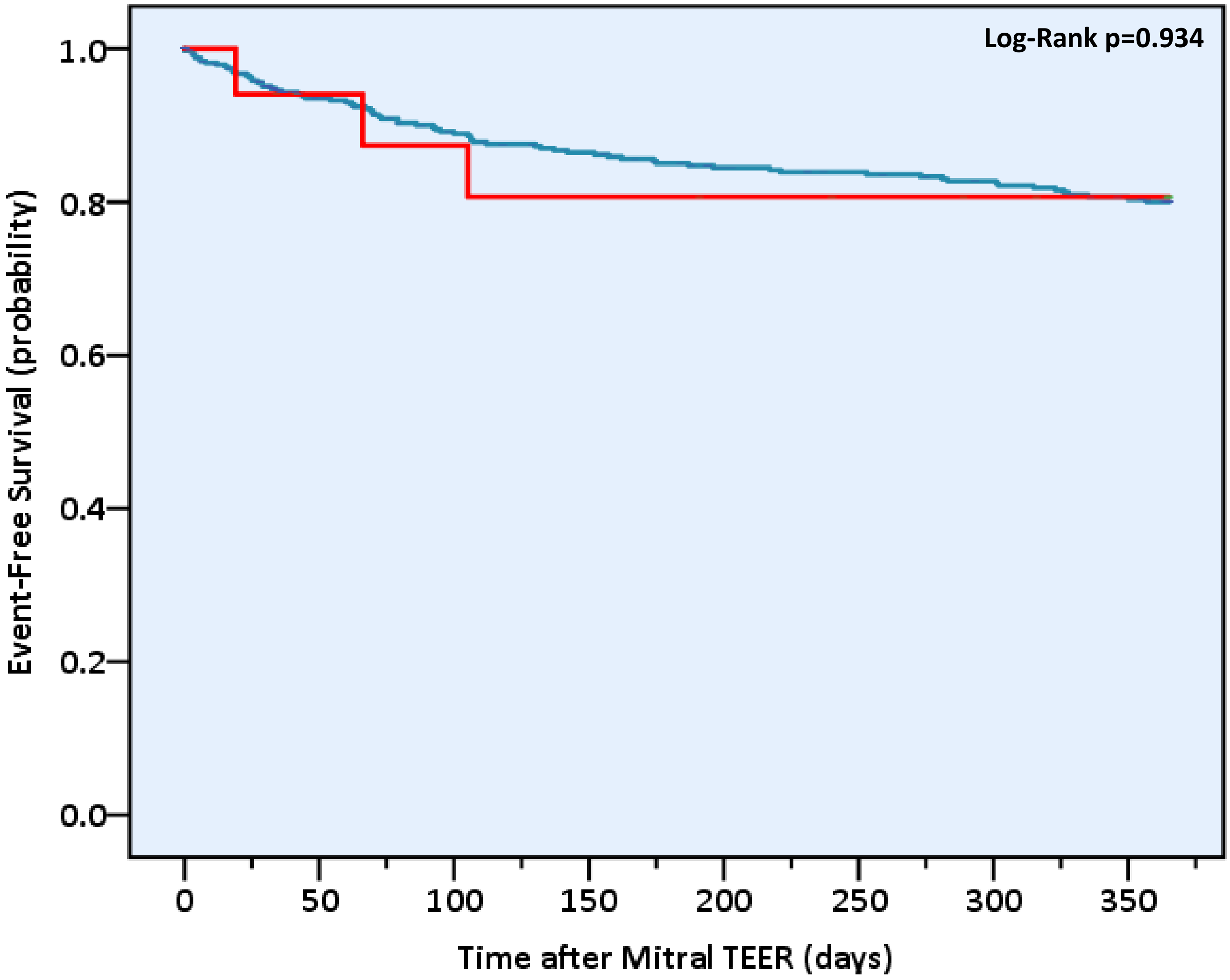

**No. at Risk**								
Non-Blacks	453	351	324	311	293	287	280	269
Blacks	17	14	13	12	11	10	9	8

D Asians vs non-Asians

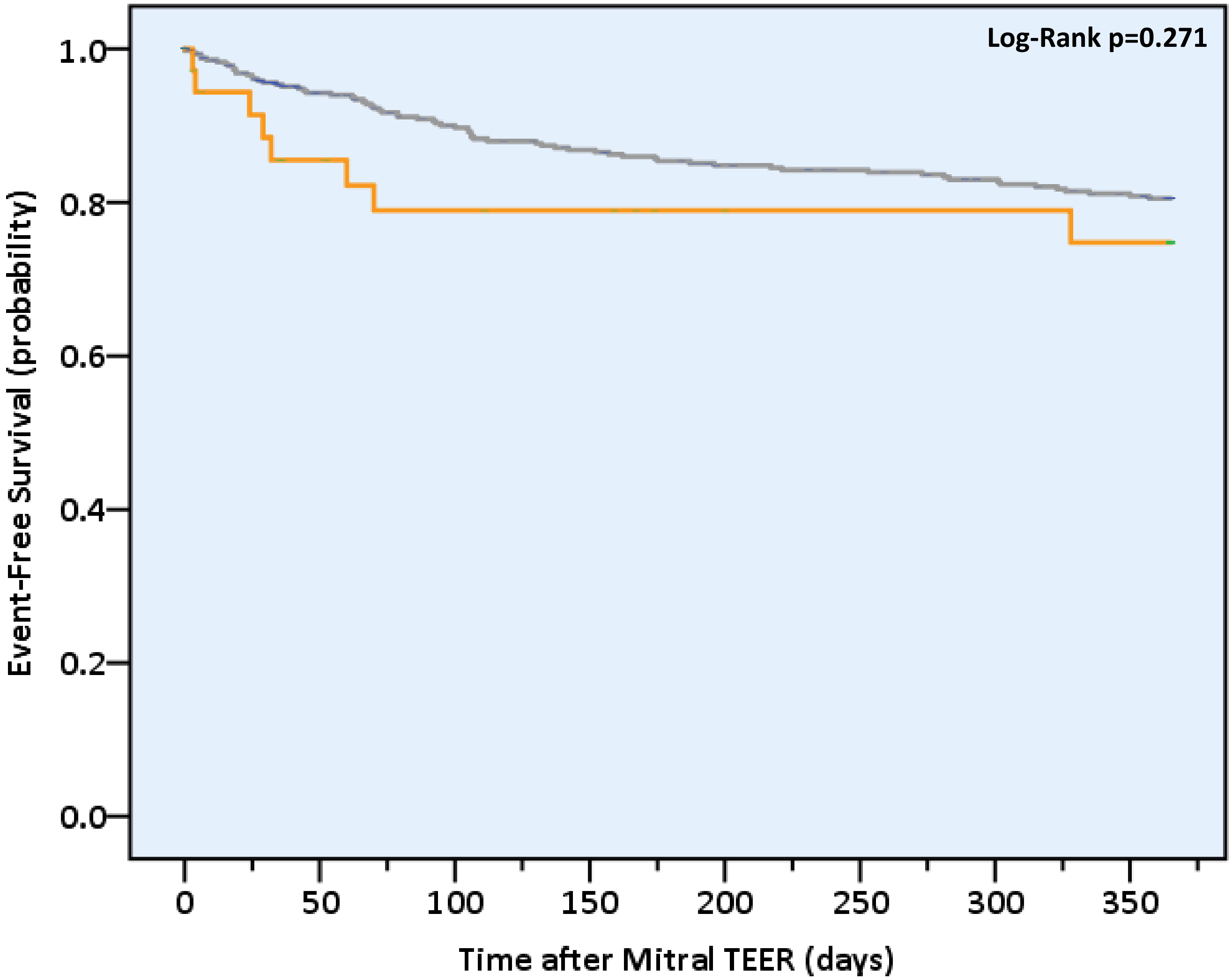

**No. at Risk**								
Non-Asians	433	338	313	300	285	278	270	259
Asians	37	27	24	23	19	19	19	18

E Hispanics vs non-Hispanics

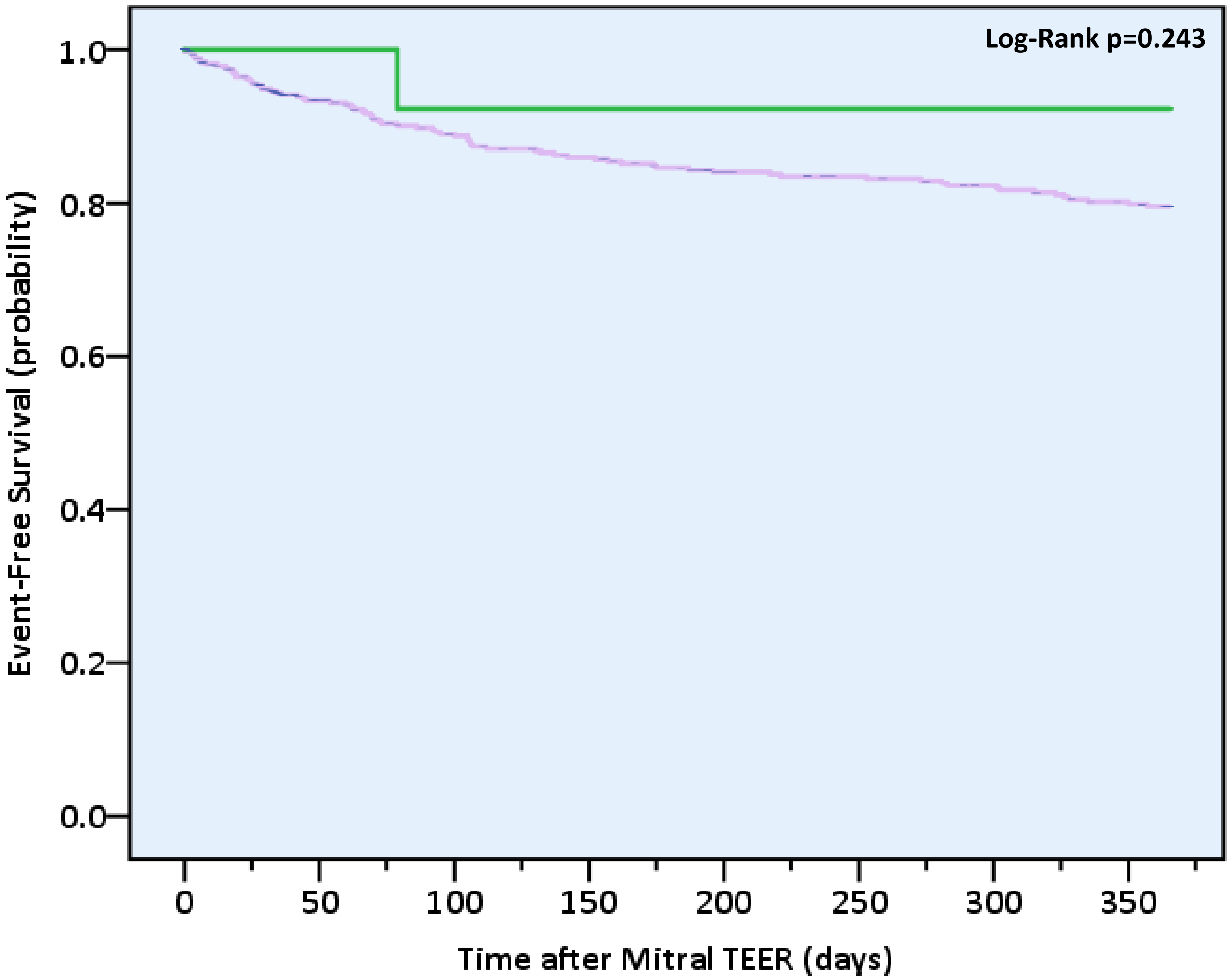

**No. at Risk**								
**Hispanics**	17	13	12	12	12	12	12	12
**Non-Hispanics**	453	352	325	311	292	285	277	265

TEER = transcatheter edge-to-edge repair

Supplemental Figure 9. One-Year Cumulative Incidence of All-Cause Mortality Following Transcatheter Edge-to-Edge Repair for Primary Mitral Regurgitation According to Race

A All Races

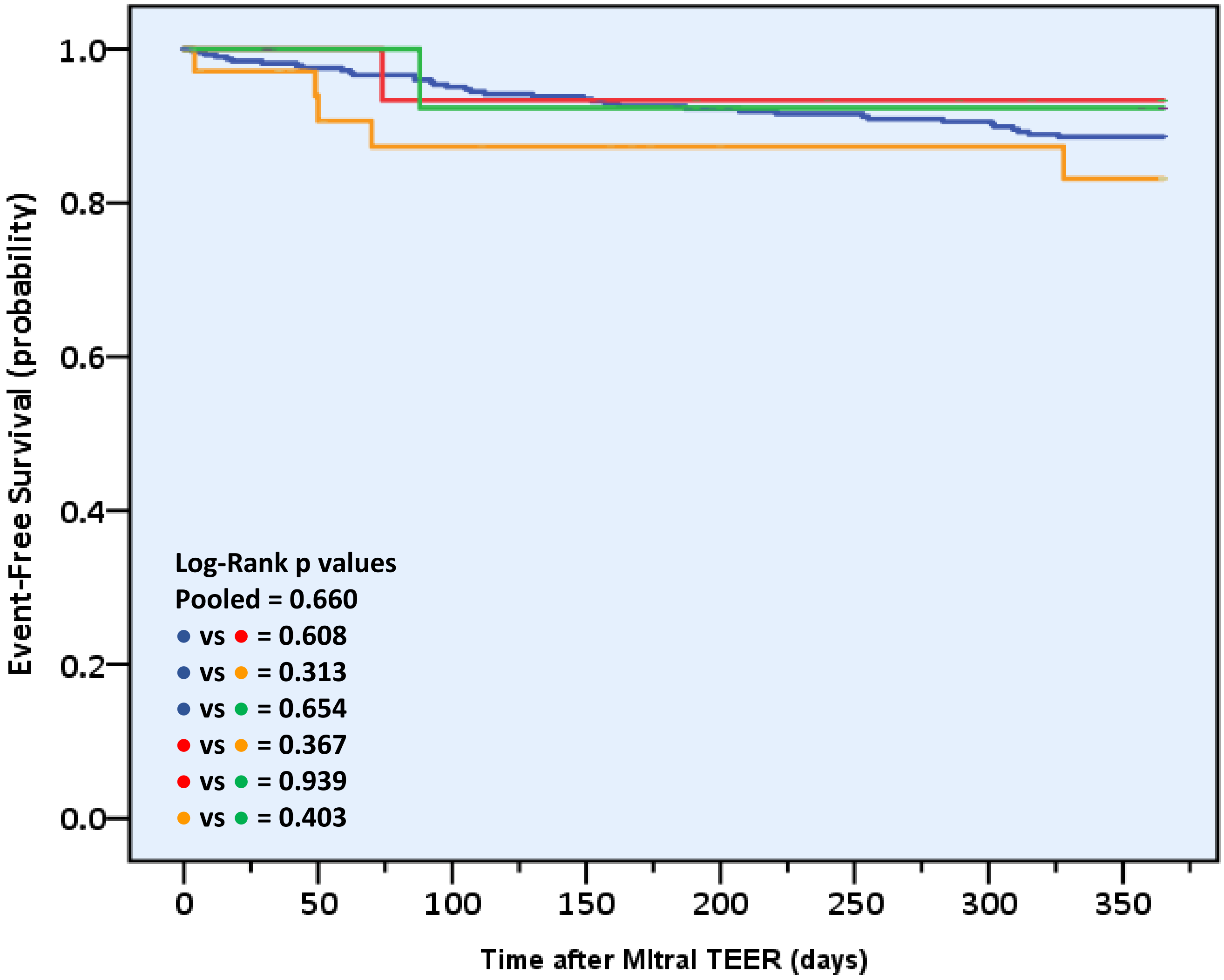

No. at Risk								
Whites	399	321	304	296	285	277	270	260
Blacks	17	15	14	14	13	12	11	10
Asians	37	28	26	25	21	21	21	20
Hispanics	17	13	12	12	12	12	12	12

B Whites vs non-Whites

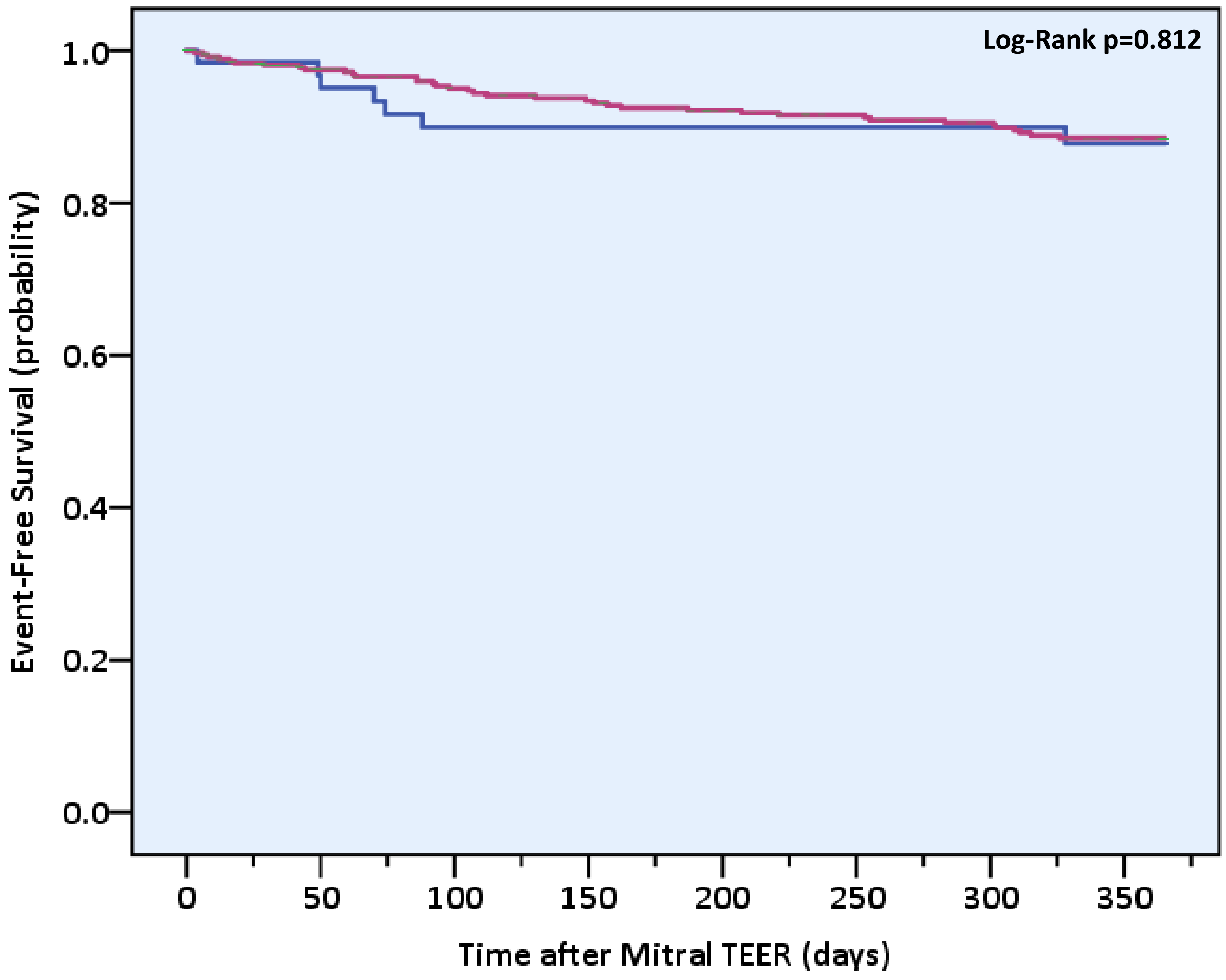

No. at Risk								
Whites	399	321	304	296	285	277	270	260
Non-Whites	71	56	52	51	46	45	44	42

C Blacks vs non-Blacks

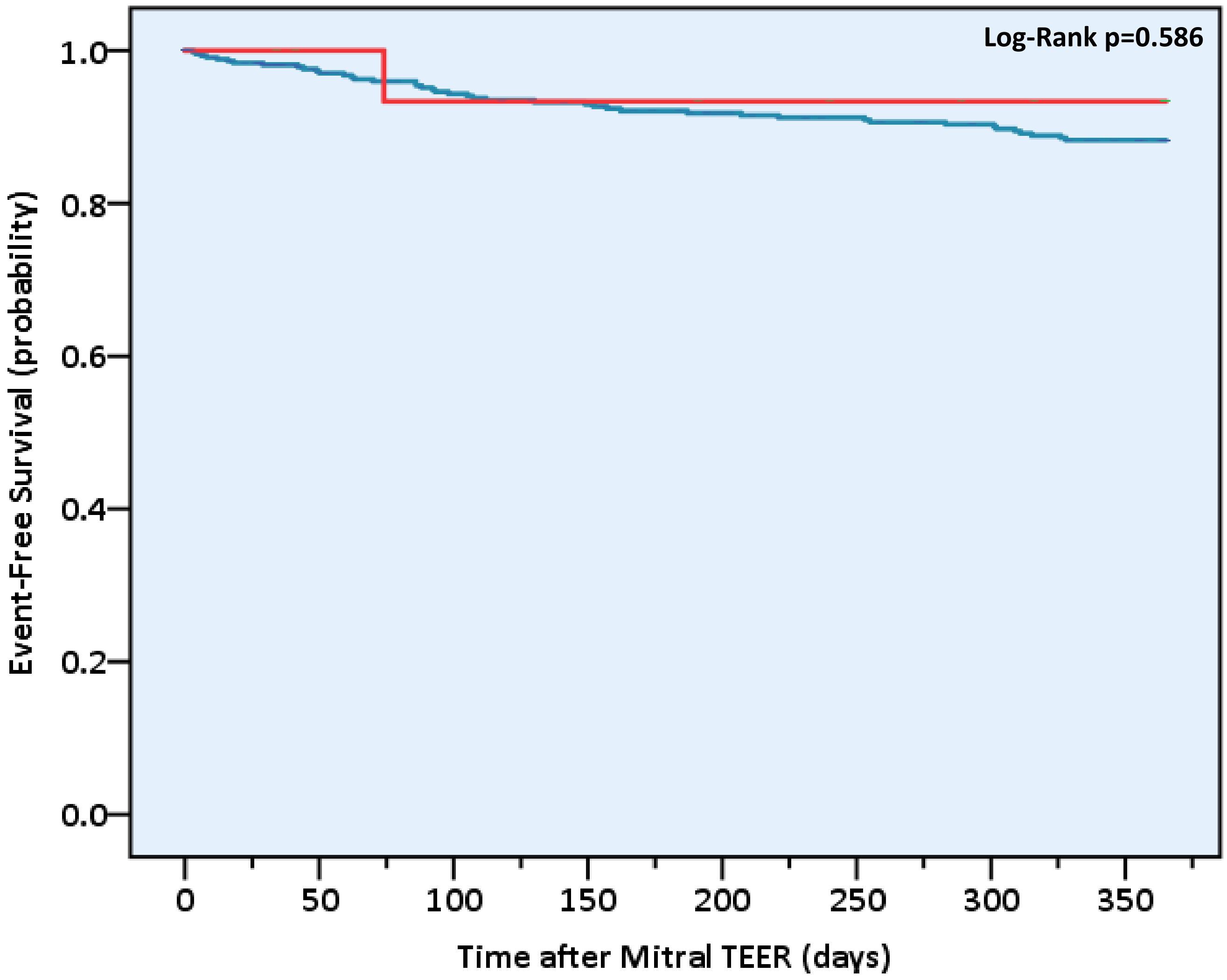

**No. at Risk**								
**Blacks**	17	15	14	14	13	12	11	10
**Non-Blacks**	453	362	342	333	318	310	303	292

TEER = transcatheter edge-to-edge repair

Supplemental Figure 10. One-Year Cumulative Incidence of Heart Failure Hospitalizations Following Transcatheter Edge-to-Edge Repair for Primary Mitral Regurgitation According to Race

A All Races

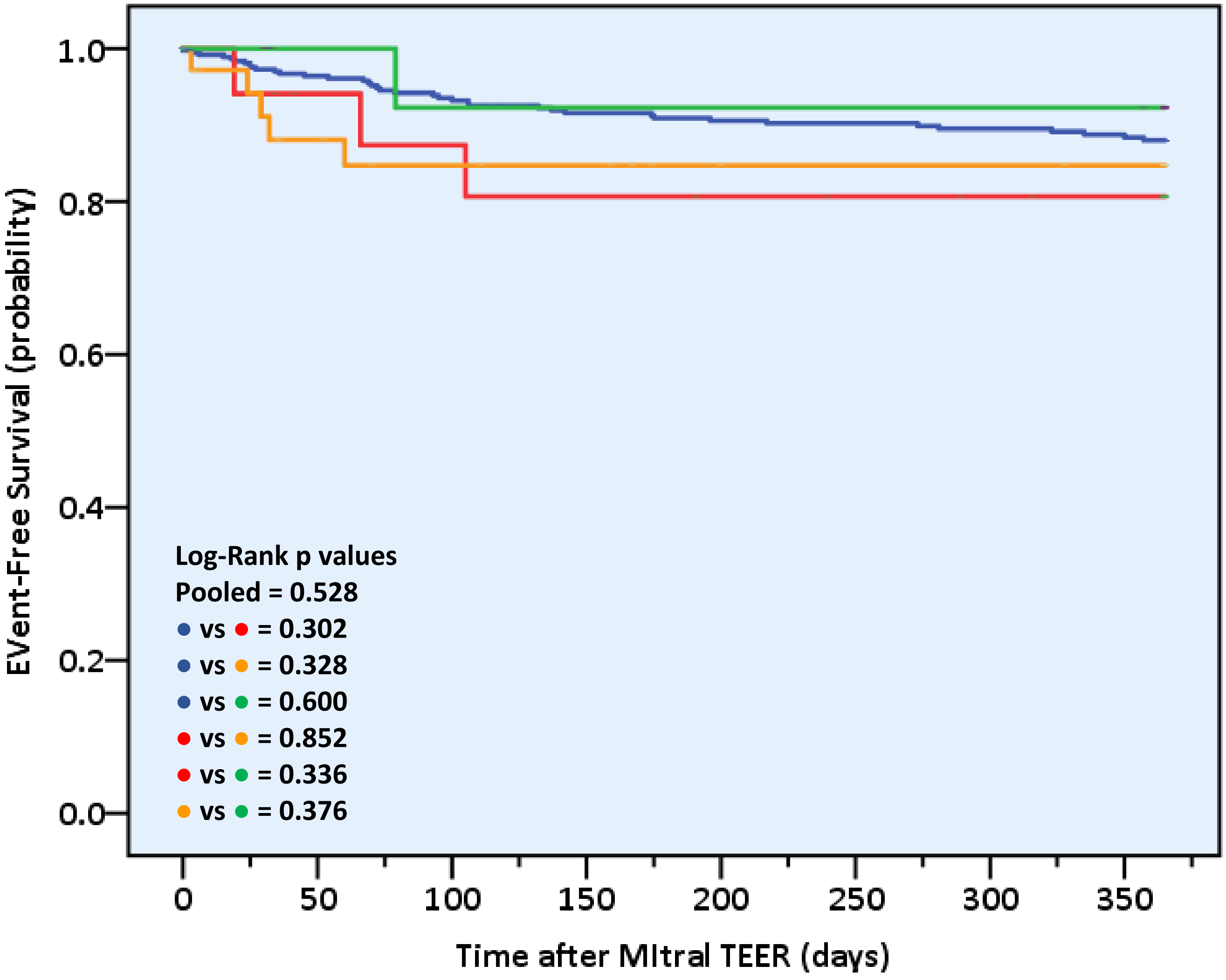

No. at Risk								
Whites	399	311	288	276	262	256	249	239
Blacks	17	14	13	12	11	10	9	8
Asians	37	27	24	23	19	19	19	18
Hispanics	17	13	12	12	12	12	12	12

B Whites vs non-Whites

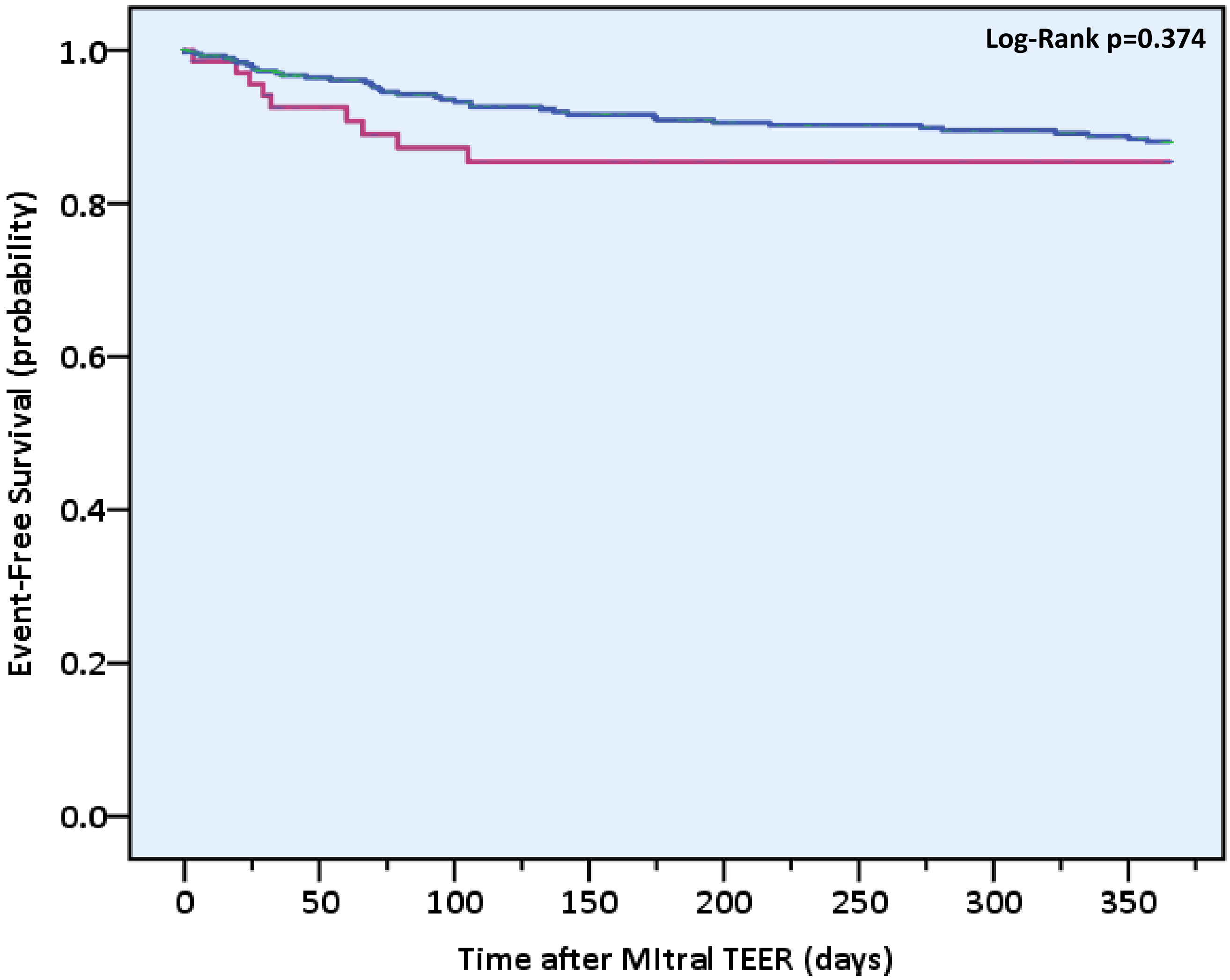

**No. at Risk**								
**Whites**	399	311	288	276	262	256	249	239
**Non-Whites**	71	54	49	47	42	41	40	38

C Blacks vs non-Blacks

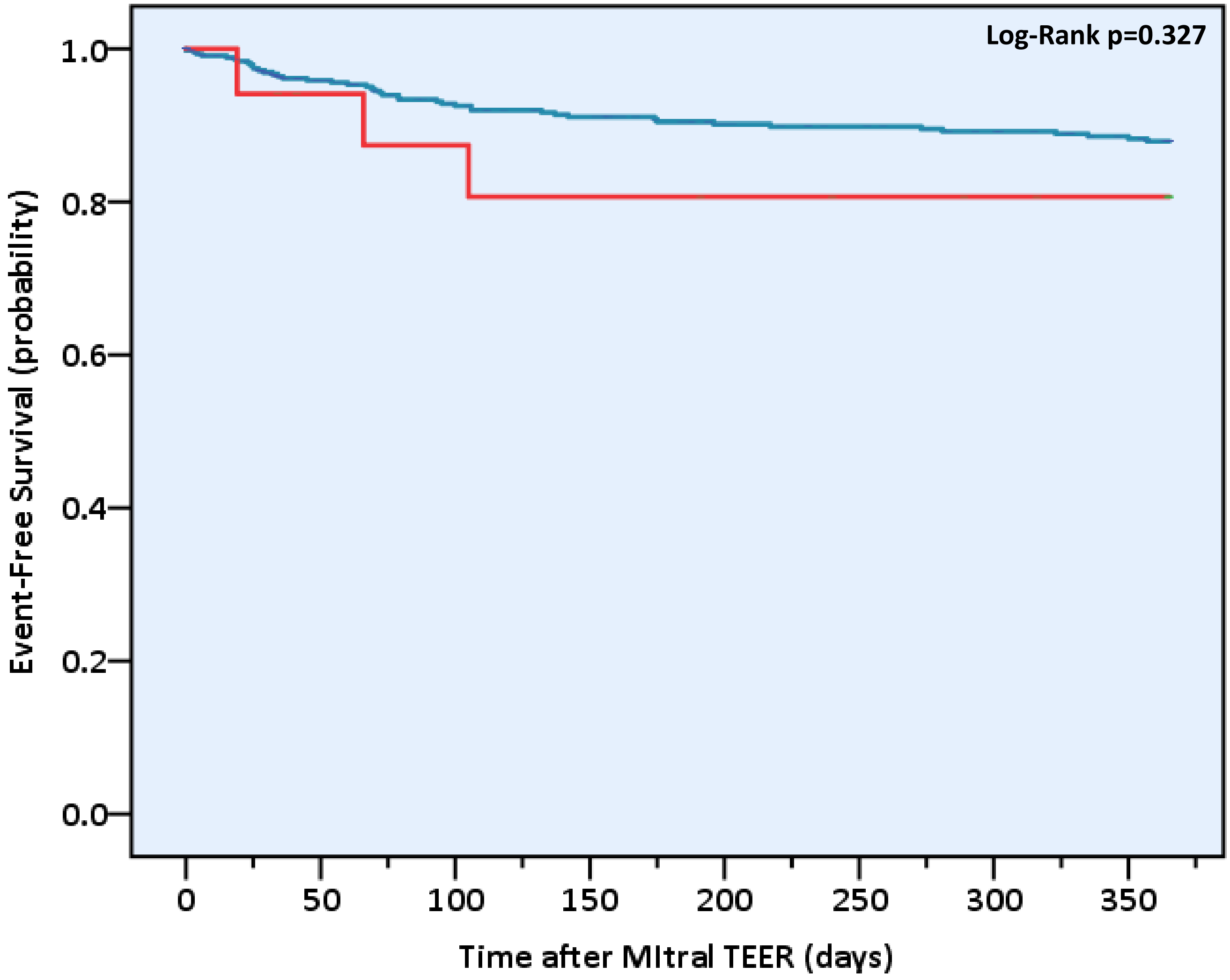

**No. at Risk**								
Non-Blacks	453	351	324	311	293	287	280	269
Blacks	17	14	13	12	11	10	9	8

TEER = transcatheter edge-to-edge repair

Supplemental Figure 11. Functional Status at Baseline and Following Transcatheter Edge-to-Edge Repair for Primary Mitral Regurgitation According to Race

A All Races

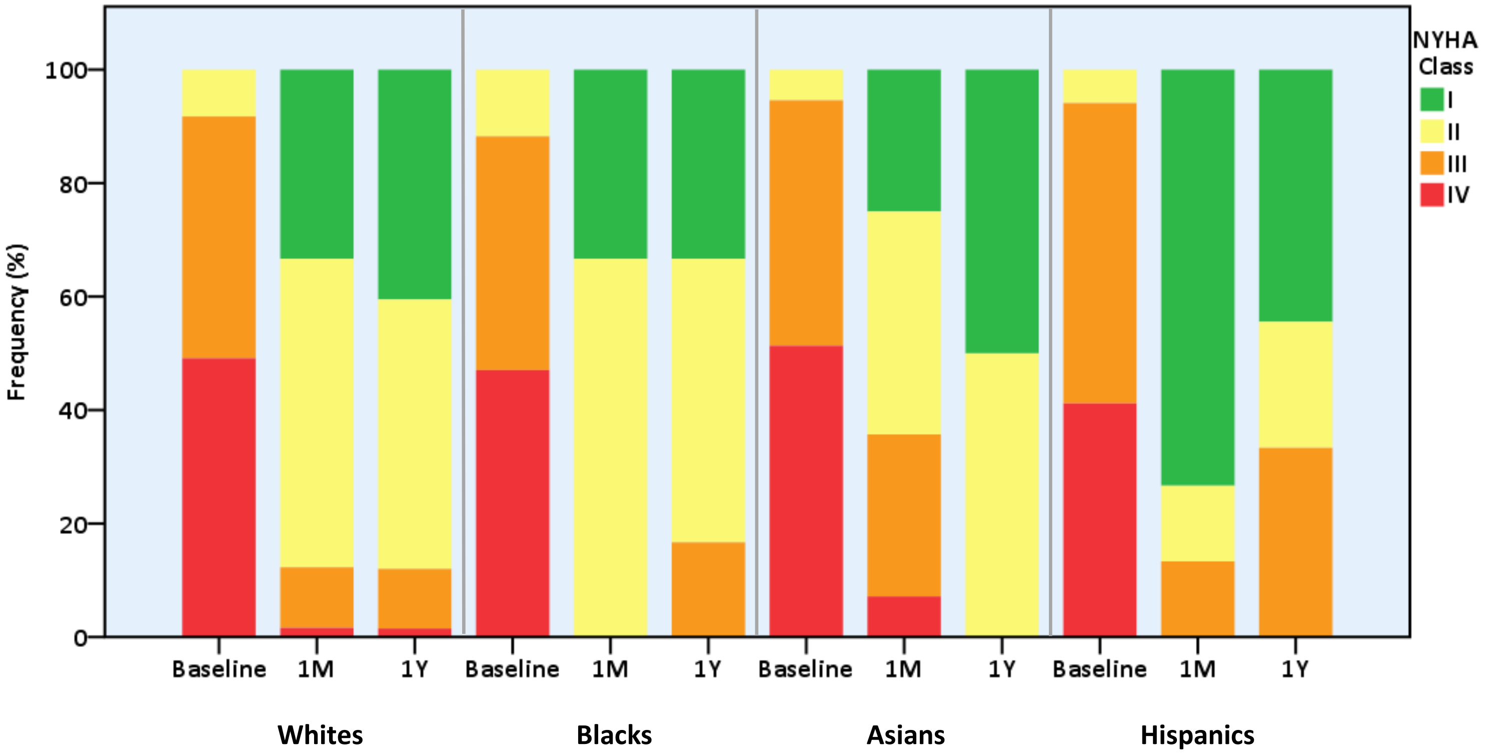

B Whites vs non-Whites

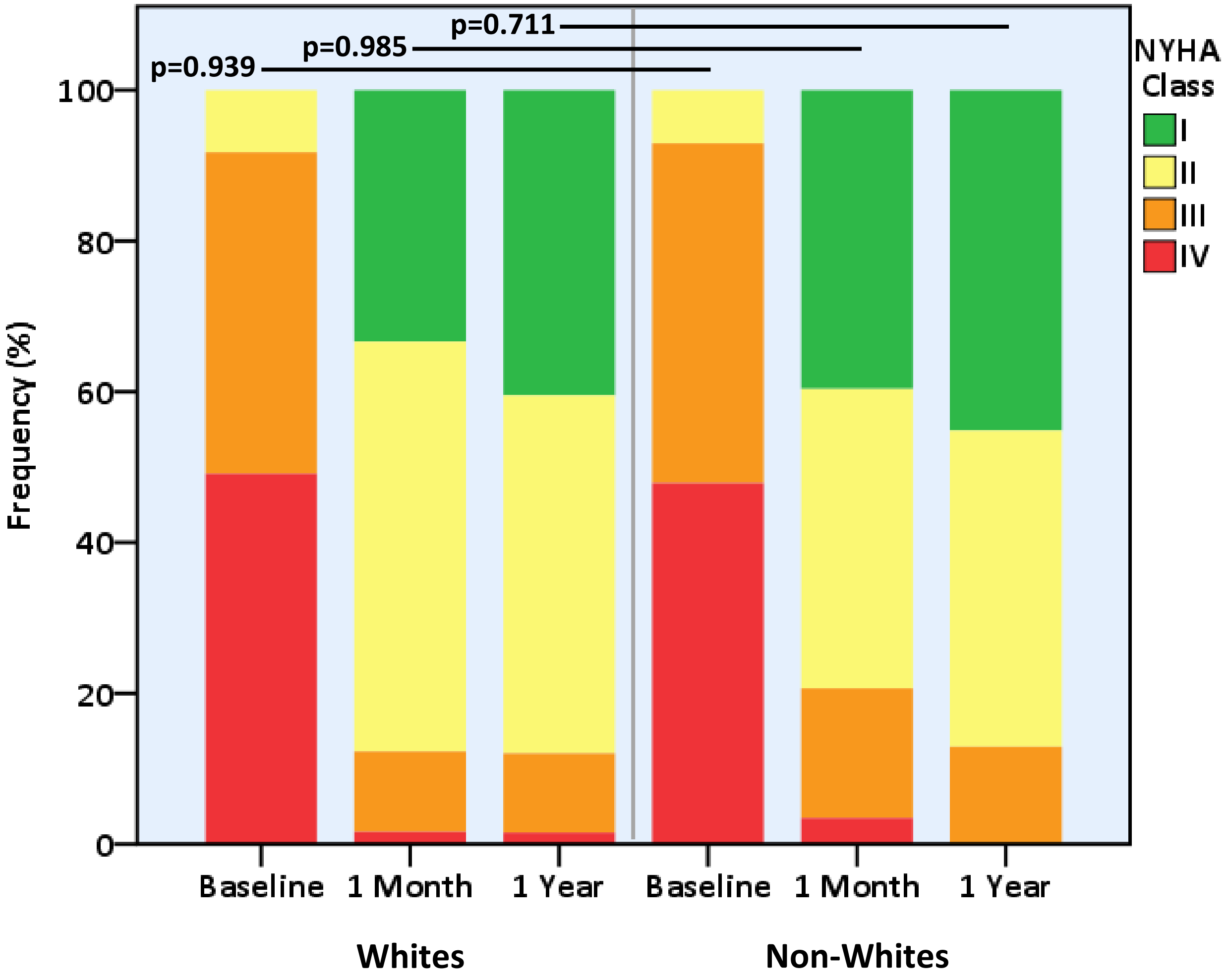

C Blacks vs non-Blacks

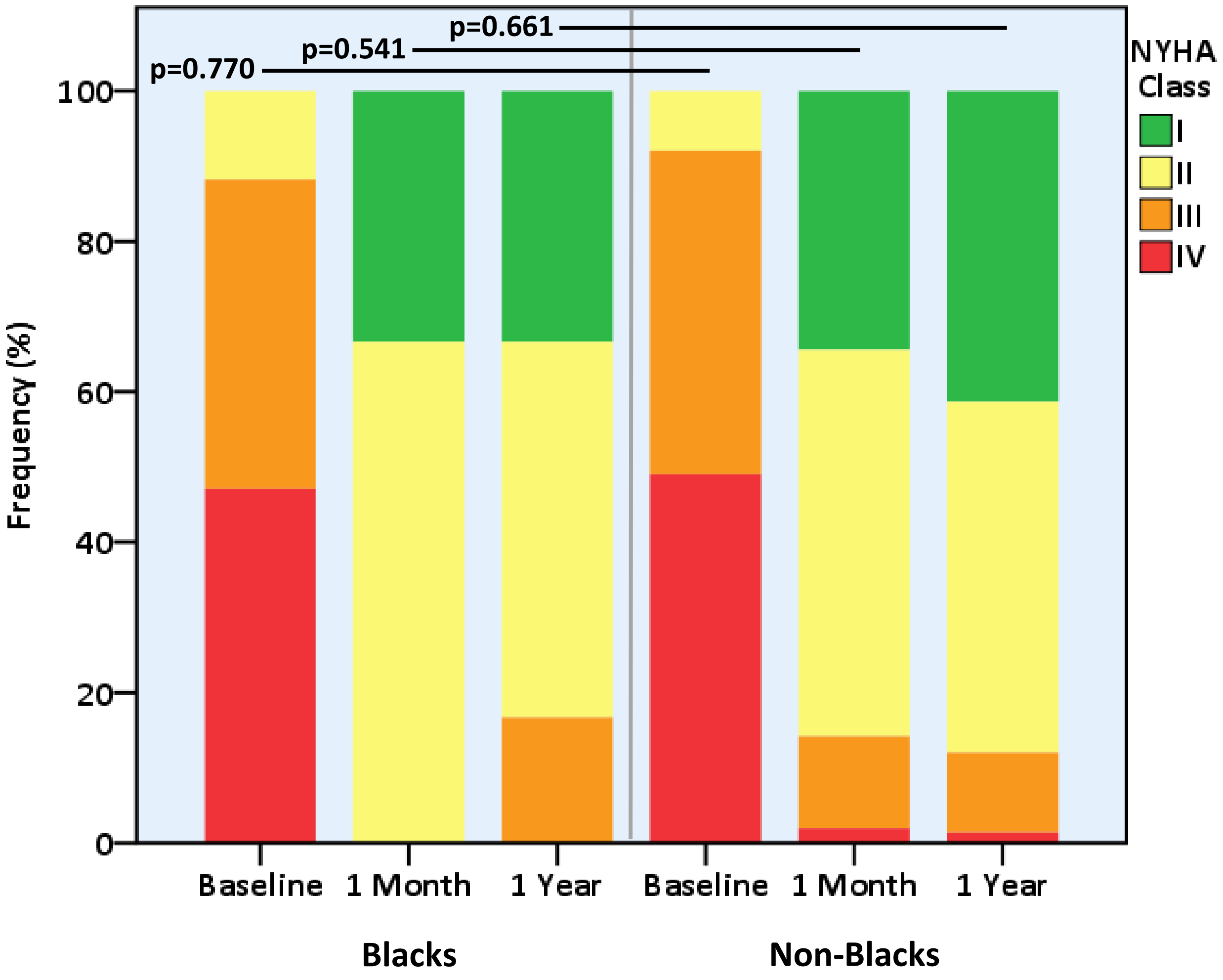

NYHA = New York Heart Association

Supplemental Figure 12. Mitral Regurgitation Grade at Baseline and Following Transcatheter Edge-to-Edge Repair for Primary Mitral Regurgitation According to Race

A All Races

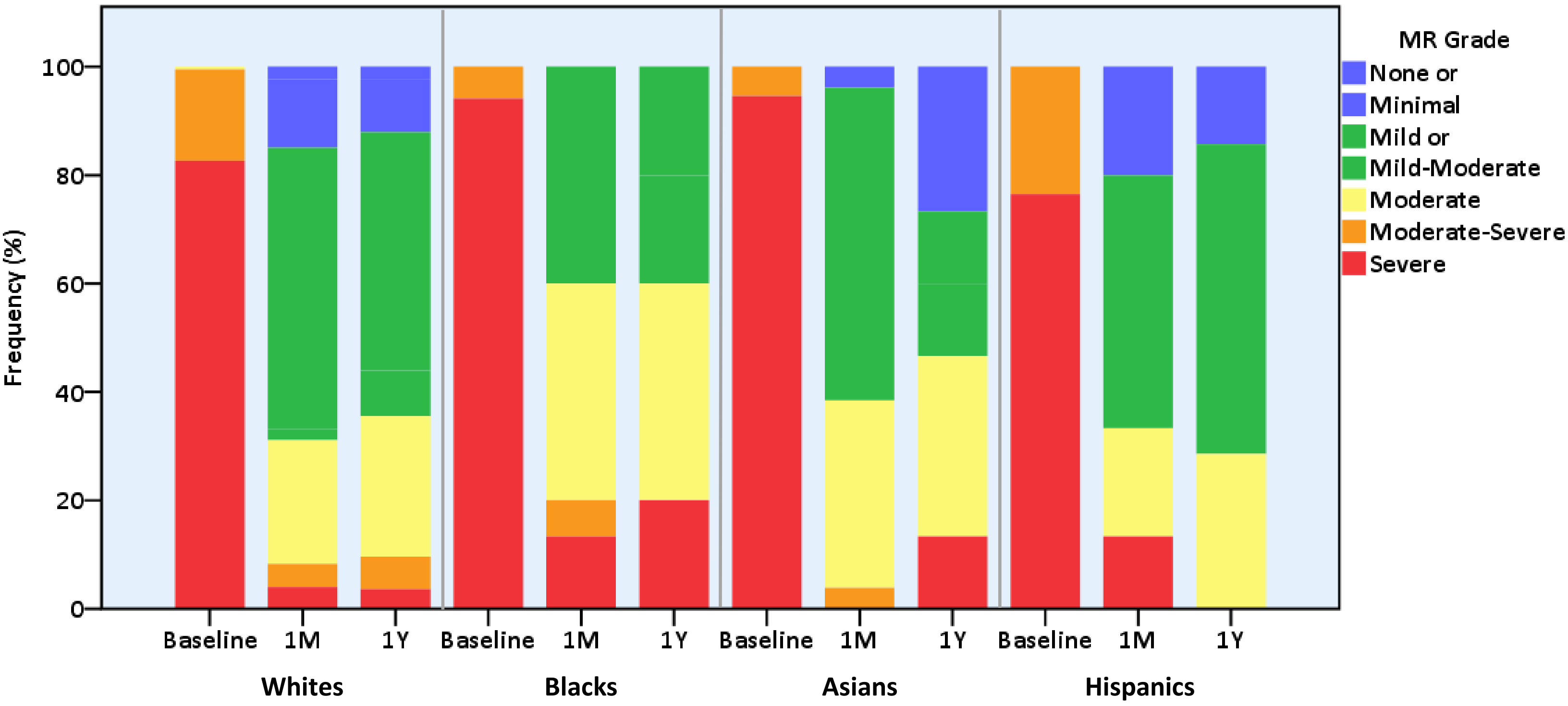

B Whites vs non-Whites

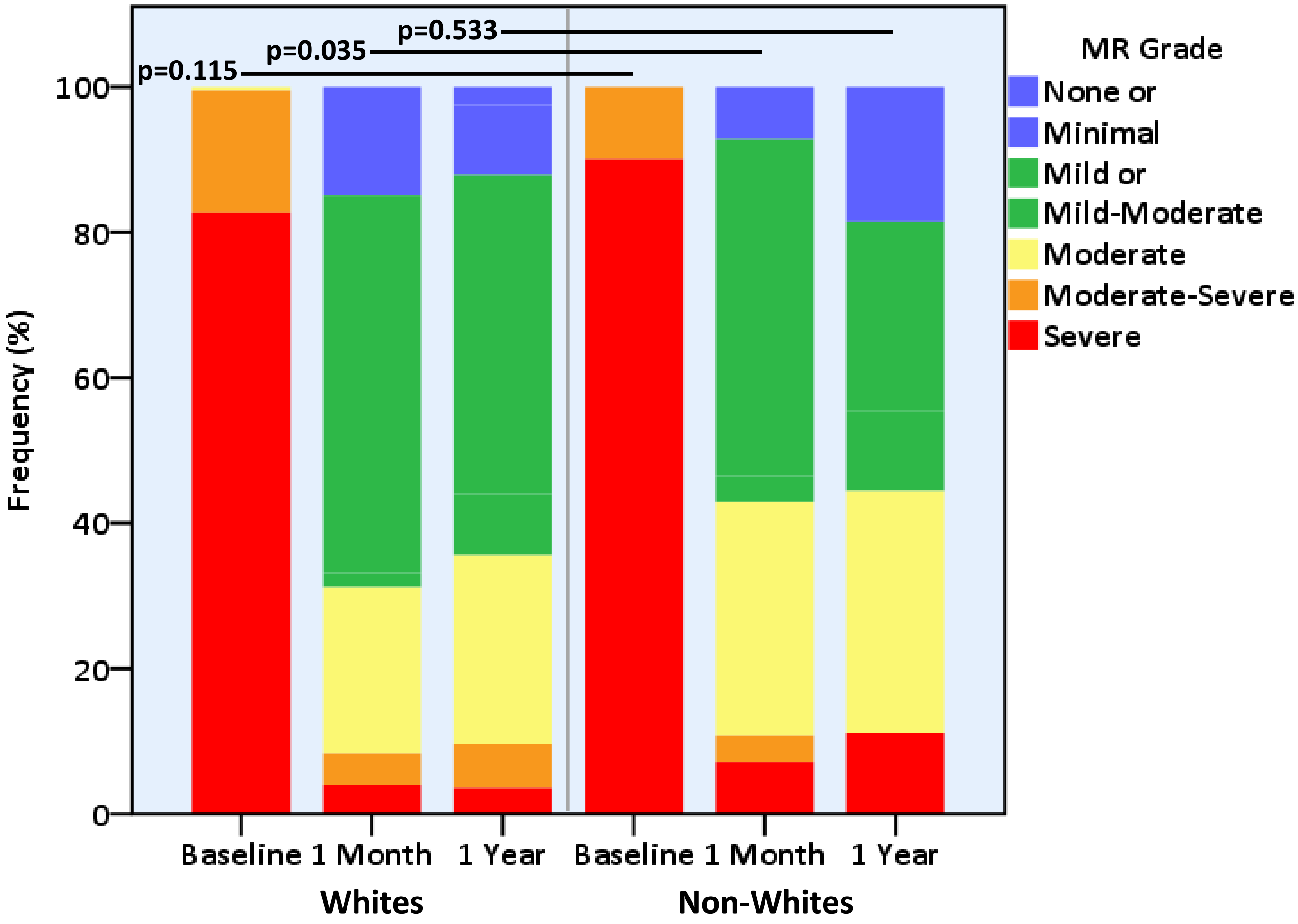

C Blacks vs non-Blacks

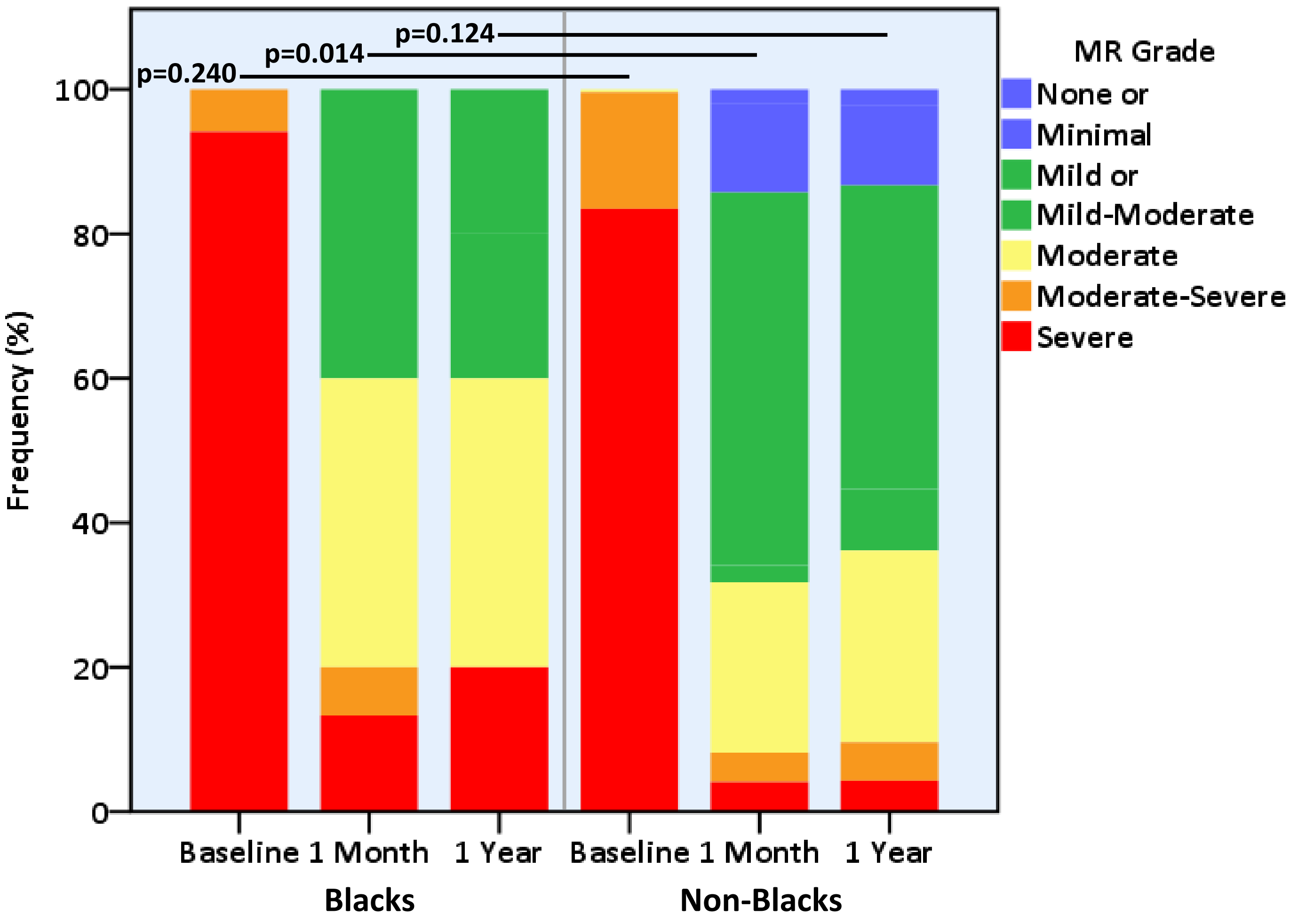

MR = mitral regurgitation

Supplemental Table 11. Baseline Clinical Characteristics of the Matched Cohorts According to Race

	Whites (N=147)	Non-Whites (N=147)	Blacks (N=65)	Non-Blacks (N=65)	P-Value Whites vs Non-Whites	P-Value Blacks vs Non-Blacks
**Demographic Details**						
Age (years)	75 (64-81)	72 (64-80)	66 (58-77)	73 (59-80)	0.286	0.203
Sex Male	73 (49.7)	70 (47.6)	39 (60.0)	38 (58.5)	0.726	0.858
Insurance						
None	2 (1.4)	1 (0.7)	0 (0.0)	1 (1.5)	0.562	1.000
Low-Income	33 (22.4)	37 (25.2)	15 (23.1)	12 (18.5)	0.584	0.517
Regular / Full	112 (76.2)	109 (74.1)	50 (76.9)	52 (80.0)	0.685	0.670
Median Yearly Household Income* (K USD)	67.1 (57.2-85.8)	64.4 (53.9-88.9)	57.1 (50.0-68.5)	61.9 (49.3-80.4)	0.733	0.237
Percentage of Adults with Academic Degree*	29.7 (17.5-44.5)	27.3 (18.7-44.2)	23.0 (15.1-32.1)	28.4 (20.0-38.4)	0.877	0.064
**Comorbidities**						
Obesity (Body Mass Index ≥30kg/m^2)	28 (19.0)	28 (19.0)	16 (24.6)	11 (16.9)	1.000	0.280
Diabetes Mellitus	36 (24.7)	59 (40.1)	26 (40.0)	19 (29.2)	**0.005**	0.197
Hypertension	119 (81.0)	125 (85.0)	57 (87.7)	53 (81.5)	0.352	0.331
Smoking History	13 (8.8)	5 (3.4)	3 (4.6)	6 (9.2)	0.052	0.492
Previous MI, PCI, or CABG	67 (45.6)	64 (43.5)	22 (33.8)	29 (44.6)	0.725	0.209
Prior Stroke or Transient Ischemic Attack (TIA)	13 (8.8)	26 (17.7)	10 (15.4)	12 (18.5)	**0.025**	0.640
Peripheral Arterial Disease (PAD)	12 (8.2)	14 (9.5)	6 (9.2)	4 (6.2)	0.681	0.510
Atrial Fibrillation / Flutter	69 (46.9)	63 (42.9)	24 (36.9)	32 (49.2)	0.482	0.157
Chronic Obstructive Pulmonary Disease (COPD)	25 (17.0)	23 (15.6)	14 (21.5)	15 (23.1)	0.752	0.833
Anemia+	89 (60.5)	101 (68.7)	47 (72.3)	44 (67.7)	0.143	0.566
Stage ≥III Chronic Kidney Disease	108 (73.5)	110 (74.8)	45 (69.2)	44 (67.7)	0.790	0.850
**Heart Failure Indices**						
New York Heart Association (NYHA) Class						
II	5 (3.4)	5 (3.4)	1 (1.5)	4 (6.2)	1.000	0.365
III	62 (42.2)	58 (39.5)	23 (35.4)	23 (35.4)	0.635	1.000
IV	80 (54.4)	84 (57.1)	41 (63.1)	38 (58.5)	0.639	0.590
Kansas City Cardiomyopathy Questionnaire 12 Score	34.4 (15.6-56.3)	29.2 (12.5-53.1)	32.3 (12.5-50.5)	32.8 (11.5-51.8)	0.488	0.751
6-Minute Walk Test Distance (m)	221 (122-366)	207 (91-312)	152 (46-320)	274 (69-358)	0.227	0.452

Serum B-type Natriuretic Peptide (pg/mL)	797 (313-2,036)	935 (324-2,083)	1,279 (554-2,164)	1,249 (491-2,586)	0.442	0.636
**Risk Status**						
STS Score for Mitral Valve Repair	5.3 (2.8-8.7)	6.2 (2.8-10.2)	6.7 (2.5-10.2)	5.2 (2.5-10.0)	0.537	0.701
MitraScore	3 (2-4)	3 (3-4)	4 (3-4)	4 (2-5)	0.518	0.803
**Treatment**						
Medications						
Beta Blockers	95 (64.6)	107 (72.8)	48 (73.8)	49 (75.4)	0.131	0.840
Renin Angiotensin System (RAS) Inhibitors	71 (48.3)	73 (49.7)	34 (52.3)	30 (46.2)	0.816	0.483
Mineralocorticoid Receptor Antagonists (MRAs)	29 (19.7)	39 (26.5)	19 (29.2)	18 (27.7)	0.167	0.846
Loop Diuretics						
Frequency	113 (76.9)	116 (78.9)	52 (80.0)	46 (70.8)	0.673	0.222
Furosemide-Equivalent Dose (mg/day)	40 (40-80)	40 (40-80)	40 (40-80)	40 (25-80)	0.718	0.478
Anti-Arrhythmics	40 (27.2)	32 (21.8)	16 (24.6)	12 (18.5)	0.278	0.393
Hydralazine + Nitrates	4 (2.7)	8 (5.5)	6 (9.4)	3 (4.6)	0.233	0.324
Oral Anticoagulants	62 (42.2)	61 (41.5)	27 (41.5)	28 (43.1)	0.906	0.859
Cardiac Implantable Electronic Device (CIED)						
Total	52 (35.4)	53 (36.1)	35 (53.8)	30 (46.2)	0.903	0.380
Pacemaker	14 (9.5)	8 (5.4)	4 (6.2)	6 (9.2)	0.184	0.510
Implantable Cardioverter Defibrillator (ICD)	6 (4.1)	17 (11.6)	15 (23.1)	8 (12.3)	**0.017**	0.108
CRT/Defibrillator (CRT/D)	32 (21.8)	28 (19.0)	16 (24.6)	16 (24.6)	0.563	1.000

Data are presented as number (percentage) or median (interquartile range), where appropriate.

* Per zip code

+ Anemia was defined as a blood hemoglobin of <13mg/dL in men or <12mg/dL in women.

CABG = coronary bypass artery grafting; CRT = cardiac resynchronization therapy; GFR = glomerular filtration rate; MI = myocardial infarction; PCI = percutaneous coronary intervention; STS = Society of Thoracic Surgeons; USD = United States Dollars

Supplemental Table 12. Baseline Echocardiographic Data of the Matched Cohorts According to Race

					P-Value	
	**Whites (N=147)**	**Non-Whites (N=147)**	**Blacks (N=65)**	**Non-Blacks (N=65)**	**Whites vs Non-Whites**	**Blacks vs Non-Blacks**
**Mitral Valve**						
Mitral Regurgitation Etiology					0.329	0.824
Functional	91 (61.9)	99 (67.3)	53 (81.5)	52 (80.0)		
Primary	56 (38.1)	48 (32.7)	12 (18.5)	13 (20.0)		
Mitral Regurgitation Severity					0.878	0.366
Moderate-Severe	26 (17.7)	25 (17.0)	10 (15.4)	14 (21.5)		
Severe	121 (82.3)	122 (83.0)	55 (84.6)	51 (78.5)		
Mitral Regurgitation PISA EROA (cm^2)	0.37 (0.26-0.48)	0.32 (0.25-0.42)	0.34 (0.25-0.47)	0.35 (0.27-0.47)	0.116	0.641
Mitral Regurgitation PISA RVol (mL)	51.5 (34.3-68.7)	46.2 (35.9-61.7)	46.1 (33.9-60.9)	53.3 (35.4-68.1)	0.401	0.495
Transmitral Mean Pressure Gradient (TMPG) (mmHg)	3 (2-4)	3 (2-4)	3 (2-4)	2 (2-4)	0.793	0.270
≥Moderate Mitral Annulus Calcification (MAC)	12 (8.2)	10 (6.8)	4 (6.2)	2 (3.1)	0.658	0.680
**Left Heart**						
Left Ventricular Ejection Fraction (LVEF) (%)	45 (25-63)	40 (22-60)	27 (17-45)	28 (19-45)	0.329	0.665
Left Ventricular End-Systolic Diameter (LVESD) (cm)	3.9 (3.2-5.2)	4.5 (3.4-5.6)	5.3 (3.9-6.2)	4.8 (3.7-5.6)	**0.039**	**0.041**
Left Ventricular Mass Index, ASE Formula (gr/m^2)	127.7 (97.6-155.4)	132.1 (113.4-158.6)	132.1 (114.3-166.2)	123.2 (91.2-148.9)	0.069	**0.029**
Left Atrial Volume Index (LAVi) (cm^3/m^2)	56.3 (42.8-72.0)	54.8 (44.9-73.7)	50.2 (40.6-76.1)	46.9 (37.2-61.2)	0.582	0.061
**Right Heart**						
≥Moderate Right Ventricular Dysfunction	34 (28.6)	36 (27.1)	22 (38.6)	19 (35.8)	0.790	0.766
≥Moderate-Severe Tricuspid Regurgitation	34 (23.1)	54 (36.7)	23 (35.4)	21 (32.3)	**0.011**	0.711
**Right Ventricular (RV)-Pulmonary Arterial (PA) Coupling**						
Tricuspid Annular Plane Systolic Excursion (TAPSE) (mm)	16 (13-19)	16 (14-21)	16 (14-20)	16 (13-20)	0.261	0.437
Pulmonary Arterial Systolic Pressure (PASP) (mmHg)	48 (34-56)	50 (35-60)	54 (36-65)	48 (35-55)	0.106	**0.040**
TAPSE/PASP (mm/mmHg)	0.33 (0.24-0.54)	0.34 (0.25-0.52)	0.33 (0.23-0.49)	0.32 (0.26-0.51)	0.834	0.579

Data are presented as number (percentage) or median (interquartile range), where appropriate.

ASE = American Society of Echocardiography; EROA = effective regurgitant orifice area; PISA = proximal isovelocity surface area; RVol = regurgitant volume

Supplemental Table 13. Procedural Details and Results Observed in the Matched Cohorts According to Race

					P-Value	
	**Whites (N=147)**	**Non-Whites (N=147)**	**Blacks (N=65)**	**Non-Blacks (N=65)**	**Whites vs Non-Whites**	**Blacks vs Non-Blacks**
**Presentation to Procedure**						
Acute Decompensated Heart Failure	24 (16.3)	35 (23.8)	18 (27.7)	10 (15.4)	0.109	0.088
Cardiogenic Shock	8 (5.4)	4 (2.7)	3 (4.6)	2 (3.1)	0.238	1.000
Medical and/or Mechanical Hemodynamic Support	10 (6.8)	12 (8.2)	7 (10.8)	3 (4.6)	0.658	0.188
Urgent Procedure	41 (27.9)	32 (21.8)	20 (30.8)	18 (27.7)	0.224	0.700
**Procedural Aspects**						
Number of Clips Deployed						
0 (Aborted / Not Deployed)	6 (4.1)	1 (0.7)	1 (1.5)	1 (1.5)	0.121	1.000
1	53 (36.1)	59 (40.1)	15 (23.1)	29 (44.6)	0.471	**0.009**
2	69 (46.9)	64 (43.5)	34 (52.3)	30 (46.2)	0.558	0.483
≥2	88 (59.9)	87 (59.2)	49 (75.4)	35 (53.8)	0.905	**0.010**
≥3	19 (12.9)	23 (15.6)	15 (23.1)	5 (7.7)	0.505	**0.015**
Median	2 (1-2)	2 (1-2)	2 (2-2)	2 (1-2)	0.700	**0.003**
Device Generation						
1	26 (17.7)	32 (21.8)	12 (18.5)	11 (16.9)	0.379	0.818
2	57 (38.8)	58 (39.5)	23 (35.4)	26 (40.0)	0.905	0.587
3	48 (32.7)	33 (22.4)	19 (29.2)	22 (33.8)	0.050	0.571
4	16 (10.9)	24 (16.3)	11 (16.9)	6 (9.2)	0.174	0.193
Intervention Site						
A1P1	2 (1.4)	3 (2.0)	0 (0.0)	1 (1.5)	1.000	1.000
A2P2	143 (97.3)	142 (96.6)	64 (98.5)	64 (98.5)	1.000	1.000
A3P3	8 (5.4)	9 (6.1)	3 (4.6)	3 (4.6)	0.803	1.000
Total Duration (min)	109 (89-129)	108 (85-140)	114 (86-143)	104 (83-121)	0.644	0.054
Fluoroscopy Duration (min)	17 (13-24)	19 (13-30)	20 (14-31)	16 (13-22)	0.052	**0.033**
Conversion to Surgery	0 (0.0)	0 (0.0)	0 (0.0)	0 (0.0)	NA	NA
**Echocardiographic and Hemodynamic Effects**						
Mitral Regurgitation Severity Reduction to ≤Mild						
Immediately after Clip Deployment	98 (66.7)	105 (71.4)	45 (69.2)	52 (80.0)	0.377	0.158

At Discharge	113 (81.3)	102 (70.8)	45 (69.2)	48 (78.7)	**0.039**	0.228
Transmitral Mean Pressure Gradient (mmHg)						
Immediately after Clip Deployment	3 (2-4)	3 (2-4)	3 (2-4)	3 (2-4)	0.494	0.943
At 1-Month	4 (3-5)	4 (3-5)	5 (3-6)	4 (2-6)	0.695	0.211
Pulmonary Venous Flow Pattern Normalization on ≥1 Side	71 (55.5)	81 (64.3)	36 (65.5)	30 (50.8)	0.152	0.114
Delta V wave (mmHg)	-8 (-19-0)	-13 (-27-[-3])	-13 (-30-[-4])	-8 (-18-0)	**0.020**	**0.027**
Delta Mean Left Atrial Pressure (LAP) (mmHg)	-2 (-10-2)	-4 (-11-0)	-5 (-12-[-1])	-3 (-8-1)	**0.020**	0.124
Delta Mean Pulmonary Arterial Pressure (PAP) (mmHg)	1 (-5-4)	-3 (-8-4)	-1 (-8-4)	-4 (-6-3)	0.160	0.972
**Post-Procedural Course**						
Intensive Cardiac Unit (ICU) Stay Duration (hours)	20.0±74.9	25.1±124.3	55.0±193.6	18.9±83.3	0.672	0.171
Hospitalization Length (days)	1 (1-8)	2 (1-8)	5 (1-11)	1 (1-7)	0.596	**0.026**
Discharge Home	131 (93.6)	137 (94.5)	59 (93.7)	57 (89.1)	0.745	0.358
Blood Transfusion or Any 1-Month Non-Fatal Adverse Event*	18 (12.2)	24 (16.3)	14 (21.5)	5 (7.7)	0.317	**0.025**
**Medical Treatment at 1-Month**						
Beta Blockers	81 (76.4)	81 (65.9)	35 (64.8)	42 (76.4)	0.080	0.186
Renin Angiotensin System (RAS) Inhibitors	57 (54.8)	60 (49.6)	28 (52.8)	26 (48.1)	0.434	0.628
Mineralocorticoid Receptor Antagonists (MRAs)	26 (24.5)	28 (22.6)	13 (24.1)	14 (25.5)	0.728	0.867
Loop Diuretics	75 (70.8)	91 (73.4)	45 (83.3)	34 (61.8)	0.657	**0.012**
Anti-Arrhythmics	24 (22.9)	31 (25.4)	15 (28.3)	8 (14.5)	0.654	0.081
Hydralazine + Nitrates	4 (3.7)	8 (6.4)	6 (10.9)	3 (5.3)	0.345	0.317
Oral Anticoagulants	49 (46.2)	50 (40.3)	20 (37.0)	23 (41.8)	0.367	0.610

Data are presented as number (percentage), median (interquartile range), or mean±standard deviation, where appropriate.

* 1-month non-fatal adverse events included the following: tamponade, cardiac arrest, myocardial infarction, stroke, transient ischemic attack, MVARC bleeding, and vascular complications.

MVARC = mitral valve academy research consortium; NA = not applicable

Supplemental Table 14. Outcomes and Trends following Mitral Transcatheter Edge-to-Edge Repair in the Matched Cohorts According to Race

					P-Value	
	**Whites (N=147)**	**Non-Whites (N=147)**	**Blacks (N=65)**	**Non-Blacks (N=65)**	**Whites vs Non-Whites**	**Blacks vs Non-Blacks**
**Primary Outcome**						
All-Cause Mortality or Heart Failure Hospitalizations at 1-Year	36 (24.5)	50 (34.0)	29 (44.6)	17 (26.2)	0.073	**0.028**
Event-Free Survival Time at 1-Year (days)	285±12	269±12	234±19	289±17	0.203	**0.045**
**Secondary Outcomes**						
Clinical						
All-Cause Mortality at 1-Year	18 (12.2)	22 (15.0)	12 (18.5)	8 (12.3)	0.496	0.331
Heart Failure Hospitalizations at 1-Year	21 (14.3)	37 (25.2)	23 (35.4)	10 (15.4)	**0.019**	**0.009**
New York Heart Association Class ≤II						
At 1-Month	73 (75.3)	81 (73.0)	31 (67.4)	41 (80.4)	0.708	0.144
At 1-Year	48 (76.2)	47 (69.1)	12 (48.0)	19 (63.3)	0.365	0.254
Echocardiographic						
Mitral Regurgitation Severity ≤Mild						
At 1-Month	50 (55.6)	57 (55.3)	25 (56.8)	29 (59.2)	0.976	0.817
At 1-Year	18 (46.2)	26 (47.3)	11 (55.0)	10 (43.5)	0.915	0.451
Mitral Regurgitation Severity ≤Moderate						
At 1-Month	84 (96.6)	96 (95.0)	37 (88.1)	49 (100.0)	0.727	**0.018**
At 1-Year	37 (94.9)	50 (90.9)	18 (90.0)	23 (100.0)	0.695	0.210
Left Ventricular Mass Index (gr/m^2)						
At 1-Month	122.6 (97.6-153.1)	132.4 (110.0-157.2)	122.0 (108.4-168.1)	114.4 (93.9-146.5)	0.219	0.199
At 1-Year	112.7 (84.0-143.3)	124.0 (103.7-153.6)	125.3 (94.9-142.7)	115.7 (84.8-142.6)	0.143	0.487
Combined Clinical and Echocardiographic						
New York Heart Association Class ≤II or Mitral Regurgitation Severity ≤Mild						
At 1-Month	81 (90.0)	93 (86.9)	39 (84.8)	48 (94.1)	0.502	0.184
At 1-Year	51 (91.1)	51 (82.3)	16 (72.7)	22 (75.9)	0.163	0.799
**Trends**						
Absolute Change in New York Heart Association Class						

At 1-Month	-1.4±0.9	-1.4±0.8	-1.3±0.8	-1.5±0.9	0.769	0.231
At 1-Year	-1.5±0.9	-1.3±0.8	-1.0±0.7	-1.3±1.0	0.179	0.191
P-Value for 1-Year vs Baseline	**<0.001**	**<0.001**	**<0.001**	**<0.001**	NA	NA
Relative Change in Left Ventricular Mass Index (%)						
At 1-Month	3.7 (-16.6-19.1)	0.2 (-13.8-19.9)	3.3 (-12.7-21.8)	-0.8 (-14.3-26.5)	0.756	0.897
At 1-Year	-4.2 (-23.4-18.5)	-13.1 (-27.7-16.6)	-8.2 (-32.1-25.5)	15.0 (-20.4-29.9)	0.933	0.250

Data are presented as number (percentage), median (interquartile range), or mean±standard deviation, where appropriate.

NA = not applicable

Supplemental Table 15. Cox Proportional Hazard Model for the Combined Outcome of All-Cause Mortality or Heart Failure Hospitalizations at 1 Year Following Mitral Transcatheter Edge-to-Edge Repair in the Matched Cohorts

	**Whites vs Non Whites Matched Cohort**				**Blacks vs Non Blacks Matched Cohort**			
	**Univariable**		**Multivariable**		**Univariable**		**Multivariable**	
	**HR (95% CI)**	**P-Value**	**HR (95% CI)**	**P-Value**	**HR (95% CI)**	**P-Value**	**HR (95% CI)**	**P-Value**
**Baseline Clinical Variables**								
Age								
Continuous	0.99 (0.97-0.99)	0.034	0.98 (0.95-1.01)	0.106	1.01 (0.98-1.03)	0.652		
≥75 years	0.91 (0.60-1.39)	0.665			1.20 (0.66-2.19)	0.543		
Sex Male	1.14 (0.75-1.74)	0.547			0.77 (0.42-1.41)	0.398		
Race								
White vs non-White	0.76 (0.49-1.16)	0.205			0.63 (0.34-1.17)	0.144		
Black vs non-Black	2.05 (1.31-3.23)	0.002	1.84 (0.99-3.42)	0.055	1.83 (1.74-3.63)	0.019	1.73 (1.02-3.33)	**0.044**
Insurance								
None vs Low-Income or Regular / Full	6.47 (1.55-27.04)	0.011	7.05 (1.34-37.03)	**0.021**	0.05 (0.01-1.48)	0.762		
Low-Income vs Regular / Full	1.15 (0.71-1.87)	0.571			0.95 (0.46-1.96)	0.878		
None or Low-Income vs Regular / Full	0.79 (0.49-1.26)	0.323			1.09 (0.53-2.25)	0.822		
Median Yearly Household Income* (continuous)	1.09 (1.01-1.11)	0.026	1.01 (0.99-1.07)	0.577	1.02 (0.99-1.07)	0.703		
Percentage of Adults with Academic Degree* (continuous)	0.99 (0.98-1.02)	0.108			1.01 (0.99-1.03)	0.158		
Obesity	1.65 (1.02-2.66)	0.040	1.20 (0.60-2.40)	0.605	1.26 (0.65-2.43)	0.491		
Diabetes Mellitus	2.05 (1.34-3.15)	0.001	2.11 (1.18-3.76)	**0.011**	1.55 (0.86-2.81)	0.149		
Hypertension	1.01 (0.57-1.79)	0.973			1.66 (0.66-4.20)	0.286		
Smoking History	1.25 (0.54-2.86)	0.604			1.06 (0.33-3.43)	0.918		

Previous MI, PCI, or CABG	1.22 (0.80-1.86)	0.361			1.56 (0.87-2.78)	0.135		
Prior Stroke or Transient Ischemic Attack (TIA)	1.24 (0.71-2.16)	0.451			1.08 (0.52-2.24)	0.839		
Peripheral Arterial Disease (PAD)	1.50 (0.81-2.76)	0.195			0.64 (0.20-2.06)	0.454		
Atrial Fibrillation / Flutter	1.14 (0.75-1.75)	0.535			1.22 (0.69-2.18)	0.494		
Chronic Obstructive Pulmonary Disease (COPD)	1.61 (0.97-2.65)	0.064	1.36 (0.67-2.77)	0.395	2.01 (1.09-3.74)	0.027	2.46 (1.21-5.01)	**0.013**
Anemia+	3.02 (1.73-5.28)	<0.001	1.42 (0.67-2.97)	0.360	6.30 (2.25-17.62)	<0.001	4.24 (1.37-13.09)	**0.012**
Stage ≥III Chronic Kidney Disease	1.45 (0.86-2.44)	0.162			1.35 (0.71-2.56)	0.364		
Non-Ischemic Cardiomyopathy	1.54 (0.96-2.38)	0.052	1.30 (0.72-3.23)	0.375	1.37 (0.77-2.44)	0.279		
New York Heart Association (NYHA) Class								
III-IV	1.71 (0.42-6.96)	0.453			21.78 (0.06-79.63)	0.306		
IV	1.83 (1.17-2.86)	0.008	1.39 (0.74-2.62)	0.306	1.53 (0.83-2.84)	0.175		
Serum B-type Natriuretic Peptide Level (continuous)	1.09 (1.03-1.10)	<0.001	1.00 (0.99-1.01)	0.135	1.10 (1.02-1.11)	0.002	1.07 (1.01-1.08)	**0.018**
No Use of Renin Angiotensin System (RAS) Inhibitors	1.67 (1.08-2.58)	0.021	1.97 (1.12-3.48)	**0.019**	2.01 (1.11-3.67)	0.022	2.07 (1.04-4.11)	**0.038**
No Use of Mineralocorticoid Receptor Antagonists (MRAs)	1.15 (0.69-1.94)	0.589			2.14 (0.99-4.58)	0.051	1.20 (0.48-2.97)	0.695
Furosemide-Equivalent Dose (continuous)	1.06 (1.02-1.10)	0.001	1.02 (0.99-1.07)	0.539	1.02 (0.99-1.07)	0.411		
Hydralazine + Nitrates Prescription	1.09 (0.34-3.44)	0.890			0.92 (0.22-3.79)	0.904		
Oral Anticoagulants Prescription	0.75 (0.49-1.17)	0.204			0.91 (0.51-1.64)	0.764		
Cardiac Implantable Electronic Device (CIED)								
Any	1.87 (1.22-2.85)	0.004	1.48 (0.75-2.93)	0.262	1.46 (0.81-2.63)	0.207		
Cardiac Resynchronization Therapy/Defibrillator (CRT/D)	1.46 (0.90-2.35)	0.122			1.31 (0.69-2.48)	0.416		
**Baseline Echocardiographic Variables**								
Functional Mitral Regurgitation	3.57 (1.98-6.44)	<0.001			2.06 (0.81-5.21)	0.128		
Severe Mitral Regurgitation	1.47 (0.80-2.71)	0.216			2.29 (0.90-5.80)	0.081	1.07 (0.39-2.94)	0.888

Mitral Regurgitation PISA EROA								
Continuous	2.72 (0.68-7.69)	0.185			1.89 (0.32-11.11)	0.481		
≥0.40cm^2	1.18 (0.74-1.85)	0.489			1.04 (0.55-1.89)	0.957		
Transmitral Mean Pressure Gradient (TMPG) (continuous)	0.93 (0.81-1.07)	0.294			1.05 (0.89-1.23)	0.599		
≥Moderate Mitral Annulus Calcification (MAC)	0.76 (0.31-1.86)	0.543			1.24 (0.30-5.12)	0.768		
Left Ventricular Ejection Fraction (LVEF)								
Continuous	0.98 (0.95-0.99)	<0.001	1.02 (0.99-1.05)	0.196	0.98 (0.96-0.99)	0.022	0.97 (-.93-1.01)	0.140
<60%	2.76 (1.55-4.89)	0.001			3.17 (0.98-10.21)	0.054		
<40%	1.98 (1.29-3.05)	0.002			1.71 (0.89-3.31)	0.109		
Left Ventricular End-Systolic Diameter (LVESD)								
Continuous	1.31 (1.15-1.50)	<0.001	1.00 (0.69-1.45)	0.999	1.21 (0.97-1.50)	0.087	1.37 (0.80-2.35)	0.249
≥0.4cm	1.82 (1.17-2.84)	0.008			1.37 (0.70-2.70)	0.363		
Left Ventricular Mass Index, ASE Formula (continuous)	1.02 (0.99-1.07)	0.379			1.01 (0.99-1.08)	0.840		
Left Atrial Volume Index (LAVi)								
Continuous	1.02 (0.99-1.09)	0.531			0.99 (0.99-1.01)	0.897		
>60cm^3/m^2	1.05 (0.69-1.62)	0.815			0.84 (0.44-1.60)	0.598		
≥Moderate Right Ventricular Dysfunction	1.96 (1.22-3.15)	0.005	1.24 (0.67-2.31)	0.491	1.42 (0.75-2.68)	0.287		
≥Moderate-Severe Tricuspid Regurgitation	1.74 (1.13-2.68)	0.012	1.18 (0.64-2.19)	0.599	1.99 (1.11-3.55)	0.021	1.07 (0.51-2.24)	0.861
TAPSE/PASP								
Continuous	0.19 (0.06-0.63)	0.006			0.18 (0.04-0.86)	0.032		
≤0.37mm/mmHg (total cohort median)	2.26 (1.41-3.63)	0.001	2.56 (1.23-5.41)	**0.013**	2.04 (1.09-3.82)	0.027	1.36 (1.12-2.98)	**0.044**
**Procedural Variables**								
Acute Heart Failure Presentation, Cardiogenic Shock, Hemodynamic Support, or Urgent Procedure	4.46 (2.88-6.90)	<0.001	2.71 (1.50-4.90)	**0.001**	3.99 (2.17-7.35)	<0.001	2.53 (1.24-5.14)	**0.010**
Number of Clips Deployed								

Continuous	1.46 (1.12-1.92)	0.005	1.35 (0.90-2.03)	0.147	2.06 (1.40-3.03)	<0.001	1.67 (1.04-2.67)	**0.033**
≥2	1.88 (1.18-3.00)	0.008			2.82 (1.36-5.85)	0.005		
≥3	1.45 (0.84-2.50)	0.179			2.56 (1.32-4.96)	0.005		
Use of 1st Generation Device	0.73 (0.41-1.29)	0.275			0.79 (0.35-1.77)	0.567		

* Per zip code

+ Anemia was defined as a blood hemoglobin of <13mg/dL in men or <12mg/dL in women.

ASE = American Society of Echocardiography; CABG = coronary artery bypass grafting; CI = confidence interval; EROA = effective regurgitant orifice area; HR = hazard ratio; MI = myocardial infarction; NA = not applicable; PASP = pulmonary arterial systolic pressure; PCI = percutaneous coronary intervention; PISA = proximal isovelocity surface area; TAPSE = tricuspid annular plane systolic excursion
